# Electrochemical Site-Selective Alkylation of Azobenzenes with (Thio)Xanthenes

**DOI:** 10.3390/molecules27154967

**Published:** 2022-08-04

**Authors:** Qiang Zhong, Hui Gao, Pei-Long Wang, Chao Zhou, Tao Miao, Hongji Li

**Affiliations:** 1 Key Laboratory of Green and Precise Synthetic Chemistry and Applications, Ministry of Education, School of Chemistry and Materials Science, Huaibei Normal University, Huaibei 235000, China; 2 Key Laboratory for Chemistry and Molecular Engineering of Medicinal Resources, School of Chemistry and Pharmaceutical Sciences, Guangxi Normal University, Guilin 541004, China; 3 Information College, Huaibei Normal University, Huaibei 235000, China

**Keywords:** electrochemistry, azobenzenes, xanthenes, thioxanthenes, alkylation, C–H functionalization

## Abstract

Herein, we first report an electrochemical methodology for the site-selective alkylation of azobenzenes with (thio)xanthenes in the absence of any transition metal catalyst or external oxidant. A variety of groups are compatible with this electrochemical alkylation, which furnishes the products in moderate to good yields.

## 1. Introduction

Azobenzenes are a class of unique aromatic compounds that have been broadly applied in numerous fields, including biomedicine, solar thermal fuels and organic synthesis [1,2,3]. The azo unit is always considered as a privileged scaffold in the design of polymer and chiral catalysts as it readily undergoes cis/trans isomerization upon irradiation under UV or visible light [4,5,6,7,8]. Particularly, azobenzenes have also received increasing attention because of their powerful ability to manipulate organic molecules in synthetic chemistry. Accordingly, various synthetic methods that provide access to, and direct functionalization of, azobenzenes have become an area of interest within the fields of organic synthesis [9,10,11].

**Scheme 1:**
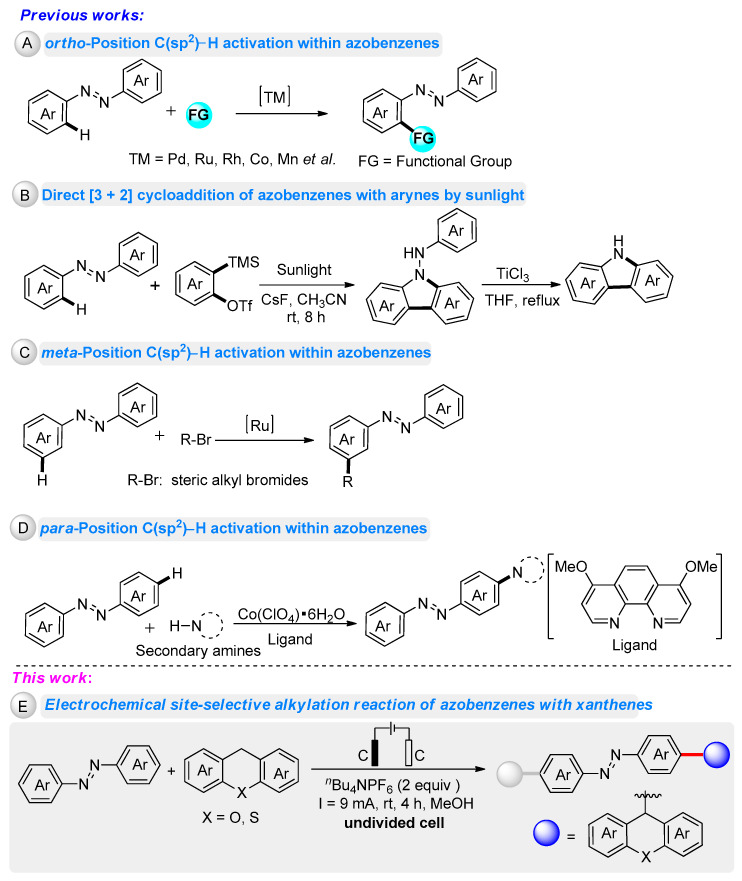
Strategies for the C–H functionalization of azobenzenes.

To the best of our knowledge, transition metal-catalyzed inert C–H activation, assisted by a directing group, is the most reliable method for chemical bond formation, and has proven to be indispensable for organic synthesis [12,13,14]. More specifically, azobenzenes, containing an “N=N” unit, can readily coordinate with a suitable transition metal, such as Pd, Ru, Rh, Co, Mn and some others, enabling the activation and late-stage functionalization of an *ortho*-position C–H bond (Scheme 1A) [15,16,17,18,19,20,21,22,23,24,25]. For example, our group and Ellman’s group early in 2013 completed indazole synthesis from the inert ortho C–H activation of azobenzenes enabled by Pd and Rh catalysis, respectively [16,17]. A number of excellent works on the functionalization of azobenzenes have been reported, using the same strategies. Recently, our group found that aryne chemistry could realize the mild transformation of azobenzenes into carbazole derivatives under sunlight irradiation, which bypasses the use of toxic transition metal catalysts and oxidants (Scheme 1B) [26]. Most precedents mainly focus on the *ortho*-C–H, while those involving meta- or para-position C–H functionalization remain relatively scarce [27,28]. Yang, Li and coworkers in 2017 reported Ru-catalyzed C_Ar_–H (di)alkylation reactions of azobenzenes with various types of alkyl bromides, in which *meta-*/*ortho*-selectivity could be well controlled and achieved (Scheme 1C) [27]. Furthermore, advancement on the *para*-position C–H activation and functionalization of azobenzenes has just been achieved. Very recently, Su’s group first reported a cobalt-catalyzed *para*-selective amination of azobenzenes with a variety of secondary amine compounds, in which the presence of a ligand is crucial for the transformation (Scheme 1D) [28]. Remarkably, most of the previously reported works on the functionalization of azobenzenes suffered from the use of transition metal catalysts, toxic oxidants and high reaction temperatures, which have severely restricted their further application in synthetic chemistry. Currently, the development of a simple and mild method for the diverse functionalization of azobenzenes is highly desirable.

In recent years, electrochemical synthesis has received increasing attention for its powerful ability to forge chemical bonds, presumably due to the advantages of no external stoichiometric chemical oxidants or reductants and milder conditions over the conventional approaches [29,30,31,32,33,34,35,36,37,38,39,40]. As a result, we speculate that electrochemistry maybe provides a unique opportunity to facilitate the functionalization of azobenzene. In a recent study, we disclosed an electrochemical formal [3 + 2] cycloaddition of azobenzenes with hexahydro-1,3,5-triazines, which afforded 1,2,4-triazolidine derivatives in an efficient fashion [41]. Based on these works, and our recent findings in electrochemical synthesis [41,42,43], we continue our effort to address the problem of *para*-position C–H functionalization in the azobenzenes with the electrochemical method. Herein, we report a catalyst-free alkylation of azobenzenes with (thio)xanthenes enabled by electrochemistry, which affords a series of azobenzenes derivatives with high regioselectivity (Scheme 1E).

## 2. Results and Discussion

Initially, (*E*)-1,2-Diphenyldiazene (**1a**) and xanthene (**2a**) were chosen as the model substrates to optimize the reaction conditions for the electrochemical alkylation reaction (Table 1). The reaction system was conducted with two carbon rods as the anode and cathode, ^*n*^Bu_4_NPF_6_ as an electrolyte, MeOH as a solvent, at constant current of 9 mA and room temperature for 4 h, generating the desired product **3a** in 76% yield (Table 1, entry 1). Meanwhile, the faradaic efficiency for the electrochemical alkylation of azobenzene was determined as 33.9% (For details, see the electronic Supporting Information). Replacing the electrolyte ^*n*^Bu_4_NPF_6_ with some other commonly used electrolytes, such as ^*n*^Bu_4_NBF_4_, ^*n*^Bu_4_NI and LiClO_4_, led to the formation of **3a** in decreasing yields (entries 2–4). It was found that choice of electrode materials proved to be crucial for this alkylation reaction. Employment of Pt(+)|Pt(−) as an electrode did not promote the model reaction (entry 5). Lower yields of **3a** were obtained when carbon with Pt was used as either the anode or cathode (Table 1, entries 6 and 7). Graphite felt (GF) or Ni electrodes could not improve the yield (entries 8–10). Next, a variety of solvents, including DCE, CH_3_CN, THF, DMF and acetone, were screened, and the result showed that MeOH was the best solvent (entries 11–15). Decreasing or increasing the reaction time did not improve the yield of **3a** (entries 16–17). Subsequently, changing the intensity of constant current from 8 mA to 10 mA also failed to enhance the yield of **3a** (entries 18–19). The control experiment demonstrated that the reaction could not proceed without electric current (entry 20). Furthermore, the reaction performed under N_2_ atmosphere had no obvious effect on the yield of **3a** (entry 21).

**Scheme 2:**
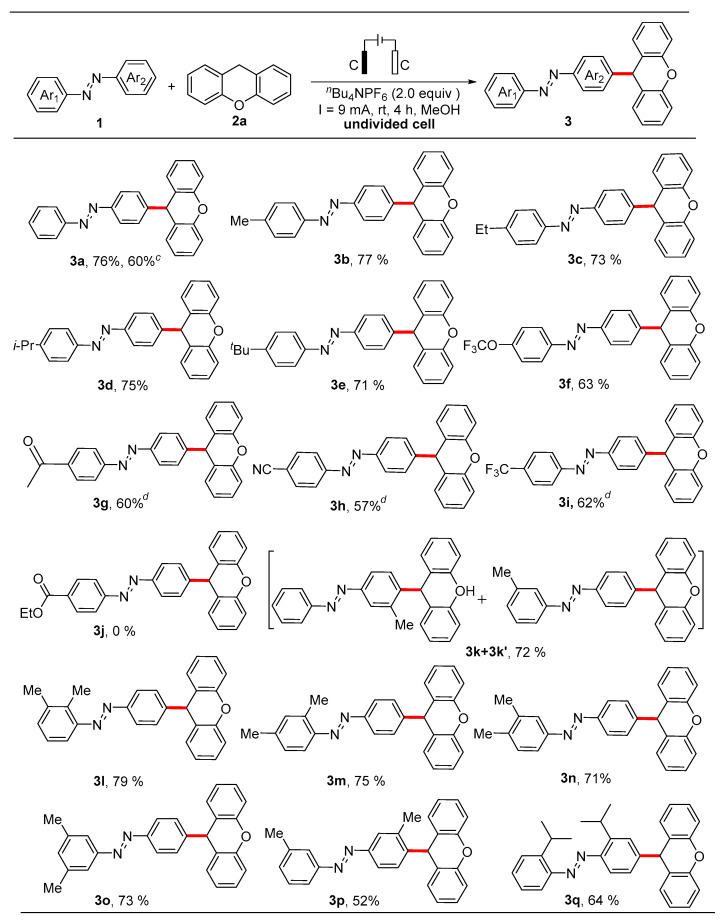
Scope of azobenzenes *^a,b,c^*. *^a^* Reaction conditions: **1** (0.30 mmol), **2a** (0.36 mmol), *^n^*Bu_4_NPF_6_ (2.0 equiv), MeOH (5.0 mL), carbon rod anode (Φ 6 mm), carbon rod cathode (Φ 6 mm), rt, 4 h (Q = 4.48 F mol^−1^). *^b^* Isolated yields are shown. *^c^* Gram-scale synthesis. *^d^* 8 h.

With the established optimal reaction conditions, we set out to investigate the substrate scope of azobenzenes (Scheme 2). In general, azobenzenes bearing electron-donating and electron-withdrawing groups are well compatible with this reaction. First, a variety of mono-substituted azobenzenes were examined under the optimized conditions. For the 4-substituted azobenzenes, the reaction took place specifically on the 4’-position (**3b**–**3i**). Alkyl substituents, including Me, Et, *i*-Pr and *t*-Bu, were well tolerated in the electrochemical system, and generated the desired product **3b**–**3e** in good yields. Gratifyingly, we further determined the exact structure of **3d** by single-crystal analysis [44]. In addition, we found that incorporation of OCF_3_ on the 4-position of azobenzene gave the product **3f** a 63% yield. Azobenzenes bearing strong electron-withdrawing groups, such as acetyl, cyano and trifluoromethyl group, could interact well with xanthene to form the products **3g**–**3i** in moderate yields by prolonging reaction time, which indicated that the electron-withdrawing group could reduce the reactivity of the substrates. Unfortunately, introduction of an ester group failed to cause a reaction with xanthene (**3j**). For the 3-substituted azobenzene, the reaction randomly happened on both the 4-positon and the 4’-position of the aromatic ring, affording the mixture of **3k** and **3k’** in 72% total yield. Next, we examined a variety of disubstituted azobenzenes bearing 2,3-dimethyl,2,4-dimethyl,3,4-dimethyl and 3,5-dimethyl substituents, and all of them worked well under the reaction conditions to form the corresponding products **3l**–**3o** in good yields and regioselectivity. Notably, the alkylation reaction selectively happened on the 4’-position of these disubstituted unsymmetrical azobenzenes. In addition, some symmetrical azobenzenes were also tested. It was found that both (*E*)-1,2-di-*m*-tolyldiazene and (*E*)-1,2-bis(2-isopropylphenyl)diazene proceeded smoothly to generate the products **3p**–**3q** in moderate yields.

**Scheme 3:**
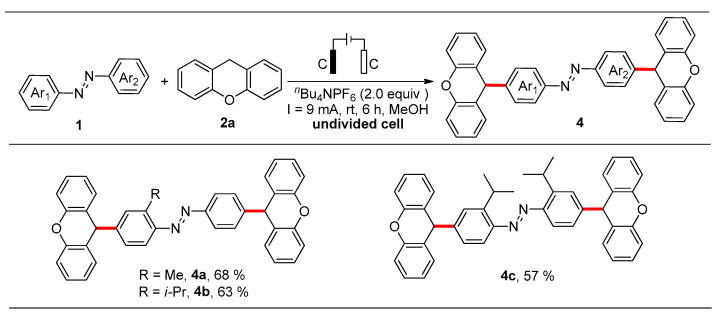
Dialkylation of azobenzenes with xanthene **2a** *^a,b^*. *^a^* Reaction conditions: **1** (0.30 mmol), **2a** (0.66 mmol), *^n^*Bu_4_NPF_6_ (2.0 equiv), MeOH (5.0 mL), carbon rod anode (Φ 6 mm), carbon rod cathode (Φ 6 mm), rt, 6 h (Q = 6.72 F mol^−1^). *^b^* Isolated yields are shown.

We next continued to explore the dialkylation of azobenzenes with xanthene **2a** (Scheme 3). By increasing the amount of **2a** to 2.2 equivalents and prolonging the reaction time to 6 h, the dialkylation of azobenzenes proceeded well under the modified reaction conditions. For instance, some unsymmetrical azobenzenes, bearing 2-Me and 2-*i*Pr substituents, reacted with xanthene to generate the corresponding products **4a** and **4b** in 68% and 63% yields, respectively. Additionally, we also found that symmetrical azobenzene (*E*)-1,2-bis (2-isopropylphenyl)diazene was demonstrated to be a suitable substrate and resulted in the formation of **4c** in 57% yield.

**Scheme 4:**
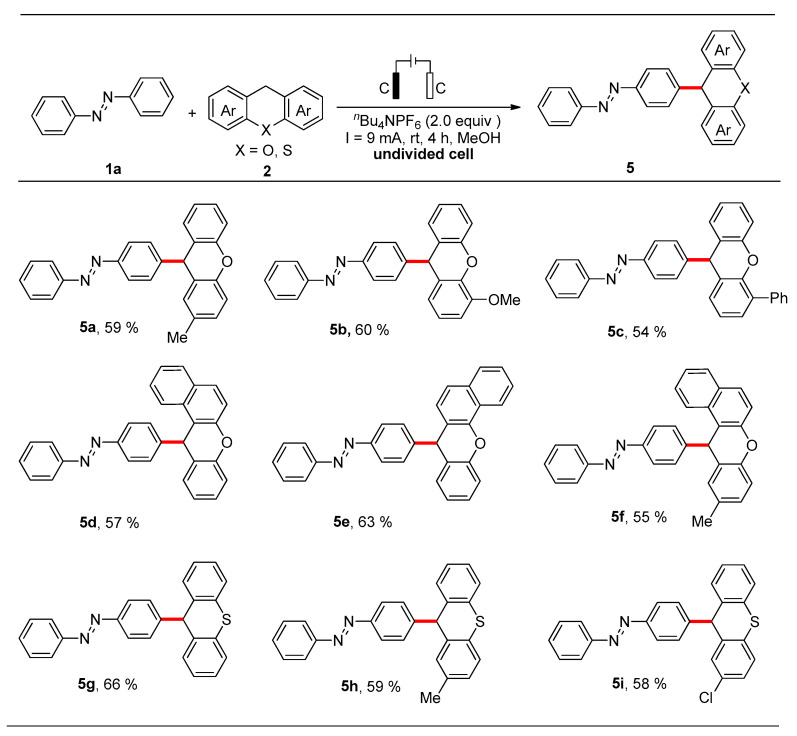
Scope of (thio)xanthenes *^a,b^*. *^a^* Reaction conditions: **1a** (0.30 mmol), **2** (0.36 mmol), *^n^*Bu_4_NPF_6_ (2.0 equiv), MeOH (5.0 mL), carbon rod anode (Φ 6 mm), carbon rod cathode (Φ 6 mm), rt, 4 h (Q = 4.48 F mol^−1^). *^b^* Isolated yields are shown.

We then turned our attention to the tolerance of the reaction towards functional groups on the xanthenes and thioxanthenes, and the results are listed in Scheme 4. More specifically, methyl, methoxy, phenyl on the different position of xanthene were well tolerated (**5a**–**c**). Benzoxanthene and derivatives, such as 12*H*-benzo[*a*]xanthene, 7*H*-benzo[*c*]xanthene, 10-methyl-12*H*-benzo[*a*]xanthene, reacted well with azobenzene, generating the products **5d**–**5f** in acceptable yields. Furthermore, some simple thioxanthenes were also examined, and products **5g**–**5i** were achieved in 58–66% yields.

**Scheme 5:**
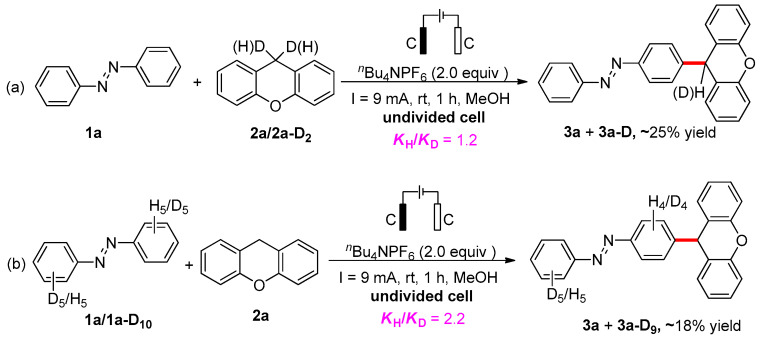
KIE experiments.

**Figure 1:**
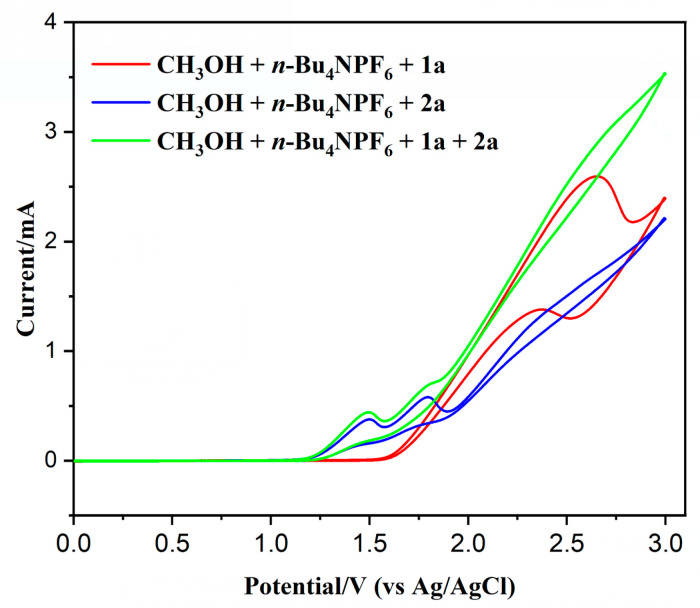
A cyclic voltammogram recorded in CH_3_OH with 0.1 M *^n^*Bu_4_NPF_6_ as the supporting electrolyte, **1a** (1 mM), **2a** (1 mM), **1a** + **2a** (1 mM).

Then, the KIE experiments were carried out to gain insight into the reaction mechanism (Scheme 5). The competing reaction of xanthene **2a** and deuterated xanthene **2a-D_2_** (1:1) with azobenzene determined the KIE with *K*_H_/*K*_D_ as 1.2, indicating that the cleavage of benzylic C(*sp*^3^)–H of xanthene was not the rate-determining step. In contrast, an obvious isotope effect (KIE = 2.2) was observed when performing the competing reaction of azobenzene **1a** and deuterated azobenzene **1a-D_10_** (1:1) with xanthene under standard conditions. These results showed that the cleavage of C_Ar_-H within azobenzene was presumably involved in the rate-determining step (For details, see Supplementary Materials). In addition, some cyclic voltammetry (CV) experiments were carried out to study the redox potential of the substrates (Figure 1). Remarkably, the oxidation potential of azobenzene **1a** (*E*_p_ = 2.2 V) was far higher than that of xanthene **2a** (*E*_p_ = 1.3, 1.7 V), demonstrating that the xanthene **2a** should be preferentially oxidized in the electrochemical system.

**Scheme 6:**
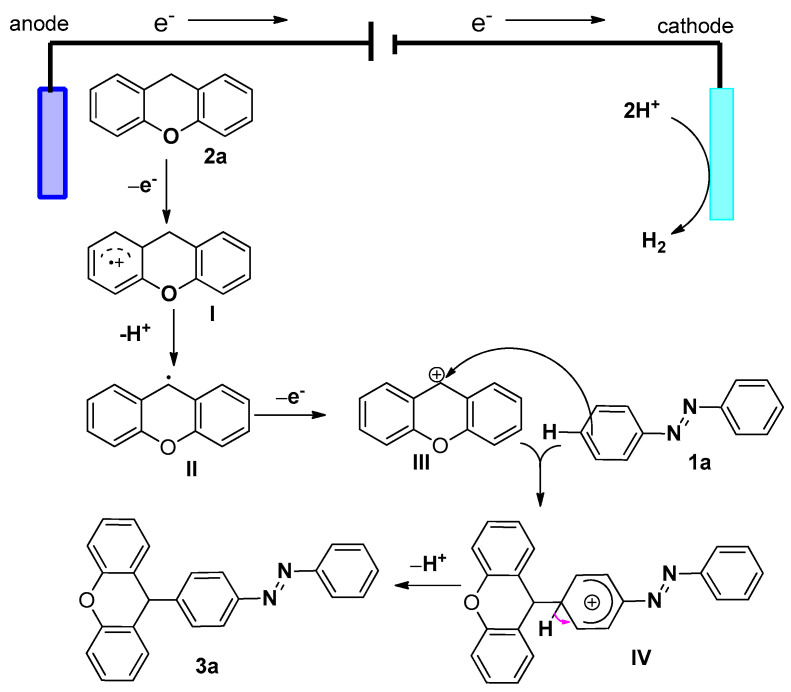
Possible reaction mechanism.

Based on the above mechanistic experiments and previous reports [41,42,43,45,46,47,48,49], a possible reaction mechanism is proposed in Scheme 6. Firstly, anodic oxidation of xanthene **2a** led to the formation of intermediate **I**, which was further deprotonated to generate radical **II**, followed by an anode oxidation to form the cationic species **III**. Secondly, a possible Friedel-Crafts reaction of **1a** with the cationic species **III** occurred to yield the intermediate **IV**. Finally, deprotonation of **IV** gave the product **3a**.

## 3. Materials and Methods

### 3.1. General Considerations

All ^1^H NMR and ^13^C NMR spectra were recorded on a 600 MHz Bruker FT-NMR spectrometer (600 MHz and 151 MHz, respectively). All chemical shifts are given as δ value (ppm) with reference to tetramethylsilane (TMS) as an internal standard. The peak patterns are indicated as follows: s, singlet; d, doublet; t, triplet; m, multiplet; q, quartet. The coupling constants, *J*, are reported in Hertz (Hz). High resolution mass spectroscopy data of the products were collected on an Agilent Technologies 6540 UHD Accurate-Mass Q-TOF LC/MS (ESI) and a Thermo Fisher Scientific LTQ FTICR-MS instrument. Melting points were determined in open capillary tube using WRS-1B digital melting point apparatus.

The starting materials, such as azobenzenes and xanthenes, were prepared according to the reported methods [42,43,50,51]. All the solvents are commercially available and directly used in this electrochemical system. Products were purified by flash chromatography on silica gels, eluting with petroleum ether/ethyl acetate (100:1 to 20:1).

### 3.2. Typical Procedure for the Synthesis of ***3a***

Azobenzene (**1a**, 0.30 mmol, 1.0 equiv), xanthene (**2a**, 0.36 mmol, 1.2 equiv), *^n^*Bu_4_NPF_6_ (0.60 mmol, 2.0 equiv) and CH_3_OH (5.0 mL) were sequentially added into a 15.0 mL oven-dried undivided single necked bottle that equipped with a magnetic stirrer bar and sealed with rubber plugs under air atmosphere. A carbon rod (Φ 6 mm) anode and a carbon rod (Φ 6 mm) were used as the cathode in the bottle. About 1.0 cm of the carbon rod was under the solution. The reaction mixture was stirred and electrolyzed at a constant current of 9 mA under air at room temperature for 4 h. After completion of the reaction, the solution was concentrated in vacuum. The resulting crude mixture was purified by flash column chromatography (petroleum ether/ethyl acetate = 100:1) to give the desired product **3a** as an orange solid (82.6 mg, 76% yield).

*(E)-1-(4-(9H-Xanthen-9-yl)phenyl)-2-phenyldiazene (***3a***)*: Prepared following general procedure and the reaction mixture was purified by flash column chromatography with petroleum ether and ethyl acetate (PE/EA = 100:1) to afford the product **3a** (82.6 mg, 76% yield). Orange solid; m.p.: 179.6~181.5 °C. ^1^H NMR (600 MHz, CDCl_3_) δ 7.87 (d, *J* = 7.2 Hz, 2H), 7.83 (d, *J* = 8.4 Hz, 2H), 7.50 (t, *J* = 7.2 Hz, 2H), 7.46 (t, *J* = 7.2 Hz, 1H), 7.35 (d, *J* = 8.4 Hz, 2H), 7.23 (t, *J* = 7.8 Hz, 2H), 7.16 (d, *J* = 7.8 Hz, 2H), 7.08 (d, *J* = 7.2 Hz, 2H), 7.00 (t, *J* = 7.8 Hz, 2H), 5.36 (s, 1H). ^13^C NMR (151 MHz, CDCl_3_) δ 152.7, 151.5, 151.0, 149.4, 130.9, 129.7, 129.2, 129.1, 128.2, 123.8, 123.3, 122.8, 116.7, 44.3. HRMS (ESI) calcd for C_25_H_19_N_2_O^+^ [M + H]^+^ 363.1492, found 363.1496.

*(E)-1-(4-(9H-Xanthen-9-yl)phenyl)-2-(p-tolyl)diazene (***3b***)*: Prepared following general procedure and the reaction mixture was purified by flash column chromatography with petroleum ether and ethyl acetate (PE/EA = 100:1) to afford the product **3b** (87.1 mg, 77% yield). Orange solid; m.p.: 176.4~178.2 °C. ^1^H NMR (600 MHz, CDCl_3_) δ 7.82 (d, *J* = 7.8 Hz, 2H), 7.80 (d, *J* = 8.4 Hz, 2H), 7.35 (d, *J* = 8.4 Hz, 2H), 7.30 (d, *J* = 7.8 Hz, 2H), 7.24 (t, *J* = 7.8 Hz, 2H), 7.17 (d, *J* = 8.4 Hz, 2H), 7.08 (d, *J* = 7.8 Hz, 2H), 7.00 (t, *J* = 7.8 Hz, 2H), 5.35 (s, 1H), 2.43 (s, 3H). ^13^C NMR (151 MHz, CDCl_3_) δ 151.5, 151.0, 150.8, 149.0, 141.5, 129.7, 129.1, 128.1, 123.8, 123.3, 123.2, 122.8, 116.6, 44.2, 21.5. HRMS (ESI) calcd for C_26_H_21_N_2_O^+^ [M + H]^+^ 377.1648, found 377.1650.

*(E)-1-(4-(9H-Xanthen-9-yl)phenyl)-2-(4-ethylphenyl)diazene (***3c***)*: Prepared following general procedure and the reaction mixture was purified by flash column chromatography with petroleum ether and ethyl acetate (PE/EA = 100:1) to afford the product **3c** (85.3 mg, 73% yield). Orange solid; m.p.: 178.6~181.1 °C. ^1^H NMR (600 MHz, CDCl_3_) δ 7.82 (d, *J* = 7.8 Hz, 4H), 7.35 (d, *J* = 8.4 Hz, 2H), 7.33 (d, *J* = 8.4 Hz, 2H), 7.23 (t, *J* = 7.2 Hz, 2H), 7.16 (d, *J* = 8.4 Hz, 2H), 7.08 (d, *J* = 7.8 Hz, 2H), 7.00 (t, *J* = 7.8 Hz, 2H), 5.35 (s, 1H), 2.73 (q, *J* = 7.8 Hz, 2H), 1.28 (t, *J* = 7.8 Hz, 3H). ^13^C NMR (151 MHz, CDCl_3_) δ 151.5, 151.0, 149.0, 147.7, 129.7, 129.1, 128.5, 128.1, 123.8, 123.3, 123.2, 122.9, 118.0, 116.7, 44.2, 28.8, 15.4. HRMS (ESI) calcd for C_27_H_23_N_2_O^+^ [M + H]^+^ 391.1805, found 391.1805.

*(E)-1-(4-(9H-Xanthen-9-yl)phenyl)-2-(4-isopropylphenyl)diazene (***3d***)*: Prepared following general procedure and the reaction mixture was purified by flash column chromatography with petroleum ether and ethyl acetate (PE/EA = 100:1) to afford the product **3d** (90.8 mg, 75% yield). Red solid; m.p.: 177.6~179.3 °C. ^1^H NMR (600 MHz, CDCl_3_) δ 7.83–7.81 (m, 4H), 7.36–7.34 (m, 4H), 7.23 (t, *J* = 7.2 Hz, 2H), 7.16 (d, *J* = 7.8 Hz, 2H),7.08 (d, *J* = 7.2 Hz, 2H), 7.00 (t, *J* = 7.8 Hz, 2H), 5.35 (s, 1H), 3.01–2.96 (m, 1H), 1.30 (d, *J* = 6.6 Hz, 6H). ^13^C NMR (151 MHz, CDCl_3_) δ 152.3, 151.5, 151.1, 151.0, 149.0, 129.7, 129.1, 128.1, 127.1, 123.8, 123.3, 123.2, 122.9, 116.7, 44.2, 34.1, 23.8. HRMS (ESI) calcd for C_28_H_24_N_2_O^+^ [M + H]^+^ 405.1961, found 405.1964.

*(E)-1-(4-(9H-Xanthen-9-yl)phenyl)-2-(4-(tert-butyl)phenyl)diazene (***3e***)*: Prepared following general procedure and the reaction mixture was purified by flash column chromatography with petroleum ether and ethylacetate (PE/EA = 100:1) to afford the product **3e** (89.1 mg, 71% yield). Red solid; m.p.: 179.4~180.6 °C. ^1^H NMR (600 MHz, CDCl_3_) δ 7.82 –7.81(m, 4H), 7.52 (d, *J* = 8.4 Hz, 2H), 7.35 (d, *J* = 7.8 Hz, 2H), 7.23(t, *J* = 7.2 Hz, 2H), 7.16 (d, *J* = 7.8 Hz, 2H), 7.00 (d, *J* = 7.8 Hz, 2H), 6.91 (t, *J* = 7.2 Hz, 2H), 5.35 (s, 1H), 1.37 (s, 9H). ^13^C NMR (151 MHz, CDCl_3_) δ 154.5, 151.6, 151.0, 150.6, 149.0, 129.7, 129.1, 128.1, 126.0, 123.8, 123.3, 123.2, 122.5, 116.7, 44.2, 35.0, 31.2. HRMS (ESI) calcd for C_29_H_21_N_2_O^+^ [M + H]^+^ 419.2118, found 419.2119.

*(E)-1-(4-(9H-Xanthen-9-yl)phenyl)-2-(4-(trifluoromethoxy)phenyl)diazene (***3f***)*: Prepared following general procedure and the reaction mixture was purified by flash column chromatography with petroleum ether and ethyl acetate (PE/EA = 100:1) to afford the product **3f** (84.3 mg, 63% yield). Orange solid; m.p.: 173.6~175.4 °C. ^1^H NMR (600 MHz, CDCl_3_) δ 7.91 (d, *J* = 9.0 Hz, 2H), 7.83 (d, *J* = 9.0 Hz, 2H), 7.36 (d, *J* = 7.8 Hz, 2H), 7.33 (d, *J* = 8.4 Hz, 2H), 7.24 (d, *J* = 7.2 Hz, 2H), 7.16 (d, *J* = 8.4 Hz, 2H), 7.08 (d, *J* = 7.8 Hz, 2H), 7.00 (t, *J* = 7.8 Hz, 2H), 5.36 (s, 1H). ^13^C NMR (151 MHz, CDCl_3_) δ 151.2, 151.0, 150.8, 149.8, 129.7, 129.2, 128.2, 126.2 (q, *J* = 268.3 Hz),124.3, 123.7, 123.4, 123.3, 122.8 (q, *J* = 47.3 Hz), 121.3, 120.6 (q, *J* = 147.2 Hz), 120.1, 116.7, 44.3. ^19^F NMR (565 MHz, CDCl_3_) δ -57.70. HRMS (ESI) calcd for C_26_H_18_F_3_N_2_O_2_^+^ [M + H]^+^ 447.1315, found 447.1319

*(E)-1-(4-((4-(9H-xanthen-9-yl)phenyl)diazenyl)phenyl)ethan-1-one (***3g***)*: Prepared following general procedure and the reaction mixture was purified by flash column chromatography with petroleum ether and ethylacetate (PE/EA = 20:1) to afford the product 3g (72.6 mg, 60% yield). Orange solid; m.p.: 176.6~178.5 °C. ^1^H NMR (600 MHz, CDCl_3_) δ 8.09 (d, *J* = 8.4 Hz, 2H), 7.92 (d, *J* = 8.4 Hz, 2H), 7.86 (d, *J* = 8.4 Hz, 2H), 7.37 (d, *J* = 7.8 Hz, 2H), 7.24 (t, *J* = 7.2 Hz, 2H), 7.16 (d, *J* = 7.8 Hz, 2H), 7.08 (d, *J* = 7.2 Hz, 2H), 7.00 (t, *J* = 6.6 Hz, 2H), 5.37 (s, 1H), 2.65 (s, 3H). ^13^C NMR (151 MHz, CDCl_3_) δ 197.4, 155.1, 151.3, 151.0, 150.2, 138.3, 129.6, 129.3, 129.2, 128.2, 123.7, 123.6, 123.4, 122.8, 116.7, 44.3, 26.8. HRMS (ESI) calcd for C_27_H_21_N_2_O_2_^+^ [M + H]^+^ 405.1598, found 405.1595.

*(E)-4-((4-(9H-Xanthen-9-yl)phenyl)diazenyl)benzonitrile (***3h***)*: Prepared following general procedure and the reaction mixture was purified by flash column chromatography with petroleum ether and ethyl acetate (PE/EA = 100:1) to afford the product **3h** (66.1 mg, 57% yield). Red solid; m.p.: 175.3~177.0 °C. ^1^H NMR (600 MHz, CDCl_3_) δ 7.93 (d, *J* = 9 Hz, 2H), 7.86 (d, *J* = 8.4 Hz, 2H), 7.79 (d, *J* = 8.4 Hz, 2H), 7.38 (d, *J* = 8.4 Hz, 2H), 7.25 (d, *J* = 6.6 Hz, 2H), 7.17 (d, *J* = 8.4 Hz, 2H), 7.08 (d, *J* = 7.2 Hz, 2H), 7.01 (t, *J* = 7.2 Hz, 2H), 5.37 (s, 1H). ^13^C NMR (151 MHz, CDCl_3_) δ 154.5, 151.1, 151.0, 150.7, 133.2, 129.6, 129.2, 128.3, 123.8, 123.5, 123.4, 123.3, 118.5, 116.8, 113.8, 44.3. HRMS (ESI) calcd for C_26_H_18_N_3_O^+^ [M + H]^+^ 388.1444, found 388.1444.

*(E)-1-(4-(9H-Xanthen-9-yl)phenyl)-2-(4-(trifluoromethyl)phenyl)diazene (***3i***)*: Prepared following general procedure and the reaction mixture was purified by flash column chromatography with petroleum ether and ethyl acetate (PE/EA = 100:1) to afford the product **3i** (89.2 mg, 69% yield). Orange solid; m.p.: 175.5~176.9 °C. ^1^H NMR (600 MHz, CDCl_3_) δ 7.94 (d, *J* = 8.4 Hz, 2H), 7.85 (d, *J* = 7.8 Hz, 2H), 7.74 (d, *J* = 8.4 Hz, 2H), 7.36 (d, *J* = 8.4 Hz, 2H), 7.23 (t, *J* = 5.4 Hz, 2H), 7.16 (d, *J* = 8.4 Hz, 2H), 7.07 (d, *J* = 7.8 Hz, 2H), 6.99 (t, *J* = 7.2 Hz, 2H), 5.35 (s, 1H). ^13^C NMR (151 MHz, CDCl_3_) δ 154.4, 151.2, 151.0, 150.3, 132.1 (q, *J* = 31.9 Hz), 129.6, 129.2, 128.2, 126.2 (q, *J* = 3.9 Hz), 124.8 (q, *J* = 272.4 Hz), 123.7, 123.6, 123.4, 122.9, 116.7, 44.3. ^19^F NMR (565 MHz, CDCl_3_) δ -62.5. HRMS (ESI) calcd for C_26_H_18_F_3_N_2_O^+^ [M + H] ^+^ 431.1336, found 431.1335.

*(E)-1-(4-(9H-Xanthen-9-yl)phenyl)-2-(m-tolyl)diazene (***3k***); (*E*)-1-(3-Methyl-4-(9H-xanthen-9-yl)phenyl)-2-phenyldiazene (***3k’***)*: Prepared following general procedure and the reaction mixture was purified by flash column chromatography with petroleum ether and ethylacetate (PE/EA = 100:1) to afford the product **3k** and **3k’** (81.2 mg, 72% yield). Red solid; m.p.: 176.6~178.9 °C. ^1^H NMR (600 MHz, CDCl_3_) δ 7.89 (d, *J* = 7.2 Hz, 1.6H), 7.82 (d, *J* = 8.4 Hz, 2H), 7.74 (s, 0.8H), 7.72 (d, *J* = 7.8 Hz, 0.8H), 7.68 (d, *J* = 5.4 Hz, 1.6H), 7.52 (t, *J* = 7.2 Hz, 1.8H), 7.47 (t, *J* = 7.2 Hz, 0.8H), 7.39 (t, *J* = 7.8 Hz, 1H), 7.35 (d, *J* = 8.4 Hz, 2H), 7.33 (d, *J* = 7.8 Hz, 0.8H), 7.28 (d, *J* = 8.4 Hz, 1H), 7.23 (q, *J* = 8.4 Hz, 4H), 7.16 (d, *J* = 7.8 Hz, 2H), 7.12 (d, *J* = 7.8 Hz, 1.6H), 7.08 (d, *J* = 7.8 Hz, 2H), 7.00 (t, *J* = 7.8 Hz, 2H), 6.95 (t, *J* = 7.8 Hz, 1.6H), 6.89 (d, *J* = 7.8 Hz, 1.6H), 5.65 (s, 0.8H), 5.35 (s, 1H), 2.45 (s, 3H), 2.33 (s, 2.4H). ^13^C NMR (151 MHz, CDCl_3_) δ 152.8, 151.5, 151.5, 151.0, 150.9, 149.3, 146.6, 139.0, 137.1, 131.9, 131.7, 130.9, 129.7, 129.2, 129.2, 129.1, 128.9, 128.2, 128.1, 125.4, 123.8, 123.5, 123.3, 123.3, 123.2, 122.9, 122.8, 121.1, 120.4, 116.7, 116.5, 44.3, 41.2, 21.4, 20.2. HRMS (ESI) calcd for C_26_H_21_N_2_O^+^ [M + H]^+^ 377.1648, found 377.1648.

*(E)-1-(4-(9H-Xanthen-9-yl)phenyl)-2-(2,3-dimethylphenyl)diazene (***3l***)*: Prepared following general procedure and the reaction mixture was purified by flash column chromatography with petroleum ether and ethyl acetate (PE/EA = 100:1) to afford the product **3l** (92.3 mg, 79% yield). Red solid; m.p.: 178.6~180.2 °C. ^1^H NMR (600 MHz, CDCl_3_) δ 7.84 (d, *J* = 8.4 Hz, 2H), 7.44 (d, *J* = 8.4 Hz, 1H), 7.35 (d, *J* = 8.4 Hz, 2H), 7.24 (d, *J* = 6.6 Hz, 3H), 7.16 (d, *J* = 8.4 Hz, 3H), 7.10 (d, *J* = 7.8 Hz, 2H), 7.01 (t, *J* = 6.6 Hz, 2H), 5.36 (s, 1H), 2.61 (s, 3H), 2.37 (s, 3H). ^13^C NMR (151 MHz, CDCl_3_) δ 151.8, 151.0, 151.0, 149.1, 138.2, 136.8, 132.1, 129.7, 129.0, 128.1, 125.7, 123.9, 123.4, 123.3, 116.7, 113.1, 44.2, 19.9, 13.2. HRMS (ESI) calcd for C_27_H_23_N_2_O^+^ [M + H]^+^ 391.1805, found 391.1806.

*(E)-1-(4-(9H-Xanthen-9-yl)phenyl)-2-(2,4-dimethy phenyl)diazene (***3m***)*: Prepared following general procedure and the reaction mixture was purified by flash column chromatography with petroleum ether and ethyl acetate (PE/EA = 100:1) to afford the product **3m** (87.5 mg, 75% yield). Red solid; m.p.: 178.6~180.3 °C. ^1^H NMR (600 MHz, CDCl_3_) δ 7.82 (d, *J* = 8.4 Hz, 2H), 7.54 (d, *J* = 8.4 Hz, 1H), 7.35 (d, *J* = 8.4 Hz, 2H), 7.24 (t, *J* = 7.2 Hz, 2H), 7.17 (d, *J* = 8.4 Hz, 2H), 7.14 (s, 1H), 7.09 (d, *J* = 7.2 Hz, 2H), 7.06 (d, *J* = 7.8 Hz, 1H), 7.01 (t, *J* = 7.8 Hz, 2H), 5.35 (s, 1H), 2.66 (s, 3H), 2.38 (s, 3H). ^13^C NMR (151 MHz, CDCl_3_) δ 151.8, 151.0, 148.8, 148.8, 141.3, 138.2, 131.8, 129.7, 129.0, 128.1, 128.1, 127.3, 123.9, 123.3, 116.7, 115.2, 44.2, 21.4, 17.4. HRMS (ESI) calcd for C_27_H_23_N_2_O^+^ [M + H]^+^ 391.1805, found 391.1809.

*(E)-1-(4-(9H-xanthen-9-yl)phenyl)-2-(3,4-dimethylphenyl)diazene (***3n***)*: Prepared following general procedure and the reaction mixture was purified by flash column chromatography with petroleum ether and ethyl acetate (PE/EA = 100:1) to afford the product **3n** (83.1 mg, 71% yield). Red solid; m.p.: 180.1~181.5 °C. ^1^H NMR (600 MHz, CDCl_3_) δ 7.81 (d, *J* = 8.4 Hz, 2H), 7.67 (s, 1H), 7.64 (d, *J* = 8.4 Hz, 1H), 7.35 (d, *J* = 8.4 Hz, 2H), 7.26 (d, *J* = 8.4 Hz, 1H), 7.24 (t, *J* = 8.4 Hz, 2H), 7.16 (d, *J* = 7.8 Hz, 2H), 7.08 (d, *J* = 7.2 Hz, 2H), 7.00 (t, *J* = 7.2 Hz, 2H), 5.35 (s, 1H), 2.35 (s, 3H), 2.33 (s, 3H). ^13^C NMR (151 MHz, CDCl_3_) δ 151.6, 151.3, 151.0, 149.0, 140.3, 137.4, 130.3, 129.7, 129.2, 128.1, 123.9, 123.5, 123.3, 123.2, 120.8, 116.7, 44.2, 19.9. HRMS (ESI) calcd for C_27_H_23_N_2_O^+^ [M + H]^+^ 391.1805, found 391.1807.

*(E)-1-(4-(9H-xanthen-9-yl)phenyl)-2-(3,5-dimethylphenyl)diazene (***3o***)*: Prepared following general procedure and the reaction mixture was purified by flash column chromatography with petroleum ether and ethyl acetate (PE/EA = 100:1) to afford the product **3o** (85.3 mg, 73% yield). Red solid; m.p.: 179.9~181.7 °C. ^1^H NMR (600 MHz, CDCl_3_) δ 7.83 (d, *J* = 8.4 Hz, 2H), 7.51 (s, 2H), 7.36 (d, *J* = 8.4 Hz, 2H), 7.24 (t, *J* = 7.2 Hz, 2H), 7.17 (d, *J* = 8.4 Hz, 2H), 7.12 (s, 1H), 7.09 (d, *J* = 7.2 Hz, 2H), 7.01 (t, *J* = 7.8 Hz, 2H), 5.36 (s, 1H), 2.42 (s, 6H). ^13^C NMR (151 MHz, CDCl_3_) δ 152.9, 151.5, 151.0, 149.2, 138.8, 132.7, 129.7, 129.2, 128.2, 123.9, 123.4, 123.3, 120.6, 116.7, 44.3, 21.3. HRMS (ESI) calcd for C_27_H_23_N_2_O^+^ [M + H]^+^ 391.1805, found 391.1810.

*(E)-1-(3-Methyl-4-(9H-xanthen-9-yl)phenyl)-2-(m-tolyl)diazene (***3p***)*: Prepared following general procedure and the reaction mixture was purified by flash column chromatography with petroleum ether and ethyl acetate (PE/EA = 100:1) to afford the product **3p** (60.7 mg, 52% yield). Orange solid; m.p.: 175.6~177.5 °C. ^1^H NMR (600 MHz, CDCl_3_) δ 7.73–7.70 (m, 4H), 7.40 (t, *J* = 8.4 Hz, 1H), 7.32 (d, *J* = 7.8 Hz, 1H), 7.29 (d, *J* = 7.2 Hz, 1H), 7.22 (t, *J* = 8.4 Hz, 2H), 7.12 (d, *J* = 7.2 Hz, 2H), 6.95 (t, *J* = 7.8 Hz, 2H), 6.89 (d, *J* = 7.8 Hz, 2H), 5.64 (s, 1H), 2.46 (s, 3H), 2.33 (s, 3H). ^13^C NMR (151 MHz, CDCl_3_) δ 152.8, 151.5, 150.8, 1465, 139.0, 137.0, 131.9, 131.7, 129.2, 128.9, 128.0, 125.3, 123.5, 123.2, 122.8, 121.0, 120.4, 116.4, 41.2, 21.4, 20.1. HRMS (ESI) calcd for C_27_H_23_N_2_O^+^ [M + H]^+^ 391.1805, found 391.1805.

*(E)-1-(2-Isopropyl-4-(9H-xanthen-9-yl)phenyl)-2-(2-isopropylphenyl)diazene (***3q***)*: Prepared following general procedure and the reaction mixture was purified by flash column chromatography with petroleum ether and ethyl acetate (PE/EA = 100:1) to afford the product **3q** (85.6 mg, 64% yield). Red solid; m.p.: 177.9~179.1 °C. ^1^H NMR (600 MHz, CDCl_3_) δ 7.47 (d, *J* = 7.8 Hz, 1H), 7.42 (d, *J* = 7.8 Hz, 1H), 7.35–7.31 (m, 2H), 7.28 (s, 1H), 7.17–7.16 (m, 1H), 7.13 (t, *J* = 6.6 Hz, 2H), 7.06 (d, *J* = 8.4 Hz, 2H), 7.01 (d, *J* = 7.8 Hz, 2H), 6.93–6.90 (m, 3H), 5.24 (s, 1H), 4.07–4.02 (m, 1H), 4.02–3.96 (m, 1H), 1.26 (d, *J* = 7.2 Hz, 6H), 1.22 (d, *J* = 6.6 Hz, 6H). ^13^C NMR (151 MHz, CDCl_3_) δ 151.1, 150.0, 149.1, 148.7, 148.3, 147.1, 130.9, 129.6, 128.0, 126.5, 126.3, 126.2, 126.1, 124.0, 123.3, 116.6, 116.3, 115.4, 44.5, 27.9, 27.7, 23.8, 23.8. HRMS (ESI) calcd for C_31_H_31_N_2_O^+^ [M + H]^+^ 447.2431, found 447.2428.

*(E)-1-(4-(9H-xanthen-9-yl)phenyl)-2-(3-methyl-4-(9H-xanthen-9-yl)phenyl)diazene (***4a***)*: Prepared following general procedure and the reaction mixture was purified by flash column chromatography with petroleum ether and ethyl acetate (PE/EA = 100:1) to afford the product **4a** (113.4 mg, 68% yield). Orange solid; m.p.: 238.4~240.2 °C. ^1^H NMR (600 MHz, CDCl_3_) δ 7.80 (d, *J* = 9.0 Hz, 2H), 7.68 (s, 1H), 7.67 (d, *J* = 8.4 Hz, 1H), 7.34 (d, *J* = 8.4 Hz, 2H), 7.30 (d, *J* = 7.8 Hz, 1H), 7.25–7.20 (m, 4H), 7.16 (d, *J* = 7.8 Hz, 2H), 7.12 (d, *J* = 8.4 Hz, 2H), 7.08 (d, *J* = 7.2 Hz, 2H), 7.00 (t, *J* = 7.8 Hz, 2H), 6.93 (t, *J* = 7.8 Hz, 2H), 6.88 (d, *J* = 7.2 Hz, 2H), 5.63 (s, 1H), 5.35 (s, 1H), 2.31 (s, 3H). ^13^C NMR (151 MHz, CDCl_3_) δ 151.5, 151.0, 150.8, 149.3, 146.5, 137.0, 131.9, 129.7, 129.2, 129.1, 128.1, 128.0, 125.3, 123.8, 123.4, 123.3, 123.2, 123.1, 120.9, 116.7, 116.4, 44.2, 41.1, 20.1. HRMS (ESI) calcd for C_39_H_29_N_2_O_2_^+^ [M + H]^+^ 557.2224, found 557.2224.

*(E)-1-(4-(9H-xanthen-9-yl)phenyl)-2-(2-isopropyl-4-(9H-xanthen-9-yl)phenyl)diazene (***4b***)*: Prepared following general procedure and the reaction mixture was purified by flash column chromatography with petroleum ether and ethyl acetate (PE/EA = 100:1) to afford the product **4b** (110.3 mg, 63% yield). Red solid; m.p.: 237.4~238.9 °C. ^1^H NMR (600 MHz, CDCl_3_) δ 7.79 (d, *J* = 8.4 Hz, 2H), 7.45 (d, *J* = 8.4 Hz, 1H), 7.33 (d, *J* = 8.4 Hz, 2H), 7.31 (s, 1H), 7.22 (q, *J* = 6.6 Hz, 4H), 7.15 (t, *J* = 7.2 Hz, 4H), 7.09 (d, *J* = 7.8 Hz, 2H), 7.06 (d, *J* = 7.2 Hz, 2H), 6.99 (q, *J* = 7.8 Hz, 5H), 5.34 (s, 1H), 5.31 (s, 1H), 4.01–3.94 (m, 1H), 1.28 (d, *J* = 7.2 Hz, 6H). ^13^C NMR (151 MHz, CDCl_3_) δ 151.7, 151.0, 151.0, 149.2, 149.1, 148.5, 148.1, 129.6, 129.6, 129.0, 128.1, 128.0, 126.6, 126.3, 124.0, 123.8, 123.4, 123.3, 123.2, 116.7, 116.6, 115.9, 44.4, 44.3, 28.0, 23.8. HRMS (ESI) calcd for C_41_H_33_N_2_O_2_^+^ [M + H]^+^ 585.2537, found 585.2538.

*(E)-1,2-Bis(2-isopropyl-4-(9H-xanthen-9-yl)phenyl)diazene (***4c***)*: Prepared following general procedure and the reaction mixture was purified by flash column chromatography with petroleum ether and ethyl acetate (PE/EA = 100:1) to afford the product **4c** (107.1 mg, 57% yield). White solid; m.p.: 242.4~246.1 °C. ^1^H NMR (600 MHz, CDCl_3_) δ 7.46 (d, *J* = 8.4 Hz, 2H), 7.34 (s, 2H), 7.22 (t, *J* = 7.8 Hz, 4H), 7.15 (d, *J* = 7.8 Hz, 4H), 7.09 (d, *J* = 7.2 Hz, 4H), 7.00 (t, *J* = 7.8 Hz, 6H), 5.31 (s, 2H), 4.08–4.01 (m, 2H), 1.29 (d, *J* = 7.2 Hz, 12H). ^13^C NMR (151 MHz, CDCl_3_) δ 151.1, 149.0, 148.6, 148.2, 129.6, 128.0, 126.4, 126.1, 124.0, 123.3, 44.5, 27.9, 23.8. HRMS (ESI) calcd for C_44_H_39_N_2_O_2_^+^ [M + H]^+^ 627.3006, found 627.3004.

*(E)-1-(4-(2-Methyl-9H-xanthen-9-yl)phenyl)-2-phenyldiazene (***5a***)*: Prepared following general procedure and the reaction mixture was purified by flash column chromatography with petroleum ether and ethyl acetate (PE/EA = 100:1) to afford the product **5a** (66.4 mg, 59% yield). Orange solid; m.p.: 178.6~179.9 °C. ^1^H NMR (600 MHz, CDCl_3_) δ 7.87 (d, *J* = 7.2 Hz, 2H), 7.83 (d, *J* = 8.4 Hz, 2H), 7.50 (t, *J* = 7.2 Hz, 2H), 7.46 (t, *J* = 7.2 Hz, 1H), 7.35 (d, *J* = 8.4 Hz, 2H), 7.22 (t, *J* = 7.2 Hz, 1H), 7.14 (d, *J* = 8.4 Hz, 1H), 7.07–7.02 (m, 3H), 6.98 (t, *J* = 7.2 Hz, 1H), 6.86 (s, 1H), 5.30 (s, 1H), 2.23 (s, 3H). ^13^C NMR (151 MHz, CDCl_3_) δ 152.7, 151.4, 151.1, 149.6, 148.9, 132.7, 130.9, 129.8, 129.7, 129.1, 129.0, 128.8, 128.1, 123.8, 123.3, 123.3, 123.1, 122.8, 116.6, 116.4, 44.3, 20.7. HRMS (ESI) calcd for C_26_H_21_N_2_O^+^ [M + H]^+^ 377.1648, found 377.1647.

*(E)-1-(4-(4-Methoxy-9H-xanthen-9-yl)phenyl)-2-phenyldiazene (***5b***)*: Prepared following general procedure and the reaction mixture was purified by flash column chromatography with petroleum ether and ethyl acetate (PE/EA = 20:1) to afford the product **5b** (70.3 mg, 60% yield). Orange solid; m.p.: 180.5~182.2 °C. ^1^H NMR (600 MHz, CDCl_3_) δ 7.87 (d, *J* = 7.2 Hz, 2H), 7.83 (d, *J* = 8.4 Hz, 2H), 7.50 (d, *J* = 7.2 Hz, 2H), 7.45 (t, *J* = 7.2 Hz, 1H), 7.36 (d, *J* = 8.4 Hz, 2H), 7.30 (d, *J* = 8.4 Hz, 1H), 7.24 (t, *J* = 8.4 Hz, 1H), 7.09 (d, *J* = 7.2 Hz, 1H), 7.01 (t, *J* = 6.6 Hz, 1H), 6.94 (t, *J* = 7.8 Hz, 1H), 6.84 (d, *J* = 7.8 Hz, 1H), 6.69 (d, *J* = 7.8, 1H), 5.35 (s, 1H), 3.98 (s, 3H). ^13^C NMR (151 MHz, CDCl_3_) δ 152.7, 151.4, 150.8, 149.4, 148.1, 140.7, 130.9, 129.6, 129.1, 129.0, 128.1, 124.7, 123.6, 123.6, 123.3, 122.9, 1228, 121.3, 117.1, 110.2, 56.2, 44.3. HRMS (ESI) calcd for C_26_H_21_N_2_O^+^ [M + H]^+^ 393.1598, found 393.1600.

*(E)-1-Phenyl-2-(4-(4-phenyl-9H-xanthen-9-yl)phenyl)diazene (***5c***)*: Prepared following general procedure and the reaction mixture was purified by flash column chromatography with petroleum ether and ethyl acetate (PE/EA = 100:1) to afford the product **5c** (70.7 mg, 54% yield). Orange solid; m.p.: 182.6~183.8 °C. ^1^H NMR (600 MHz, CDCl_3_) δ 7.88 (d, *J* = 7.2 Hz, 2H), 7.85 (d, *J* = 8.4 Hz, 2H), 7.68 (d, *J* = 7.2 Hz, 2H), 7.51 (t, *J* = 7.2 Hz, 4H), 7.47, (d, *J* = 7.2 Hz, 1H), 7.42, (d, *J* = 7.2 Hz, 1H),7.40 (d, *J* = 8.4 Hz, 2H), 7.30 (d, *J* = 7.2 Hz, 1H), 7.21 (t, *J* = 8.4 Hz, 1H), 7.13–7.07 (m, 4H), 7.02 (t, *J* = 7.2 Hz, 1H), 5.41 (s, 1H). ^13^C NMR (151 MHz, CDCl_3_) δ 152.7, 151.5, 151.1, 149.1, 148.0, 137.7, 130.9, 130.1, 129.7, 129.7, 129.6, 129.3, 129.0, 128.9, 128.1, 128.0, 127.2, 124.7, 124.1, 123.5, 123.3, 123.2, 122.8, 120.4, 116.8, 114.1, 44.8. HRMS (ESI) calcd for C_31_H_23_N_2_O^+^ [M + H]^+^ 439.1805, found 439.1803.

*(E)-1-(4-(12H-benzo[a]xanthen-12-yl)phenyl)-2-phenyldiazene (***5d***)*: Prepared following general procedure and the reaction mixture was purified by flash column chromatography with petroleum ether and ethyl acetate (PE/EA = 100:1) to afford the product **5d** (70.5 mg, 57% yield). Orange solid; m.p.: 179.7~181.4 °C. ^1^H NMR (600 MHz, CDCl_3_) δ 7.94 (d, *J* = 8.4 Hz, 1H), 7.84–7.82 (m, 4H), 7.76 (d, *J* = 8.4 Hz, 2H), 7.49–7.43 (m, 7H), 7.41 (d, *J* = 7.2 Hz, 1H), 7.37 (t, *J* = 7.2 Hz, 1H), 7.24–7.21 (m, 2H), 7.07 (t, *J* = 7.9 Hz, 1H), 5.92 (s, 1H). ^13^C NMR (151 MHz, CDCl_3_) δ 152.7, 151.3, 150.1, 149.4, 149.3, 131.6, 130.8, 130.8, 129.3, 129.3, 129.0, 128.6, 128.1, 127.9, 126.9, 124.2, 124.1, 123.7, 123.4, 123.0, 122.7, 118.0, 116.8, 115.2, 41.9. HRMS (ESI) calcd for C_29_H_21_N_2_O^+^ [M + H]^+^ 413.1648, found 413.1652.

*(E)-1-(4-(7H-benzo[c]xanthen-7-yl)phenyl)-2-phenyldiazene (***5e***)*: Prepared following general procedure and the reaction mixture was purified by flash column chromatography with petroleum ether and ethyl acetate (PE/EA = 100:1) to afford the product **5e** (77.6 mg, 63% yield). Orange solid; m.p.: 179.8~181.6 °C. ^1^H NMR (600 MHz, CDCl_3_) δ 7.94 (d, *J* = 8.4 Hz, 1H), 7.84–7.82 (m, 4H), 7.76 (d, *J* = 8.4 Hz, 2H), 7.49–7.43 (m, 7H), 7.40 (d, *J* = 7.8 Hz, 1H), 7.37 (t, *J* = 7.8 Hz, 1H), 7.25–7.21 (m, 2H), 7.07 (t, *J* = 7.2 Hz, 1H), 5.92 (s, 1H). ^13^C NMR (151 MHz, CDCl_3_) δ 152.7, 151.3, 150.1, 149.4, 149.3, 131.6, 130.8, 130.8, 129.3, 129.3, 129.0, 128.6, 128.1, 127.9, 126.9, 124.2, 124.1, 123.7, 123.4, 123.0, 122.7, 118.0, 116.8, 115.1, 41.9. HRMS (ESI) calcd for C_29_H_21_N_2_O^+^ [M + H]^+^ 413.1648, found 413.1653.

*(E)-1-(4-(10-Methyl-12H-benzo[a]xanthen-12-yl)phenyl)-2-phenyldiazene (***5f***)*: Prepared following general procedure and the reaction mixture was purified by flash column chromatography with petroleum ether and ethyl acetate (PE/EA = 100:1) to afford the product **5f** (70.2 mg, 55% yield). Orange solid; m.p.: 181.4~183.2 °C. ^1^H NMR (600 MHz, CDCl_3_) δ 7.93 (d, *J* = 8.4 Hz, 1H), 7.81 (d, *J* = 9.6 Hz, 4H), 7.75 (d, *J* = 9.0 Hz, 2H), 7.48–7.42 (m, 7H), 7.36 (t, *J* = 7.2 Hz, 1H), 7.18 (s, 1H), 7.10 (d, *J* = 8.4 Hz, 1H), 7.03 (d, *J* = 9.6 Hz, 1H), 5.86 (s, 1H), 2.29 (s, 3H). ^13^C NMR (151 MHz, CDCl_3_) δ 152.7, 149.5, 149.5, 148.0, 133.1, 131.7, 130.8, 130.8, 129.5, 129.3, 129.0, 128.7, 128.6, 128.2, 126.8, 124.1, 123.7, 123.4, 122.9, 122.7, 118.1, 116.5, 115.1, 41.9, 20.8. HRMS (ESI) calcd for C_30_H_23_N_2_O^+^ [M + H]^+^ 427.1805, found 427.1805.

*(E)-1-(4-(9H-Thioxanthen-9-yl)phenyl)-2-phenyldiazene (***5g***)*: Prepared following general procedure and the reaction mixture was purified by flash column chromatography with petroleum ether and ethyl acetate (PE/EA = 100:1) to afford the product **5g** (74.7 mg, 66% yield). White solid; m.p.: 176.1~177.9 °C. ^1^H NMR (600 MHz, CDCl_3_) δ 7.85 (d, *J* = 7.8 Hz, 2H), 7.74 (d, *J* = 8.4 Hz, 2H), 7.50–7.44 (m, 7H), 7.30 (d, *J* = 7.2 Hz, 2H), 7.27 (d, *J* = 8.4 Hz, 2H), 7.16 (d, *J* = 8.4 Hz, 2H), 5.41 (s, 1H). ^13^C NMR (151 MHz, CDCl_3_) δ 152.7, 151.3, 144.1, 136.9, 133.3, 130.8, 129.6, 129.0, 128.6, 127.3, 127.1, 126.7, 122.7, 122.7, 53.0. HRMS (ESI) calcd for C_25_H_19_N_2_S^+^ [M + H]^+^ 379.1263, found 379.1264.

*(E)-1-(4-(2-Methyl-9H-thioxanthen-9-yl)phenyl)-2-phenyldiazene (***5h***)*: Prepared following general procedure and the reaction mixture was purified by flash column chromatography with petroleum ether and ethylacetate (PE/EA = 100:1) to afford the product **5h** (69.4 mg, 59% yield). Orange solid; m.p.: 174.4~176.4 °C. ^1^H NMR (600 MHz, CDCl_3_) δ 7.85 (d, *J* = 7.2 Hz, 2H), 7.75 (d, *J* = 8.4 Hz, 2H), 7.49 (t, *J* = 7.2 Hz, 2H), 7.46–7.43 (m, 3H), 7.36 (d, *J* = 7.8 Hz, 1H), 7.30–7.27 (m, 3H), 7.18 (d, *J* = 8.4 Hz, 2H), 7.09 (d, *J* = 7.8 Hz, 1H), 5.36 (s, 1H), 2.39 (s, 3H). ^13^C NMR (151 MHz, CDCl_3_) δ 152.7, 151.3, 144.3, 137.1, 136.8, 136.6, 133.6, 130.8, 130.3, 129.8, 129.5, 129.0, 128.5, 127.9, 127.3, 127.1, 127.0, 126.6, 122.7, 122.7, 53.1, 21.1. HRMS (ESI) calcd for C_26_H_21_N_2_S^+^ [M + H]^+^ 393.1420, found 393.1419.

*(E)-1-(4-(2-Chloro-9H-thioxanthen-9-yl)phenyl)-2-phenyldiazene (***5i***)*: Prepared following general procedure and the reaction mixture was purified by flash column chromatography with petroleum ether and ethyl acetate (PE/EA = 100:1) to afford the product **5i** (71.7 mg, 58% yield). Orange solid; m.p.: 178.6~179.8 °C. ^1^H NMR (600 MHz, CDCl_3_) δ 7.86 (d, *J* = 7.2 Hz, 2H), 7.76 (d, *J* = 8.4 Hz, 2H), 7.49 (t, *J* = 7.2 Hz, 2H), 7.47–7.43 (m, 4H), 7.39 (d, *J* = 8.4 Hz, 1H), 7.31 (t, *J* = 7.8 Hz, 1H), 7.29 (d, *J* = 7.2 Hz, 1H), 7.25 (d, *J* = 8.4 Hz, 1H), 7.16 (d, *J* = 8.4 Hz, 2H), 5.35 (s, 1H). ^13^C NMR (151 MHz, CDCl_3_) δ 152.7, 151.4, 143.2, 138.7, 136.3, 132.9, 132.5, 131.9, 130.9, 129.6, 129.4, 129.0, 128.5, 128.4, 127.3, 127.2, 127.0, 122.8, 120.8, 120.3, 52.8. HRMS (ESI) calcd for C_25_H_18_ClN_2_S^+^ [M + H]^+^ 413.0874, found 413.0873.

## 4. Conclusions

In summary, we have established a mild protocol to access azobenzene derivatives through the electrochemical alkylation of simple azobenzenes with (thio)xanthenes. This electrochemical transformation proceeds well in the absence of any catalyst or external oxidant, and provides an atom-economic approach for the site-selective functionalization of azobenzenes. We postulate that this strategy can be extended to more challenging organic molecules akin to azobenzene for the development of sustainable electrochemical transformations.

## Figures and Tables

**Table 1:** Optimization of the reaction conditions ^*a*^.

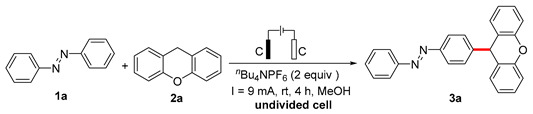		
Entry	Variation from the “Standard Conditions”	Yield (%) *^b^*
1	none	76
2	*^n^*Bu_4_NBF_4_ instead of *^n^*Bu_4_NPF_6_	62
3	*^n^*Bu_4_NI instead of *^n^*Bu_4_NPF_6_	35
4	LiClO_4_ instead of *^n^*Bu_4_NPF_6_	n.d.
5	Pt(+)|Pt(−) instead of C(+)|C(−)	n.d.
6	Pt(+)|C(−) instead of C(+)|C(−)	68
7	C(+)|Pt (−) instead of C(+)|C(−)	64
8	C(+)|GF(−) instead of C(+)|C(−)	19
9	GF(+)|C(−) instead of C(+)|C(−)	65
10	C(+)|Ni(−) instead of C(+)|C(−)	11
11	DCE instead of MeOH	55
12	CH_3_CN instead of MeOH	43
13	THF instead of MeOH	40
14	DMF instead of MeOH	29
15	Acetone instead of MeOH	n.d.
16	3 h instead of 4 h	55
17	5 h instead of 4 h	68
18	8 mA instead of 9 mA	46
19	10 mA instead of 9 mA	67
20	no electric current	n.d.
21	N_2_	74 *^c^*

*^a^* Reaction conditions: **1a** (0.30 mmol), **2a** (0.36 mmol), *^n^*Bu_4_NPF_6_ (1.5 equiv), MeOH (5.0 mL), carbon rod anode (Φ 6 mm), carbon rod cathode (Φ 6 mm), rt, 4 h (Q = 4.48 F mol^−1^). *^b^* Isolated yields. *^c^* N_2_. n.d. = not detected.

## Data Availability

Not applicable.

## Supplementary Materials

The following supporting information can be downloaded at: https://www.mdpi.com/article/10.3390/molecules27154967/s1, Figure S1: Experiment setup for electrochemical site-selective alkylation of azobenzenes with (thio)xanthenes; Figure S2: Experiment setup for the gram-scale synthesis of **3a**; Figure S3: X-ray structure of **3d** (ORTEP diagram with ellipsoid contour 50% probability); Gram-scale synthesis of **3a**; Mechanistic experiments; ^1^H, ^13^C, ^19^F NMR spectra of the products; Crystallographic data for **3d**; Determination of faradaic efficiency.

## Sample Availability

Samples of the compounds are available from the authors.
