# Supplementary material for: Electrochemical Site-Selective Alkylation of Azobenzenes with (Thio)Xanthenes
Source: Molecules. 2022 Aug 4;27(15):4967. doi: 10.3390/molecules27154967 (PMC9370205; doi:10.3390/molecules27154967)
Supplement: Supplementary file 1 [file molecules-27-04967-s001.zip › molecules-1844086-supplementary.pdf]

Supporting Information

Electrochemical Site-Selective Alkylation of Azobenzenes with (Thio)Xanthenes

Qiang Zhong,[a] Hui Gao,[a,b] Pei-Long Wang*,[a,c] Chao Zhou,[a] Tao Miao[a] and Hongji Li*,[a]

a Key Laboratory of Green and Precise Synthetic Chemistry and Applications, Ministry of Education, School of Chemistry and Materials Science, Huaibei Normal University, Huaibei, Anhui 235000, P. R. China.

b Key Laboratory for Chemistry and Molecular Engineering of Medicinal Resources, School of Chemistry and Pharmaceutical Sciences, Guangxi Normal University, Guilin 541004, China.

c Information College, Huaibei Normal University, Huaibei 235000, China.

*Corresponding authors' emails: wangpl@chnu.edu.cn; hongjili@chnu.edu.cn

Table of Contents for Supporting Information

1. Experiment setup for electrochemical site-selective alkylation of azobenzenes with (thio)xanthenes ……………………………………………………S1
2. Gram-Scale Synthesis of **3a**……………………………………………..S1
3. Mechanistic Experiments…………………………………………..……S2
   3.1 Cyclic Voltammetry Studies ……………………………..…………S2
   3.2 KIE Experiment…………………………………………………..S2
4. ^{1}H, ^{13}C, ^{19}F NMR Spectra of the Products ……..…………………………..…..S6
5. Crystallographic Data for **3d** ………….………….…………….……….......S35
6. Determination of Faradaic Efficiency ……….………….………….…….......S36

1. Experiment setup for electrochemical site-selective alkylation of azobenzenes with (thio)xanthenes

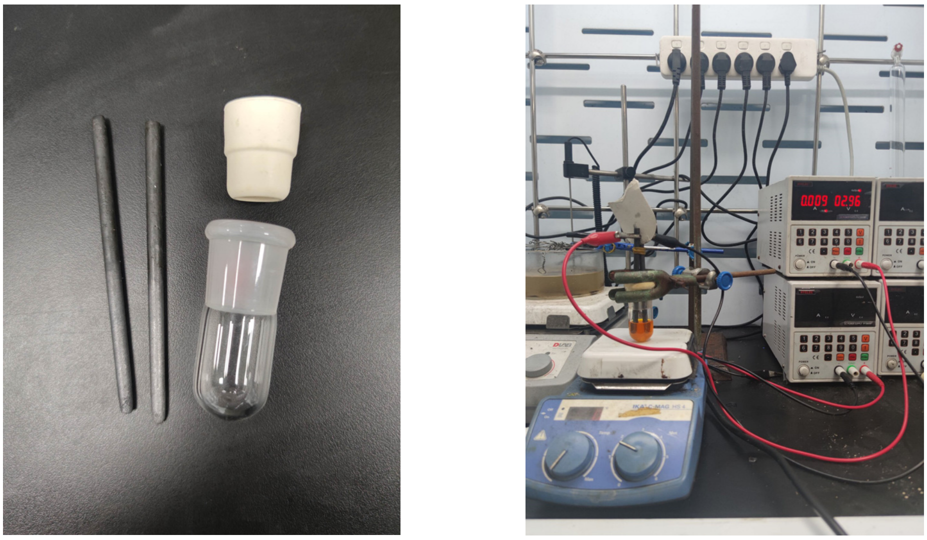

Figure S1 Experiment setup for electrochemical site-selective alkylation of azobenzenes with (thio)xanthenes

2. Gram-Scale Synthesis of 3a

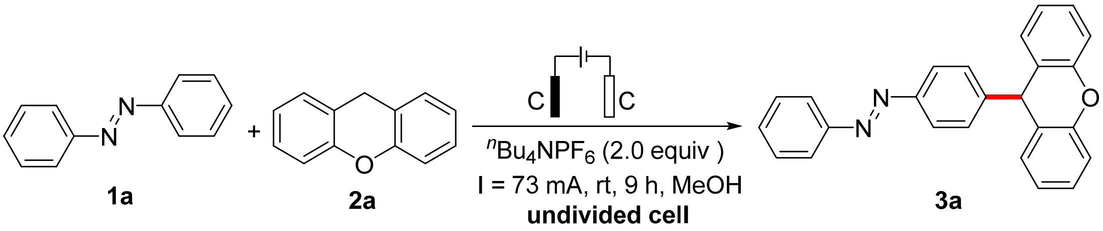

Azobenzene (**1a**, 1.0 g, 5.5 mmol, 1.0 equiv), xanthene (**2a**, 1.2 g, 6.6 mmol, 1.2 equiv), $^{n}Bu_4NPF_6$ (4.3 g, 11.0 mmol, 2.0 equiv) and MeOH (25 mL) was sequentially added to a 50 mL oven-dried undivided three neck bottle that equipped with a magnetic stirrer bar and sealed with rubber plugs under air atmosphere. A carbon rod (Φ 6 mm) anode and a carbon rod (Φ 6 mm) were used as the cathode in the bottle. About 1.0 cm of the carbon rod was under the solution. The reaction mixture was stirred and electrolyzed at a constant current of 73 mA under air at room temperature for 9 hours. After completion of the reaction, the solution was concentrated in vacuum. The resulting crude mixture was purified by flash column chromatography to give the desired product **3a** (1.2 g, 60 % yield).

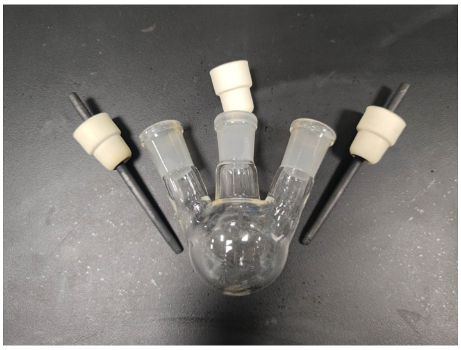

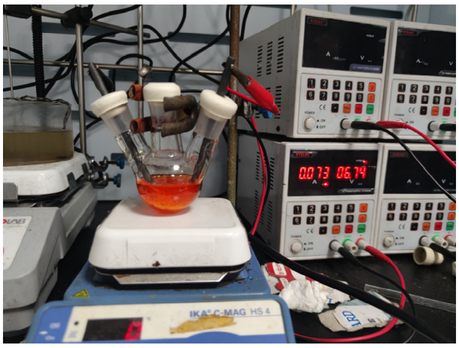

Figure S2 Experiment setup for the gram-scale synthesis of **3a**.

3. Mechanistic Experiments

3.1 Cyclic Voltammetry Studies

Cyclic voltammetry was performed in a three electrode cell connected to a Schlenk line at room temperature. The working electrode was a glassy carbon electrode, and the counter electrode was a platinum electrode. The reference was an Ag/AgCl electrode, and MeOH (10 mL) containing 0.1 M *n*-Bu_4NPF_6 was poured into the electrochemical cell in all experiments. Under ambient conditions, the LK98C electrochemical workstation was used with an applied potential range of 0 V to 3.0 V and a scan rate of 0.1 V/s. The test concentrations of **1a**, **2a** and **1a+2a** are 1 mM, respectively.

3.2 KIE experiments

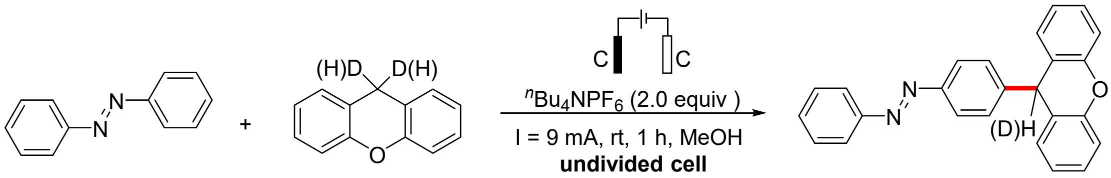

1a **2a/2a-d2** **3a+3a-d, ~25% yield**

Azobenzene **1a** (0.3 mmol，1.0 equiv), **2a** (0.18 mmol, 0.6 equiv), **2a-d2** (0.18 mmol, 0.6 equiv), $^nBu_4NPF_6$ (0.6 mmol, 2.0 equiv) and MeOH (5.0 mL) were sequentially added into a 15.0 mL oven-dried undivided single necked bottle that equipped with a magnetic stirrer bar and sealed with rubber plugs under air atmosphere. A carbon rod (Φ 6 mm) anode and a carbon rod (Φ 6 mm) were used as the cathode in the bottle. About 1.0 cm of the carbon rod was under the solution. The

reaction mixture was stirred and electrolyzed at a constant current of 9 mA under air at room temperature for 1 h. After that, the mixture in reaction tube was detected by TLC. The crude product was purified by flash chromatography (silica gel, petroleum ether), affording the desired product **3a** and **3a-d** as an orange solid (27.4 mg, 25% yield). The ratio of **3a**/**3a-d** was determined as 1.2 by ^{1}H NMR analysis.

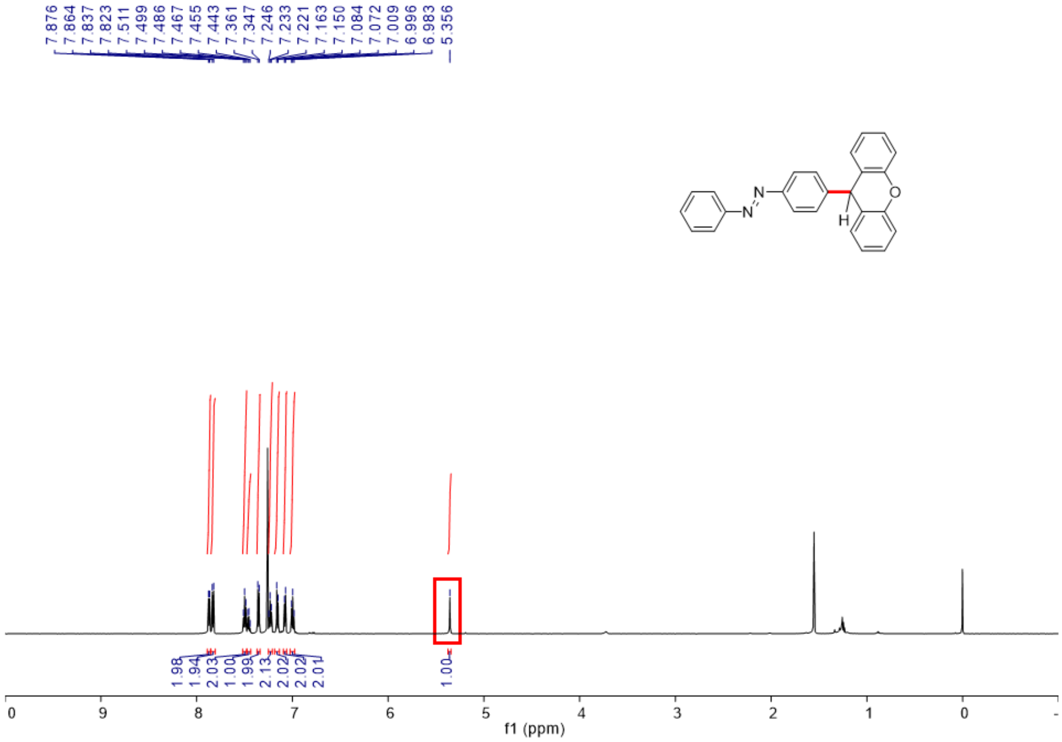

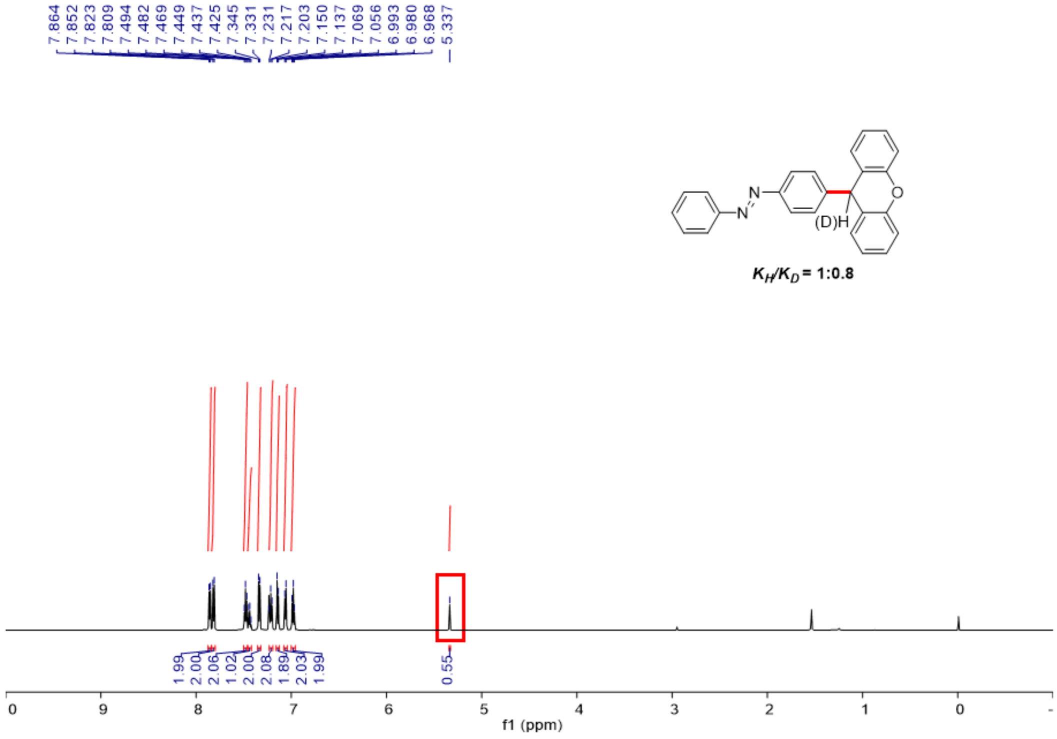

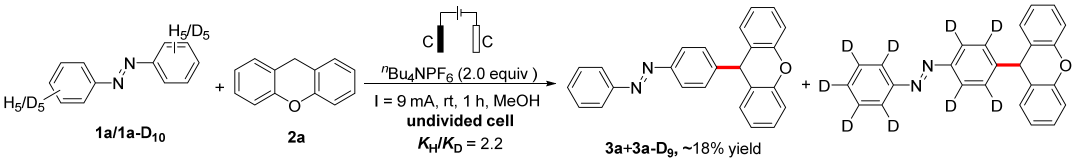

1a (0.15 mmol，0.5 equiv), **1a-d$_{10}$** (0.15 mmol，0.5 equiv), xanthene **2a** (0.36 mmol, 1.0 equiv), ^{n}Bu$_4$NPF$_6$ (0.6 mmol, 2.0 equiv) and MeOH (5.0 mL) were sequentially added into a 15.0 mL oven-dried undivided single necked bottle that equipped with a magnetic stirrer bar and sealed with rubber plugs under air atmosphere. A carbon rod (Φ 6 mm) anode and a carbon rod (Φ 6 mm) were used as the cathode in the bottle. About 1.0 cm of the carbon rod was under the solution. The reaction mixture was stirred and electrolyzed at a constant current of 9 mA under air at room temperature for 1 h. After that, the mixture in reaction tube was detected by TLC. The crude product was purified by flash chromatography (silica gel, petroleum ether), affording the desired product **3a** and **3a-D$_9$** as an orange solid (19.8 mg, 18% yield). The ratio of **3a**/**3a-D$_9$** was determined as 2.2 by ^{1}H NMR analysis.

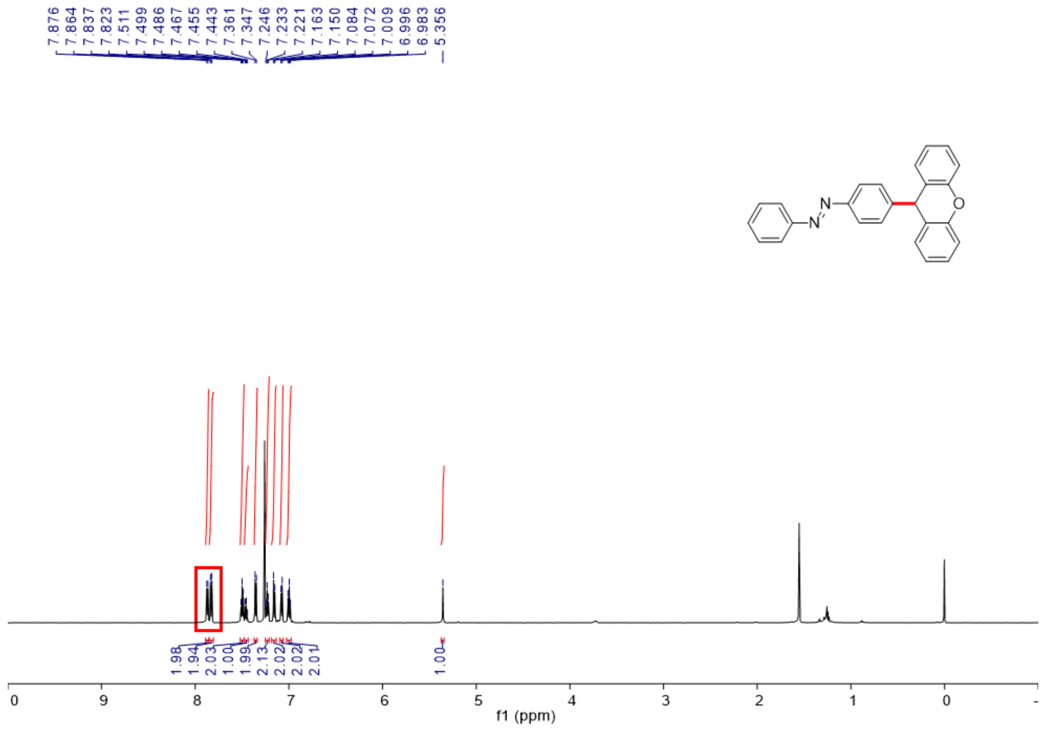
7.876
7.864
7.837
7.823
7.511
7.499
7.486
7.467
7.455
7.443
7.361
7.347
7.246
7.233
7.221
7.163
7.150
7.084
7.072
7.009
6.996
6.983
5.356
N
N
O
1.98
1.94
2.03
1.00
1.99
2.13
2.02
2.02
2.01
1.00
f1 (ppm)

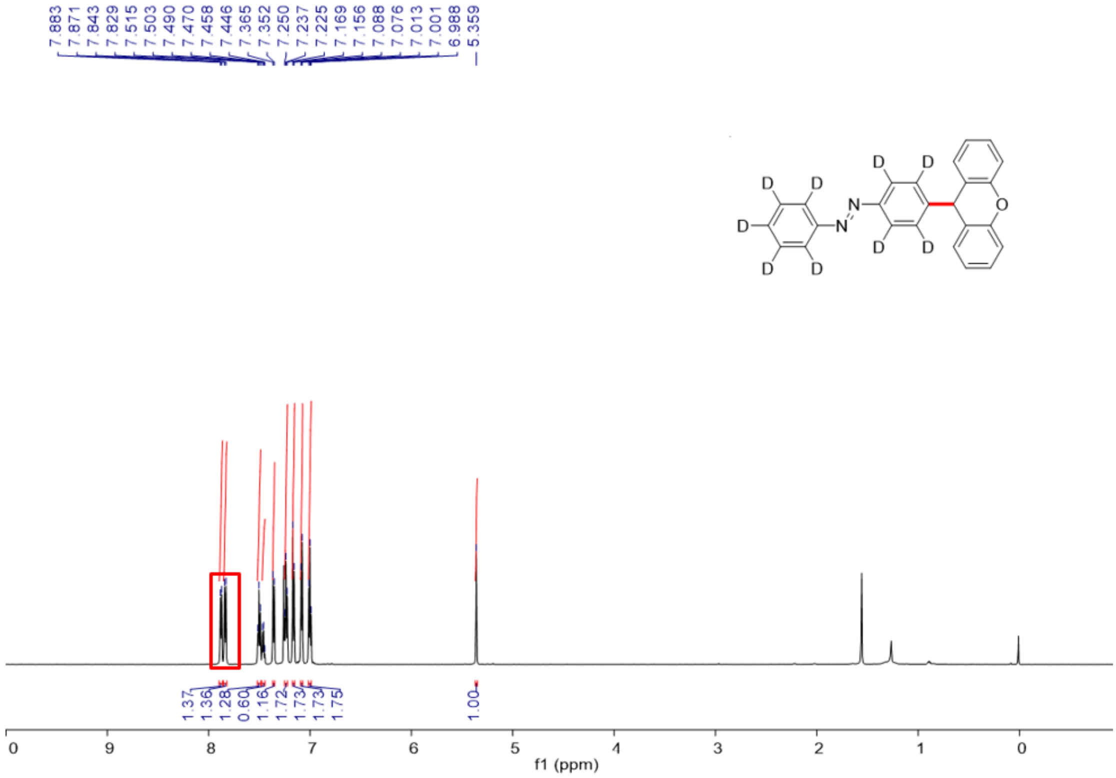
7.883
7.871
7.843
7.829
7.515
7.503
7.490
7.470
7.458
7.446
7.365
7.352
7.250
7.237
7.225
7.169
7.156
7.088
7.076
7.013
7.001
6.988
5.359
D
N
N
O
1.37
1.36
1.28
0.60
1.16
1.72
1.73
1.73
1.75
1.00
f1 (ppm)

4. ^{1}H, ^{13}C, ^{19}F NMR Spectra of the Products

NMR spectra of (*E*)-1-(4-(9*H*-xanthen-9-yl)phenyl)-2-phenyldiazene (**3a**)

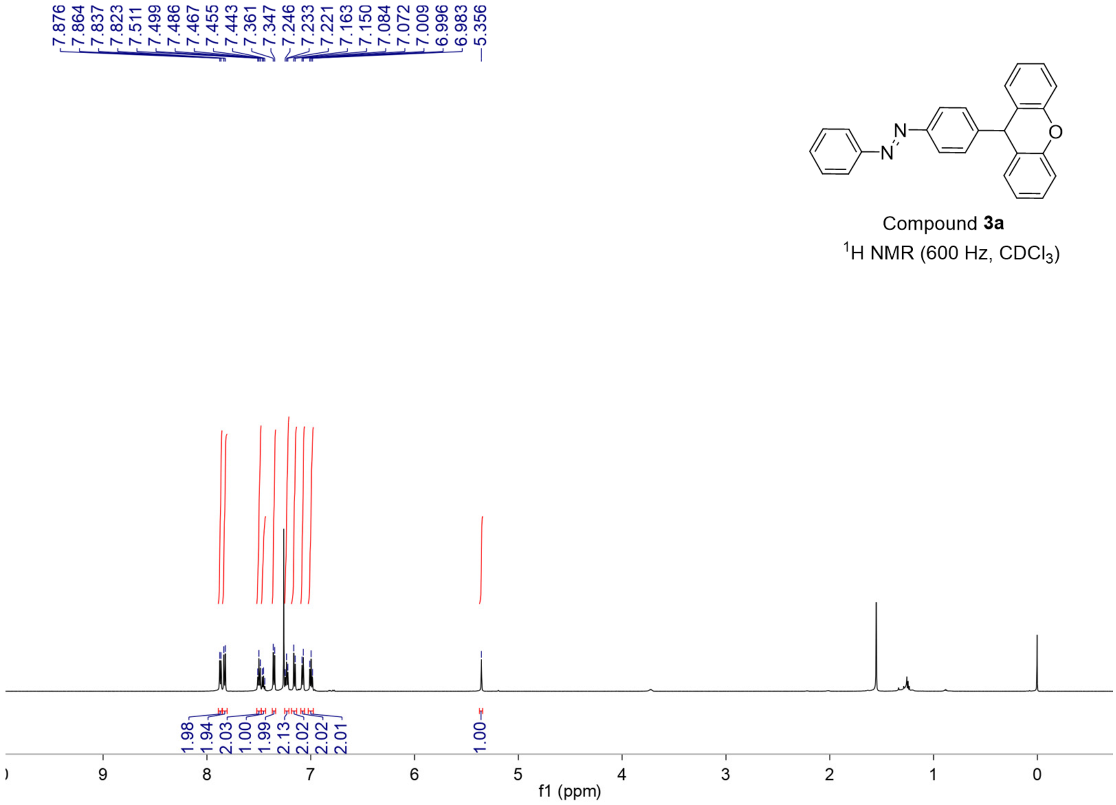

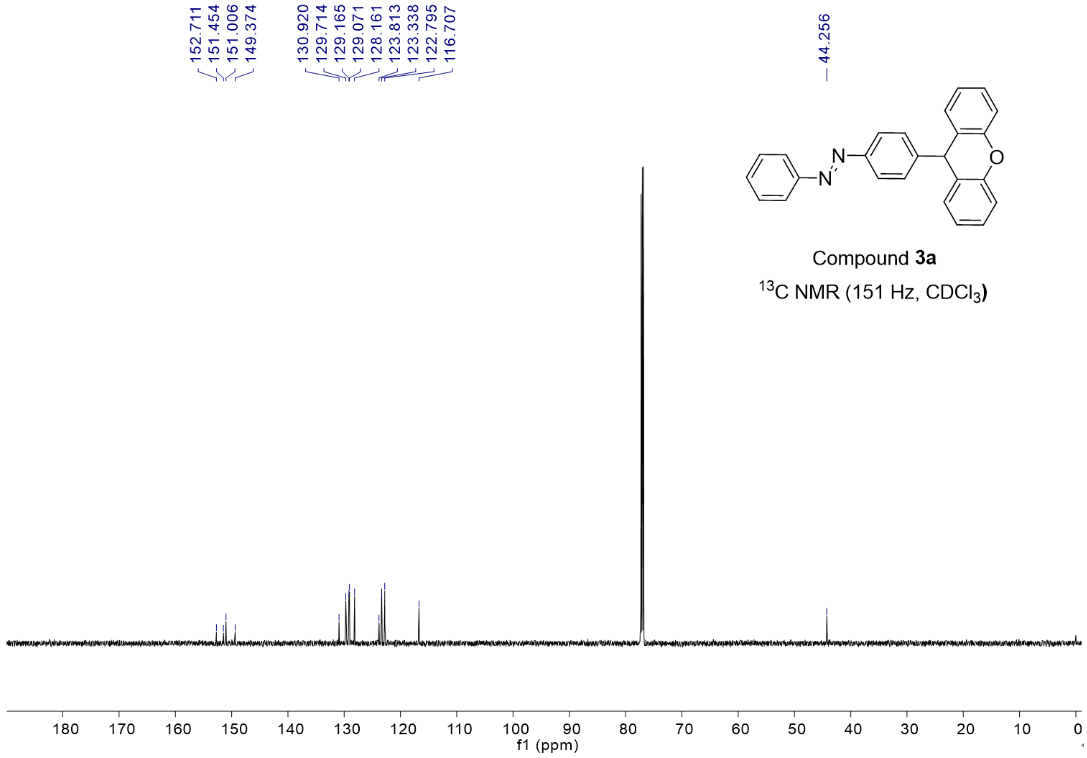

NMR spectra of (*E*)-1-(4-(9*H*-xanthen-9-yl)phenyl)-2-(*p*-tolyl)diazene (**3b**)

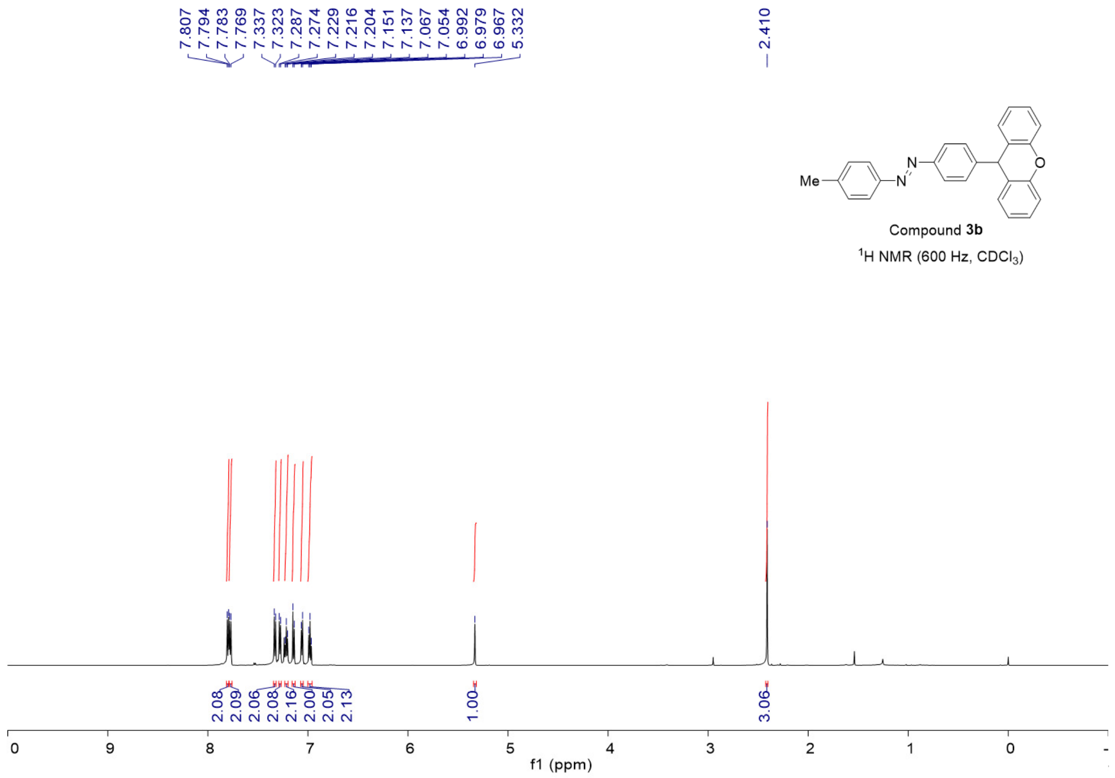

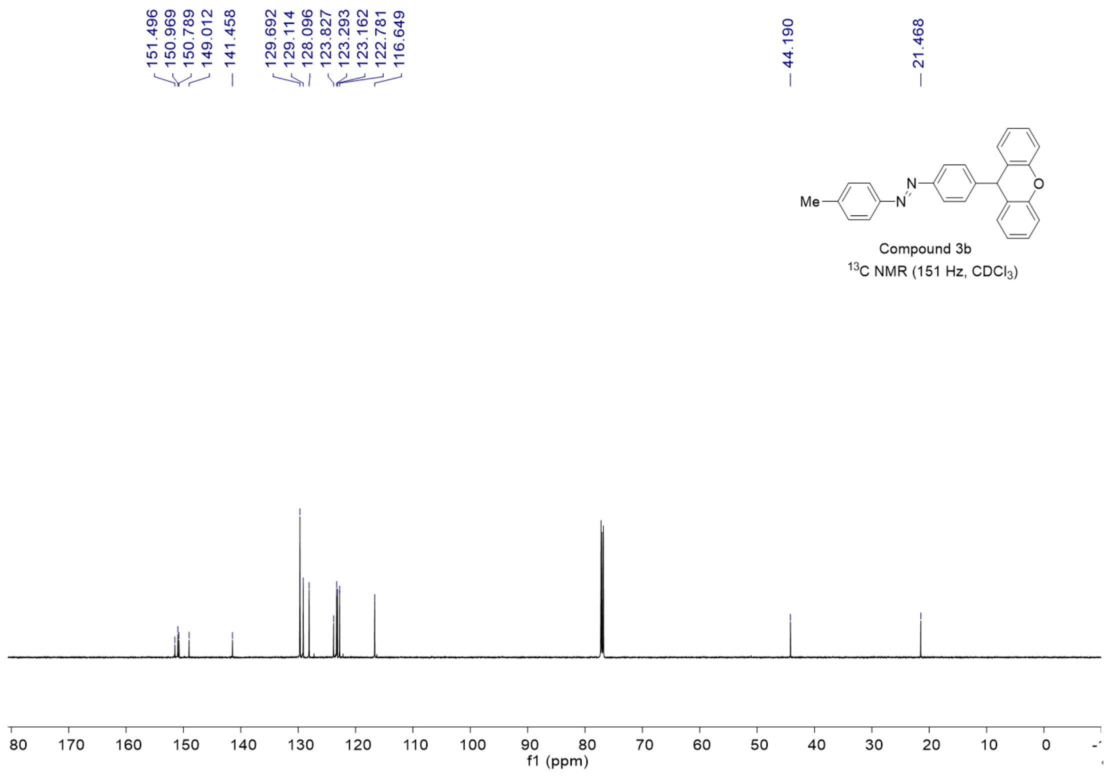

NMR spectra of (*E*)-1-(4-(9*H*-xanthen-9-yl)phenyl)-2-(4-ethylphenyl)diazene (**3c**)

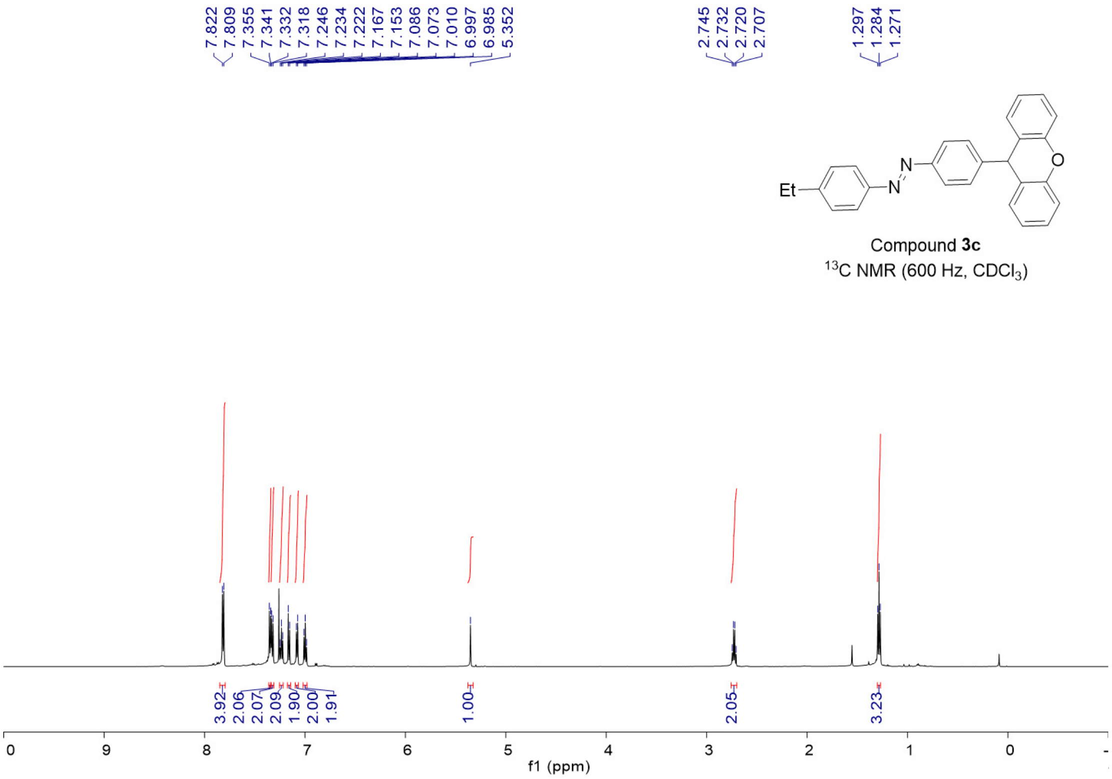

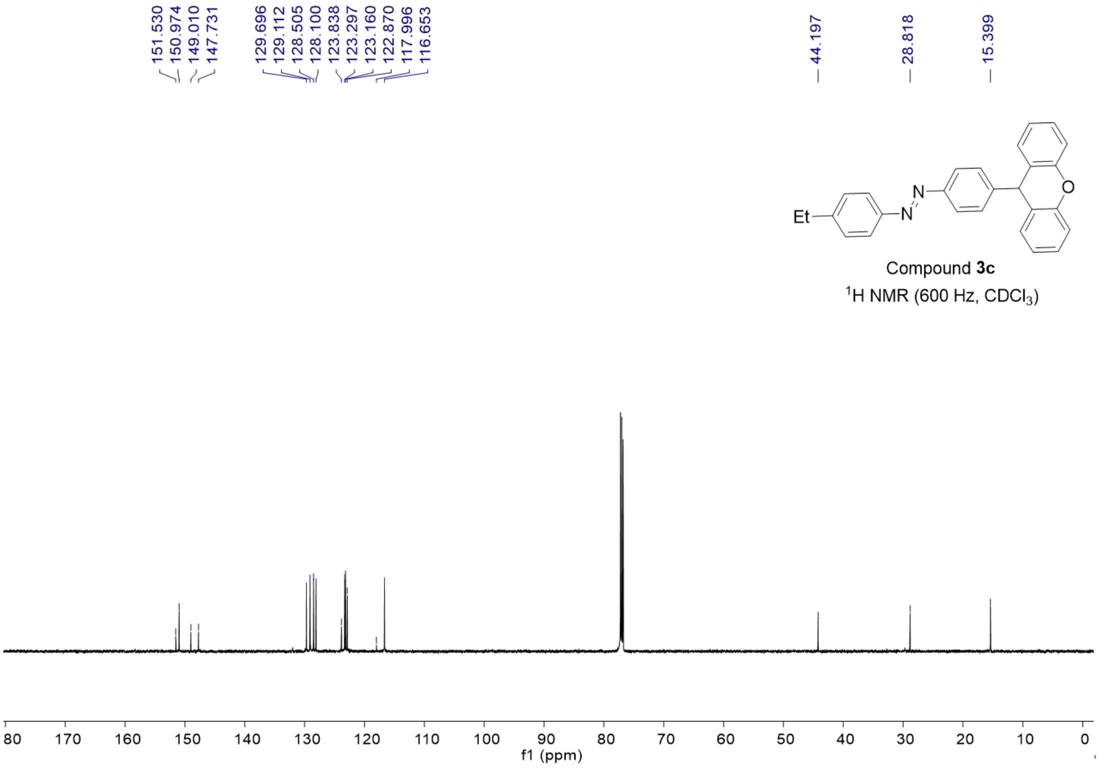

NMR spectra of (*E*)-1-(4-(9*H*-xanthen-9-yl)phenyl)-2-(4-isopropylphenyl)diazene (**3d**)

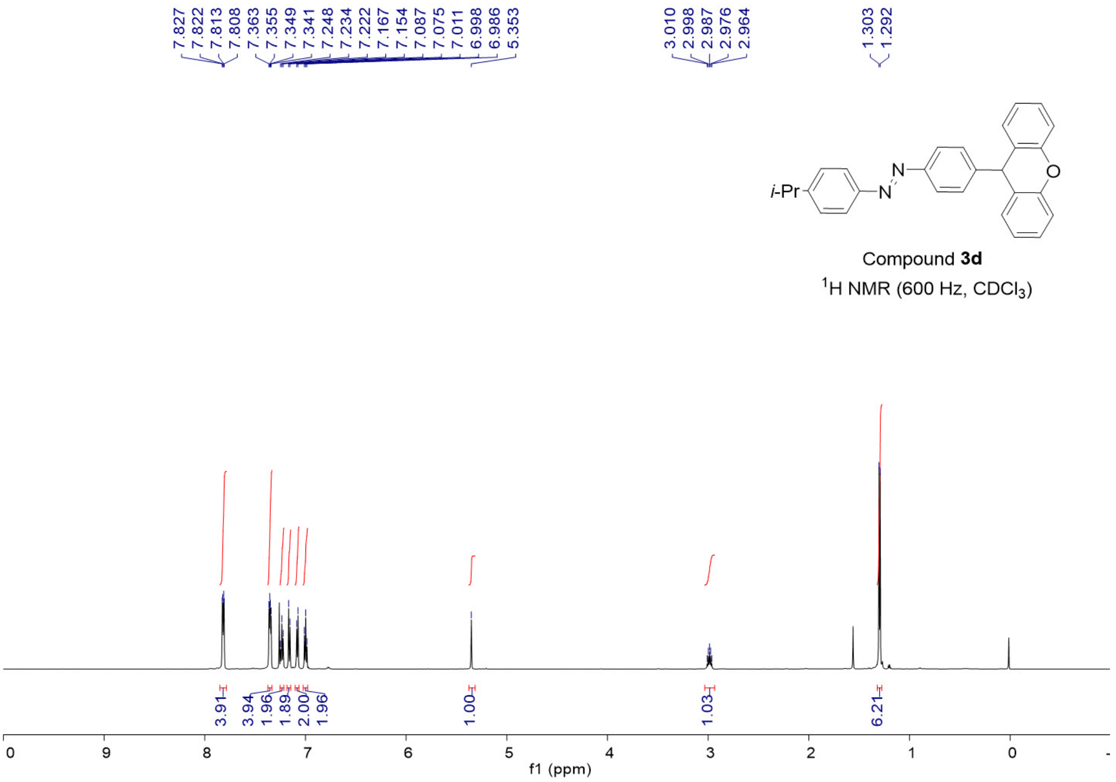

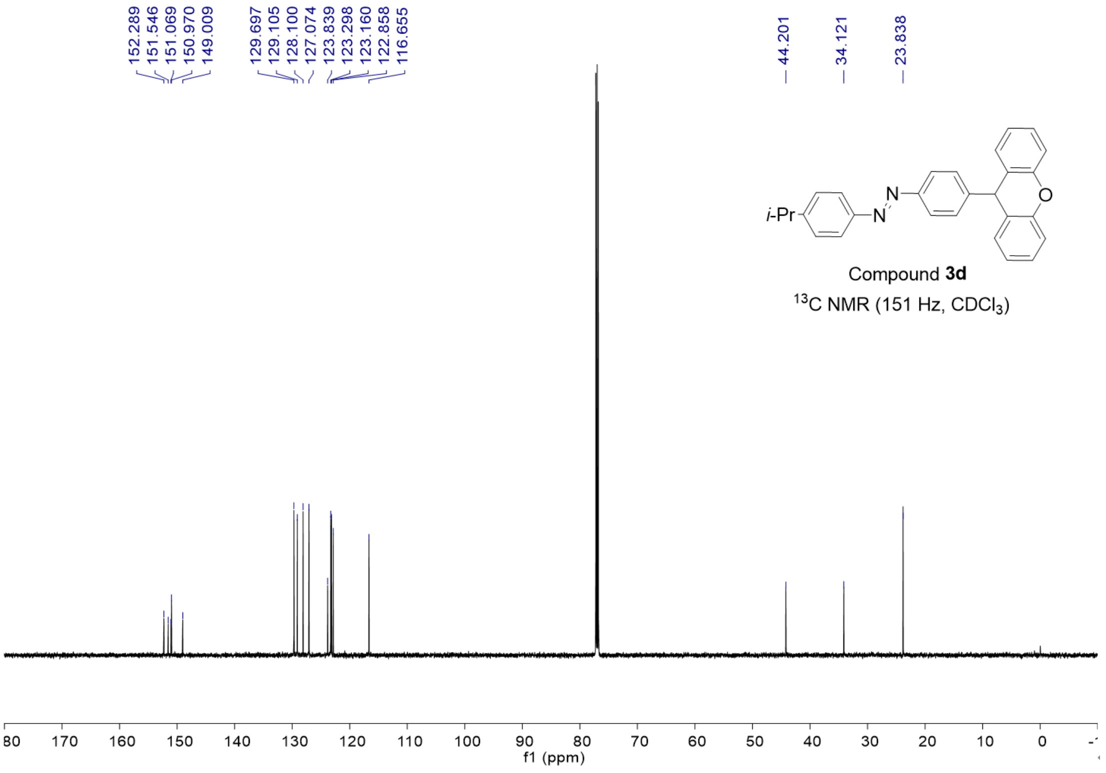

NMR spectra of (*E*)-1-(4-(9*H*-xanthen-9-yl)phenyl)-2-(4-(*tert*-butyl)phenyl)diazene (**3e**)

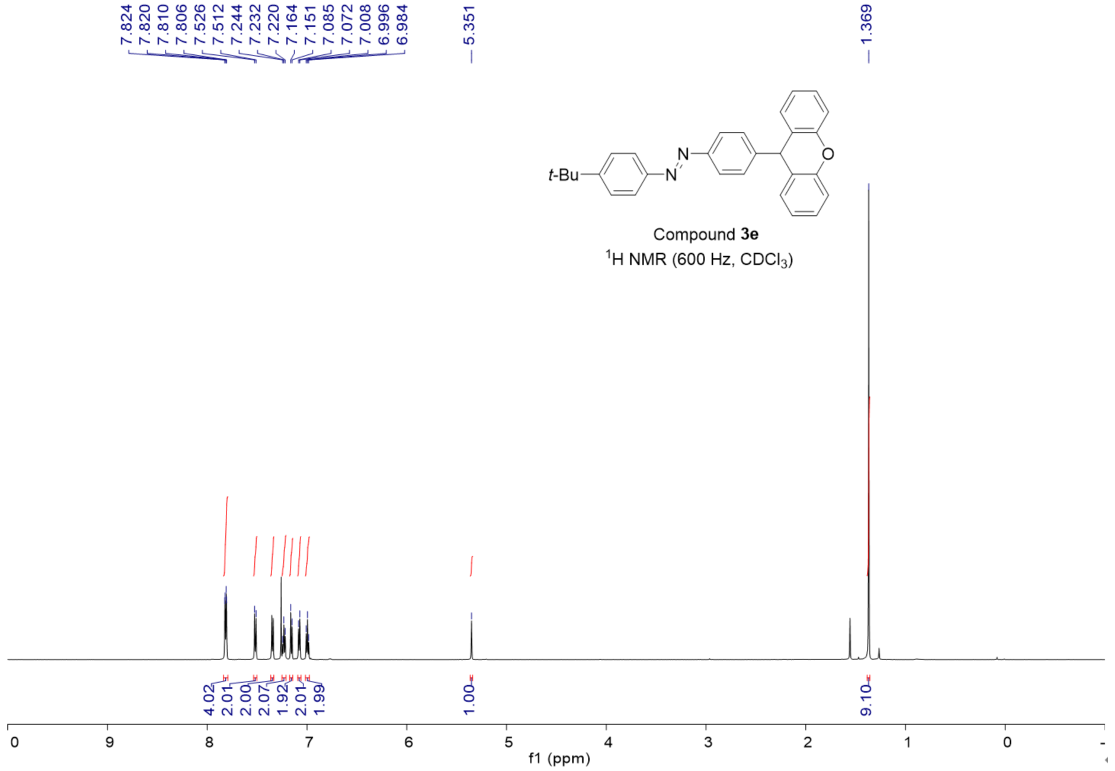

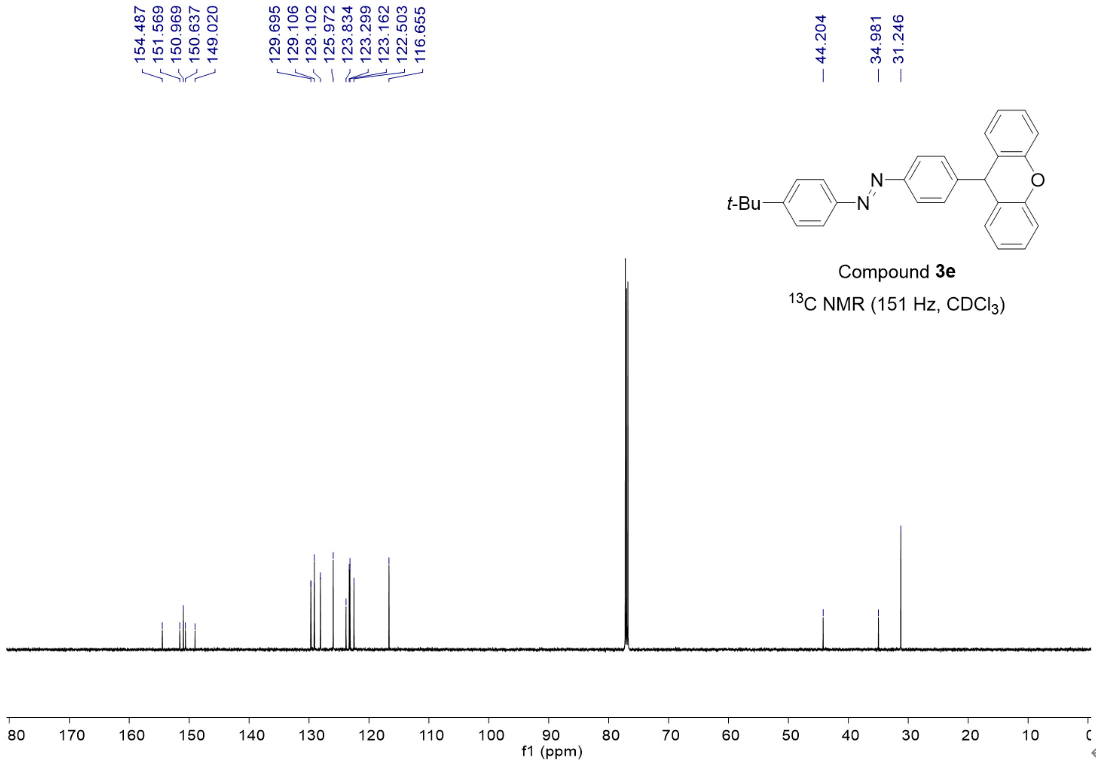

NMR spectra of (*E*)-1-(4-(9*H*-xanthen-9-yl)phenyl)-2-(4-(trifluoromethoxy)phenyl)diazene **(3f)**

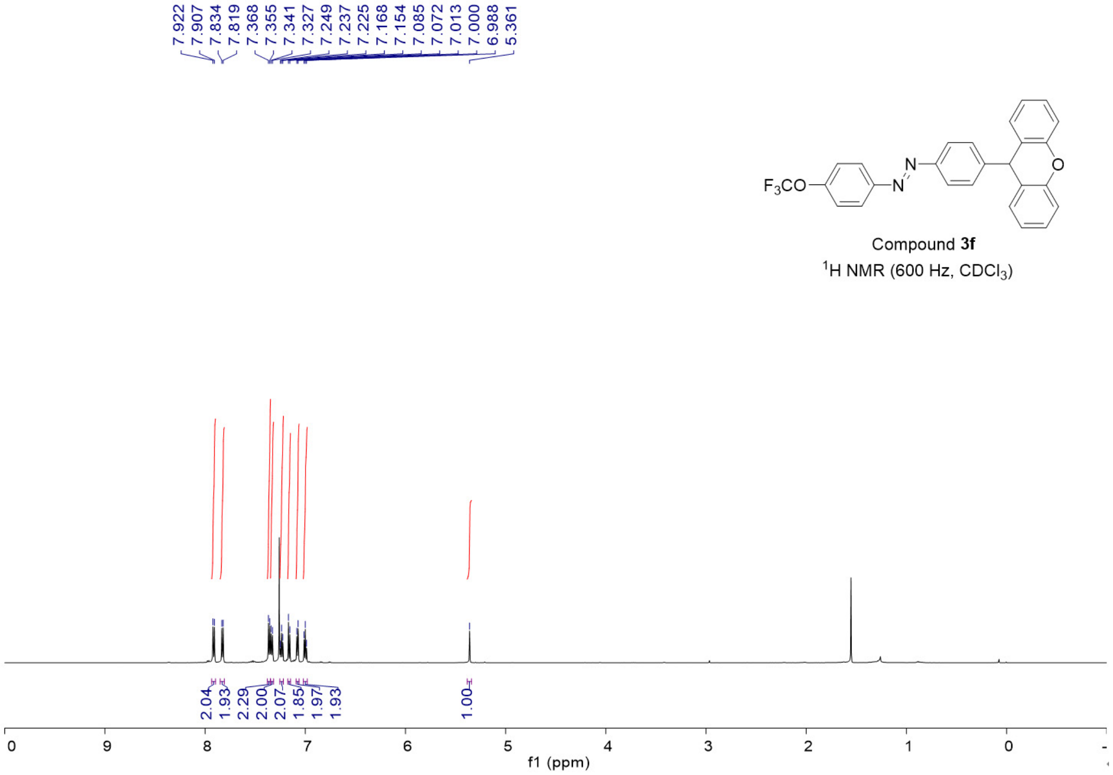

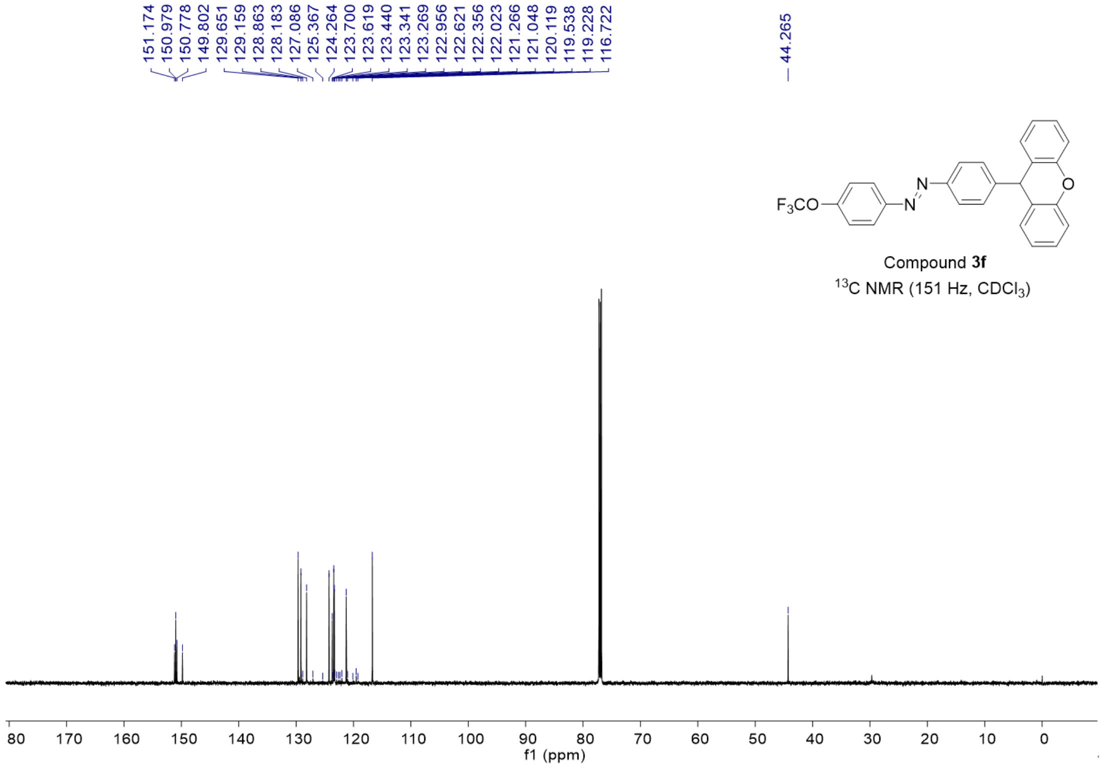

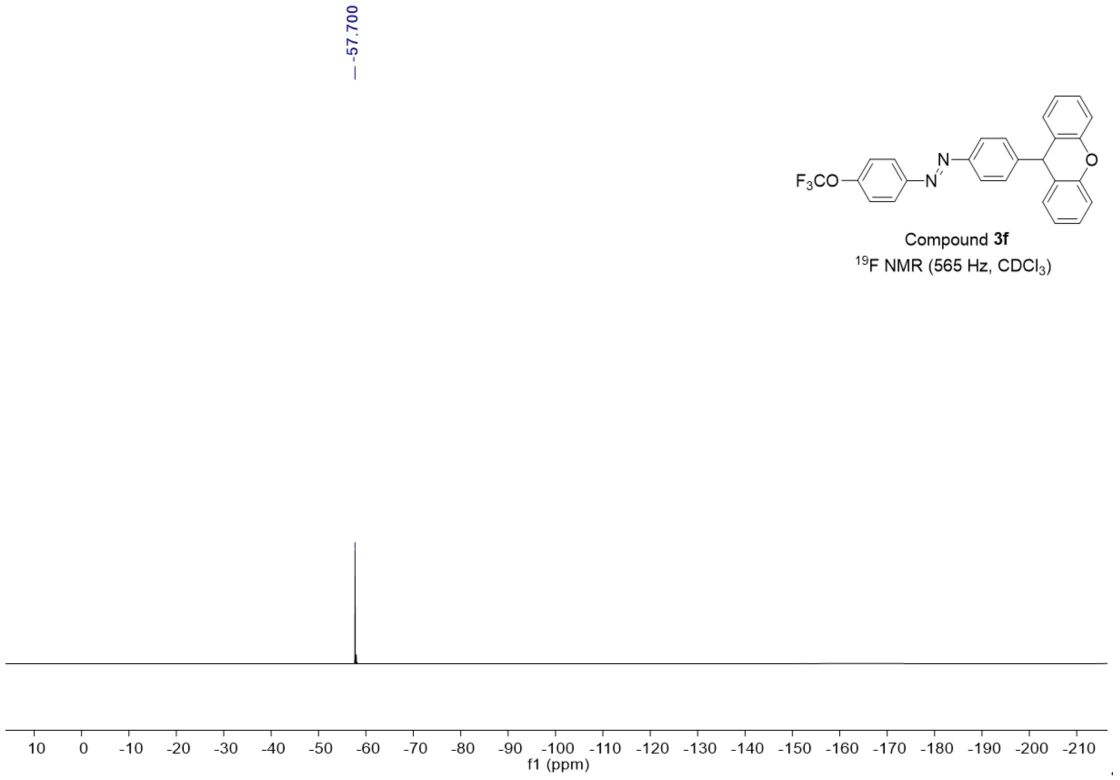

NMR spectra of (*E*)-1-(4-((4-(9*H*-xanthen-9-yl)phenyl)diazenyl)phenyl)ethan-1-one (**3g**)

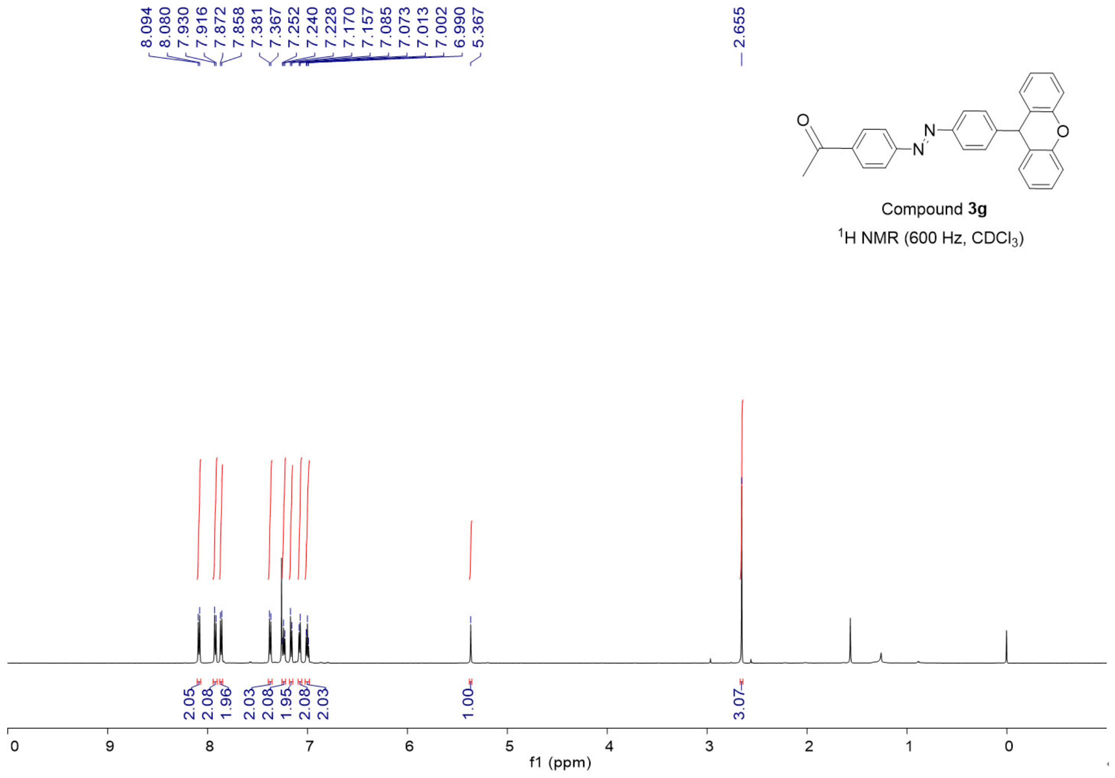

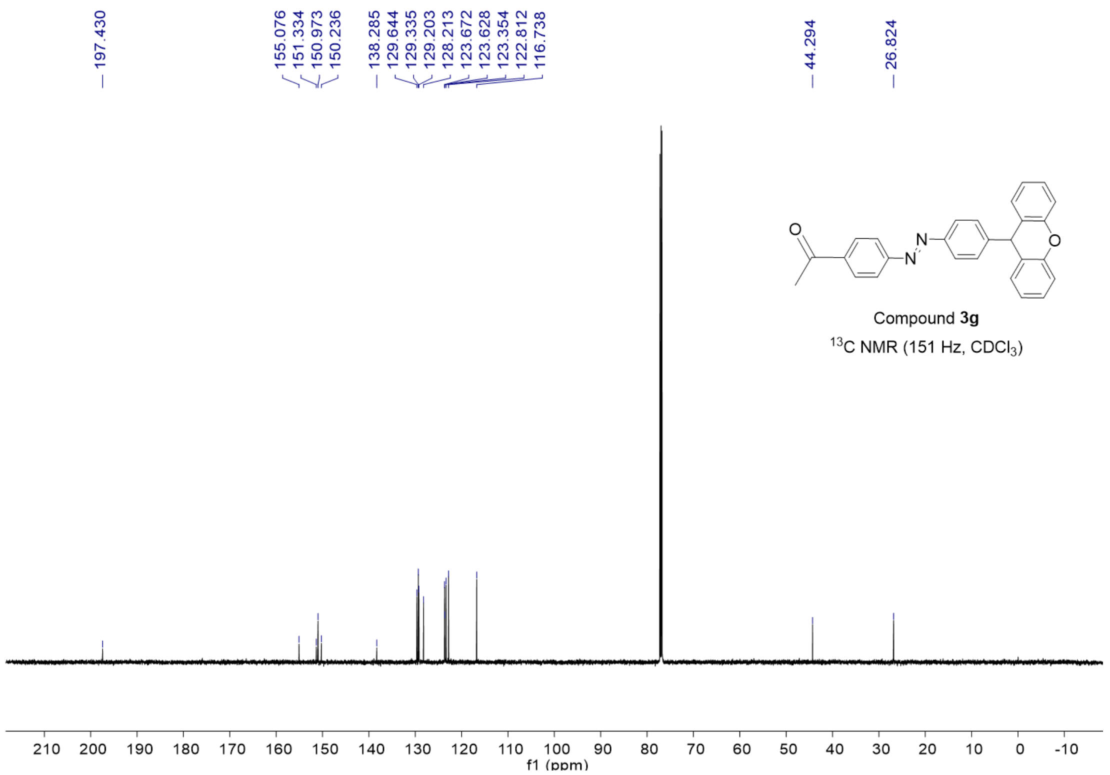

NMR spectra of (*E*)-4-((4-(9*H*-xanthen-9-yl)phenyl)diazenyl)benzonitrile (**3h**)

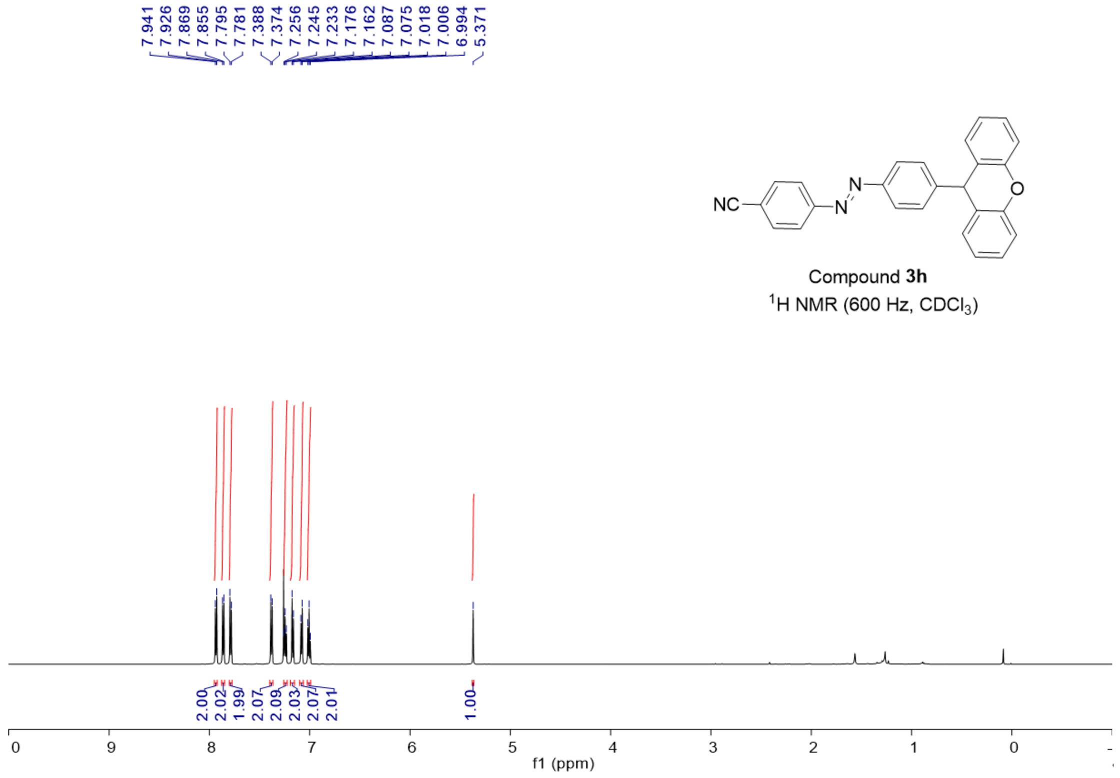

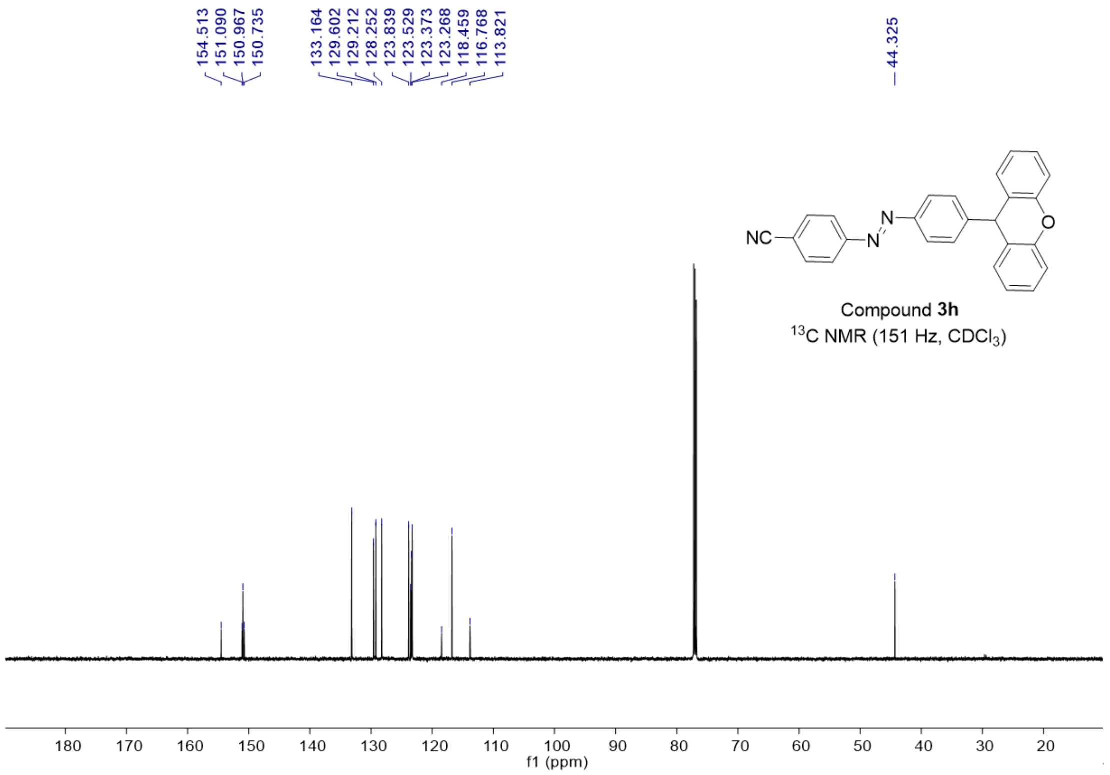

NMR spectra of (*E*)-1-(4-(9*H*-xanthen-9-yl)phenyl)-2-(4-(trifluoromethyl)phenyl)diazene (**3i**)

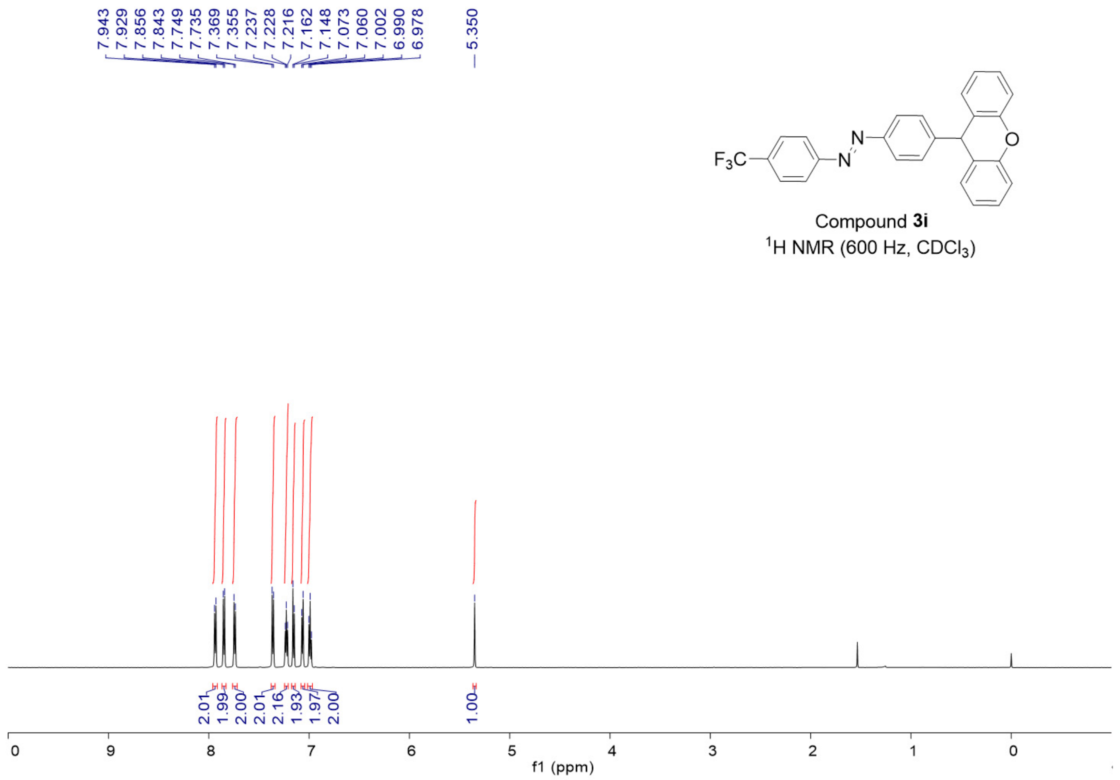

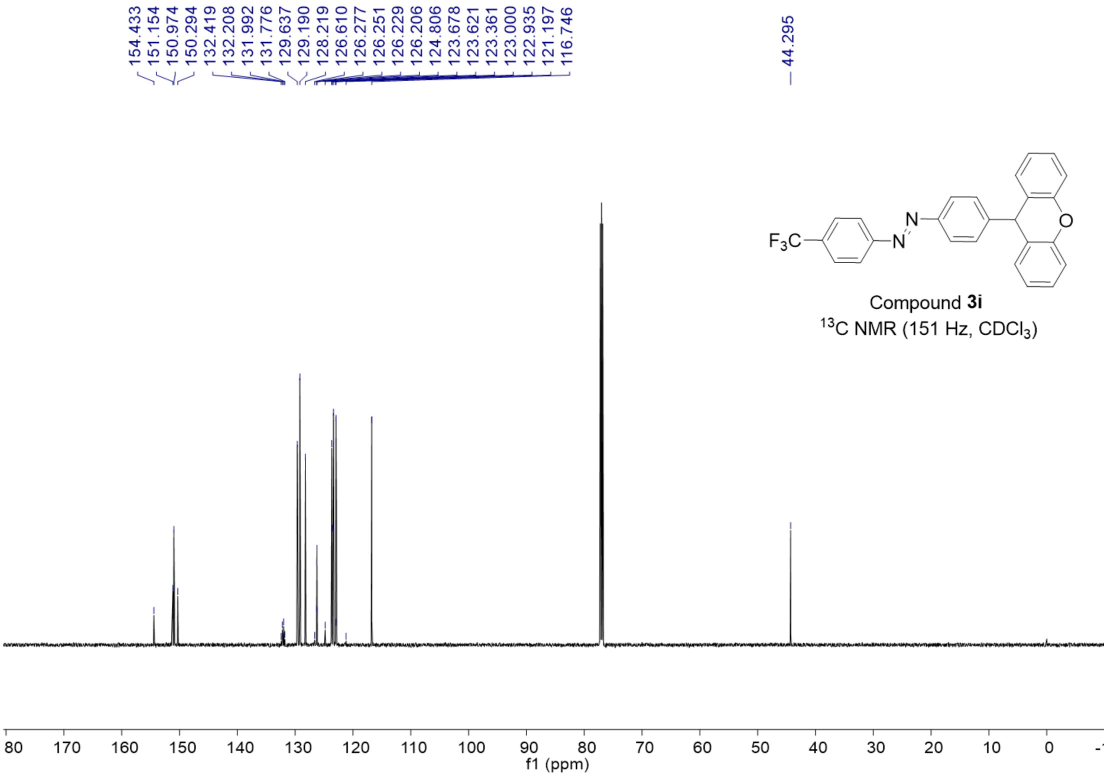
154.433
151.154
150.974
150.294
132.419
132.208
131.992
131.776
129.637
129.190
128.219
126.610
126.277
126.251
126.229
126.206
124.806
123.678
123.621
123.361
123.000
122.935
121.197
116.746
44.295
F_3C
N
N
O
Compound **3i**
^{13}C NMR (151 Hz, $CDCl_3$)
f1 (ppm)

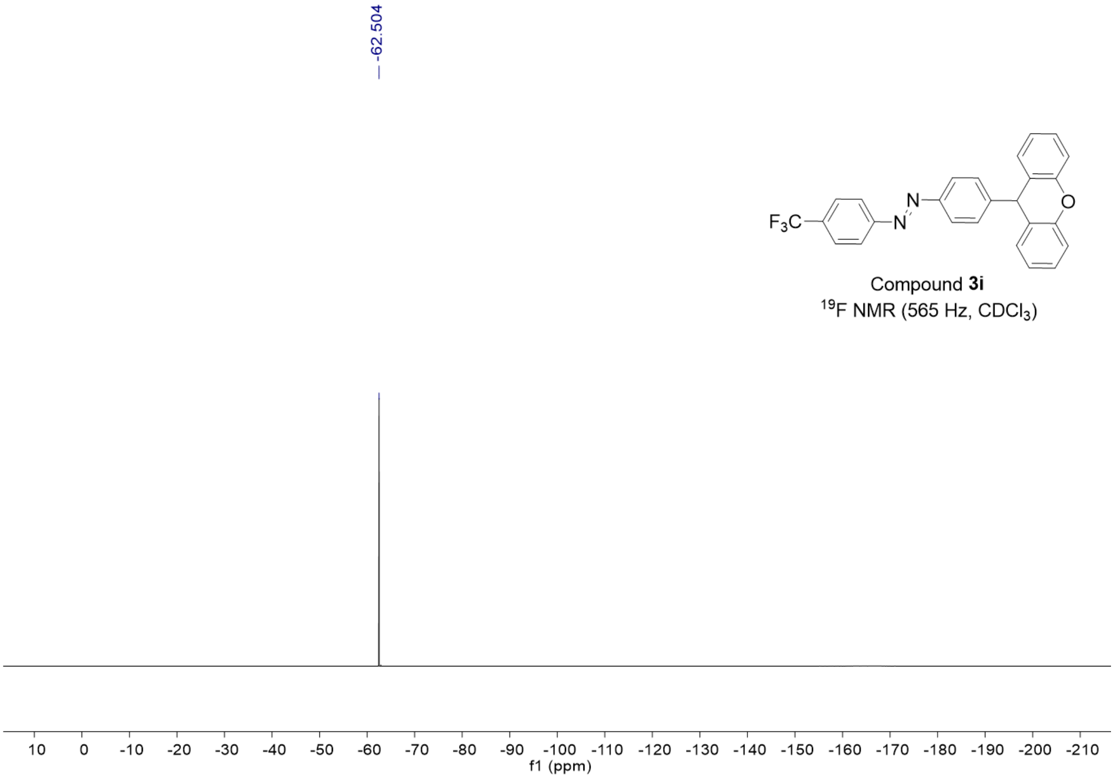
-62.504
F_3C
N
N
O
Compound **3i**
^{19}F NMR (565 Hz, $CDCl_3$)
f1 (ppm)

NMR spectra of (*E*)-1-(4-(9H-xanthen-9-yl)phenyl)-2-(m-tolyl)diazene **(3k);** (*E*)-1-(3-methyl-4-(9H-xanthen-9-yl)phenyl)-2-phenyldiazene **(3k')**

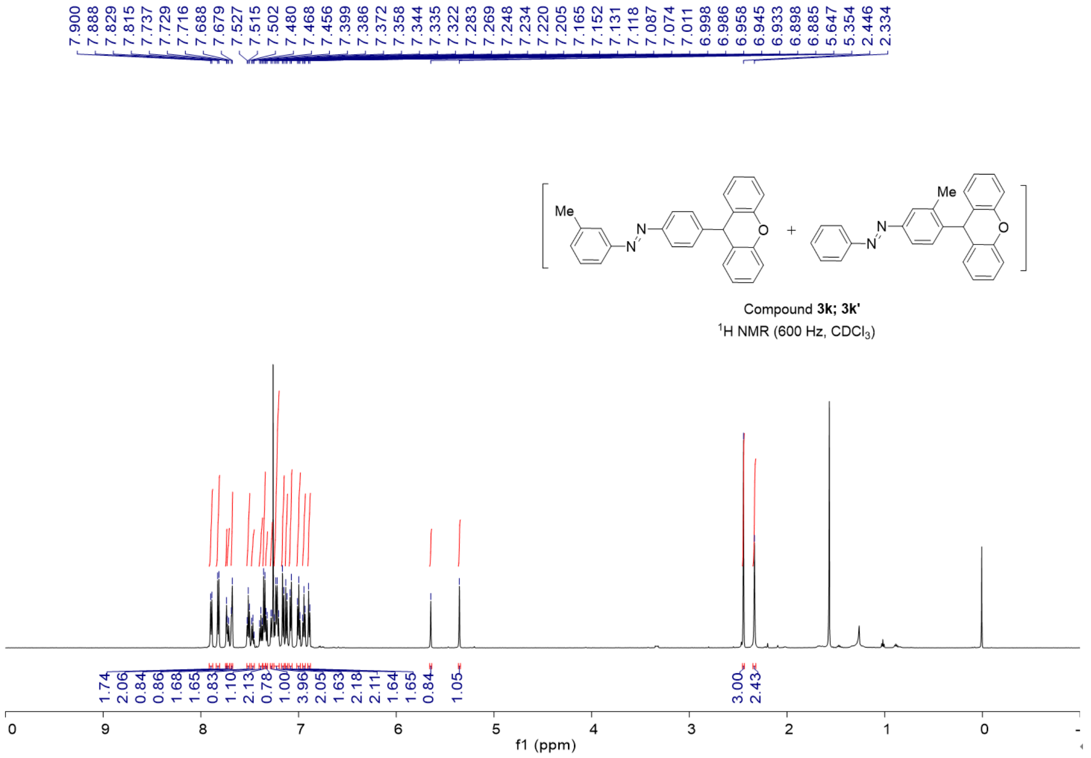

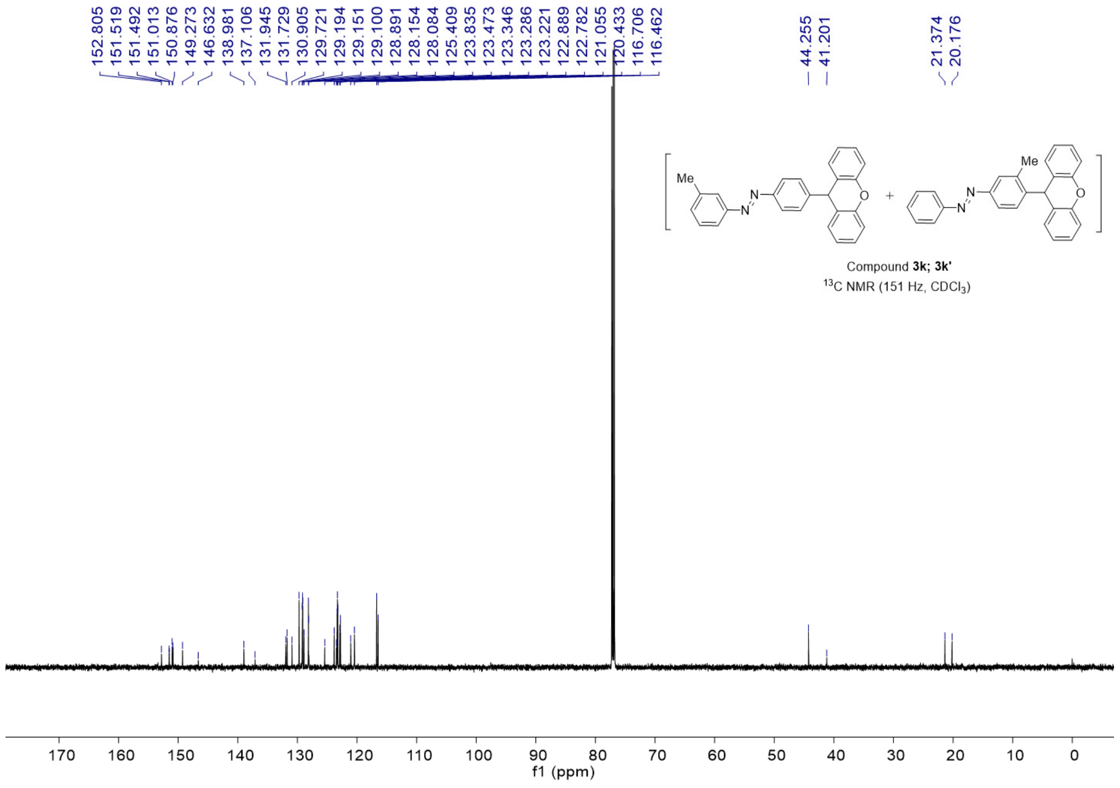

NMR spectra of (*E*)-1-(4-(9*H*-xanthen-9-yl)phenyl)-2-(2,3-dimethylphenyl)diazene (**3l**)

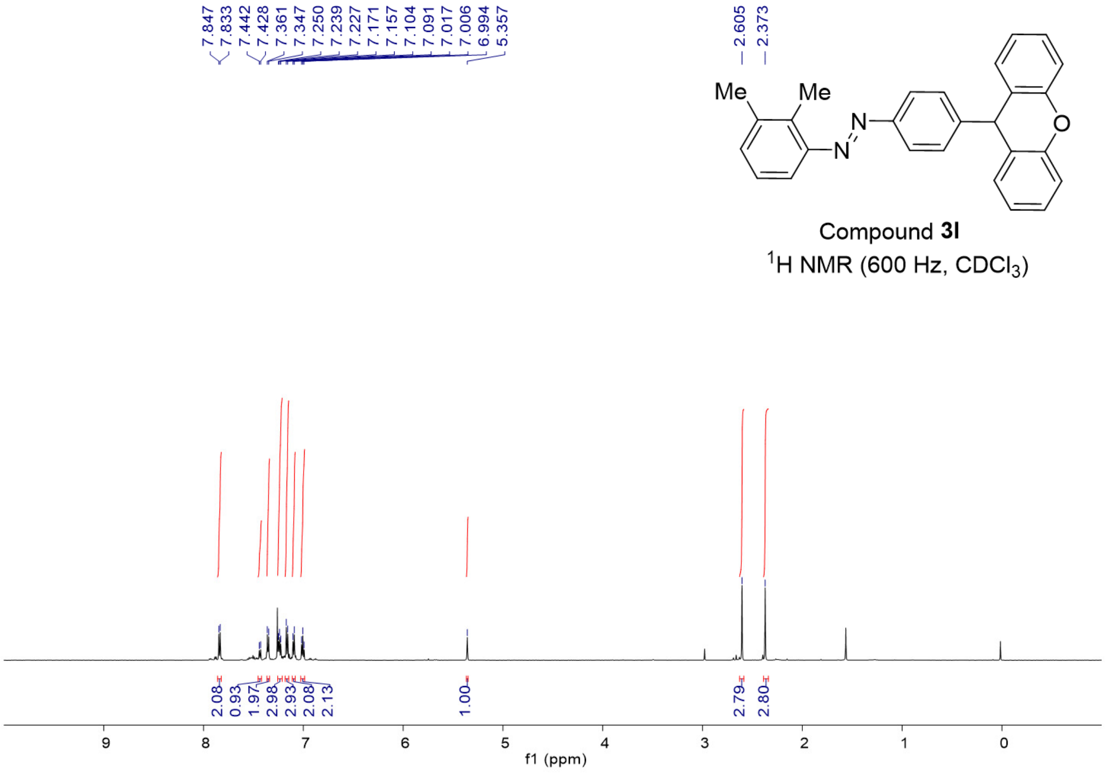

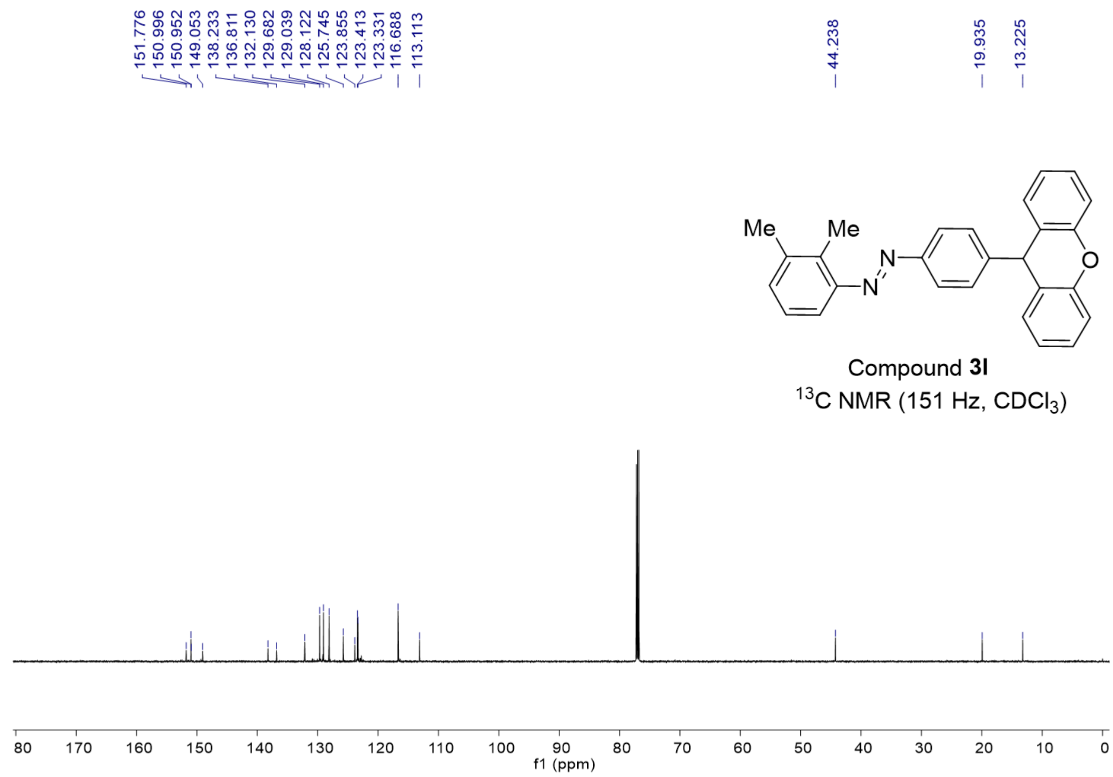

NMR spectra of (*E*)-1-(4-(9*H*-xanthen-9-yl)phenyl)-2-(2,4-dimethy phenyl)diazene (**3m**)

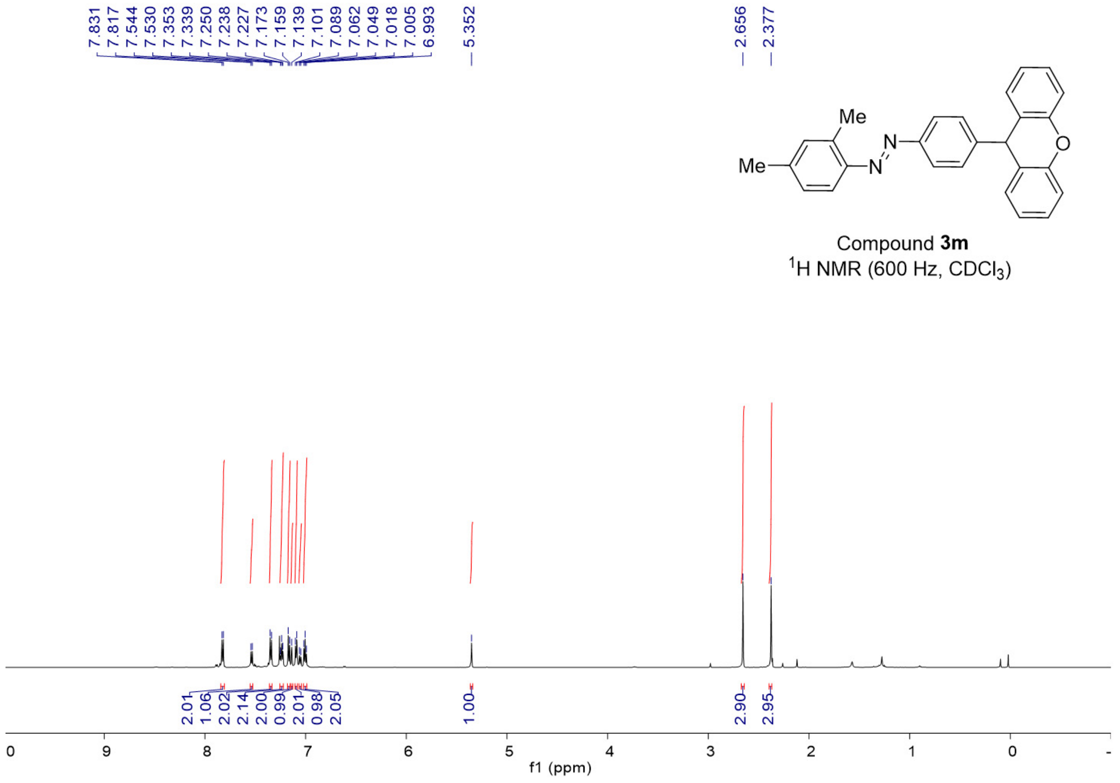

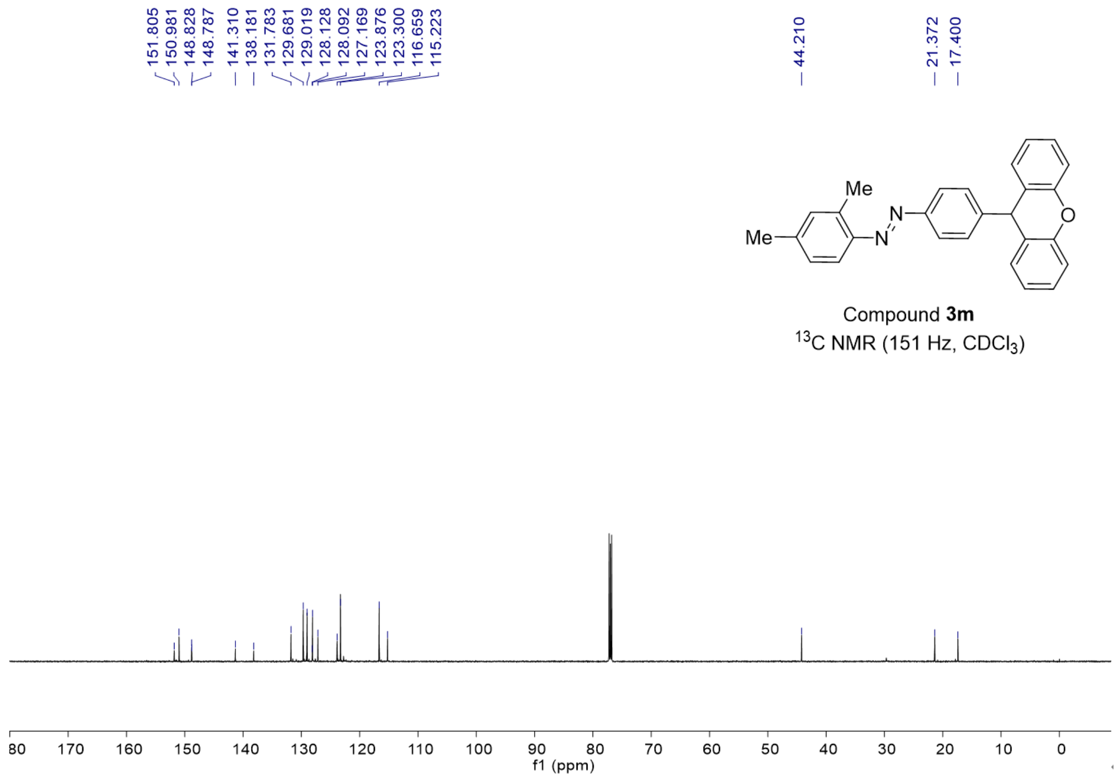

NMR spectra of (*E*)-1-(4-(9*H*-xanthen-9-yl)phenyl)-2-(3,4-dimethylphenyl)diazene (**3n**)

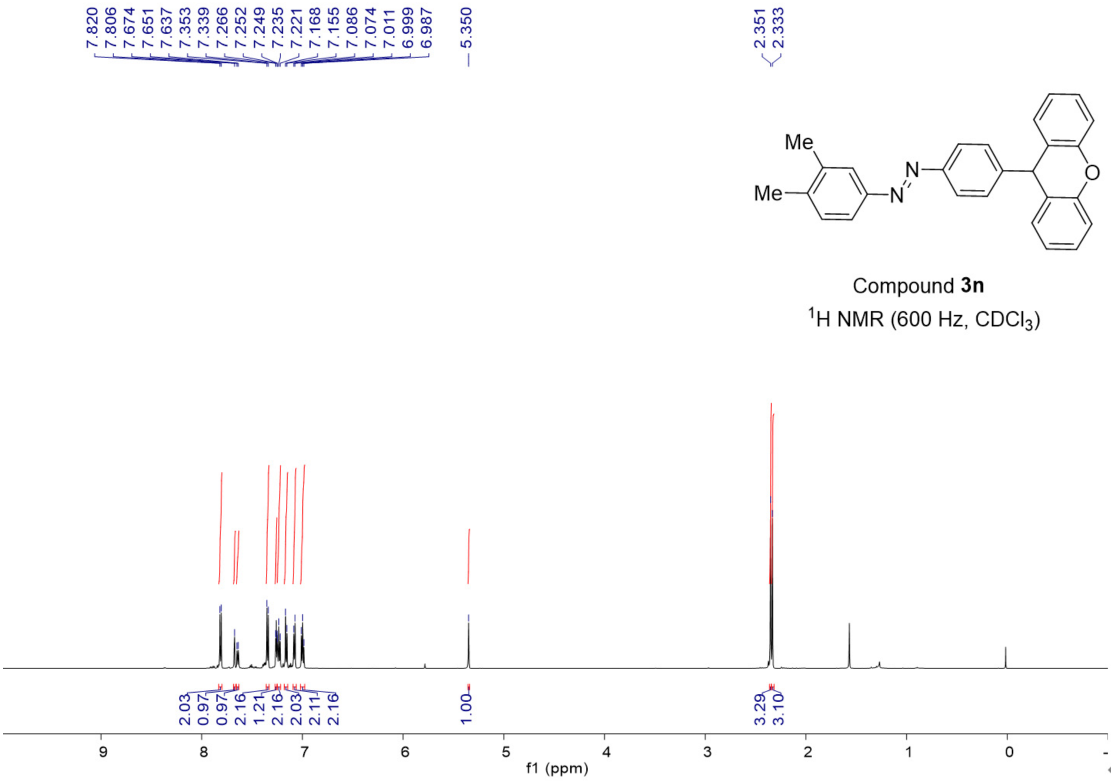

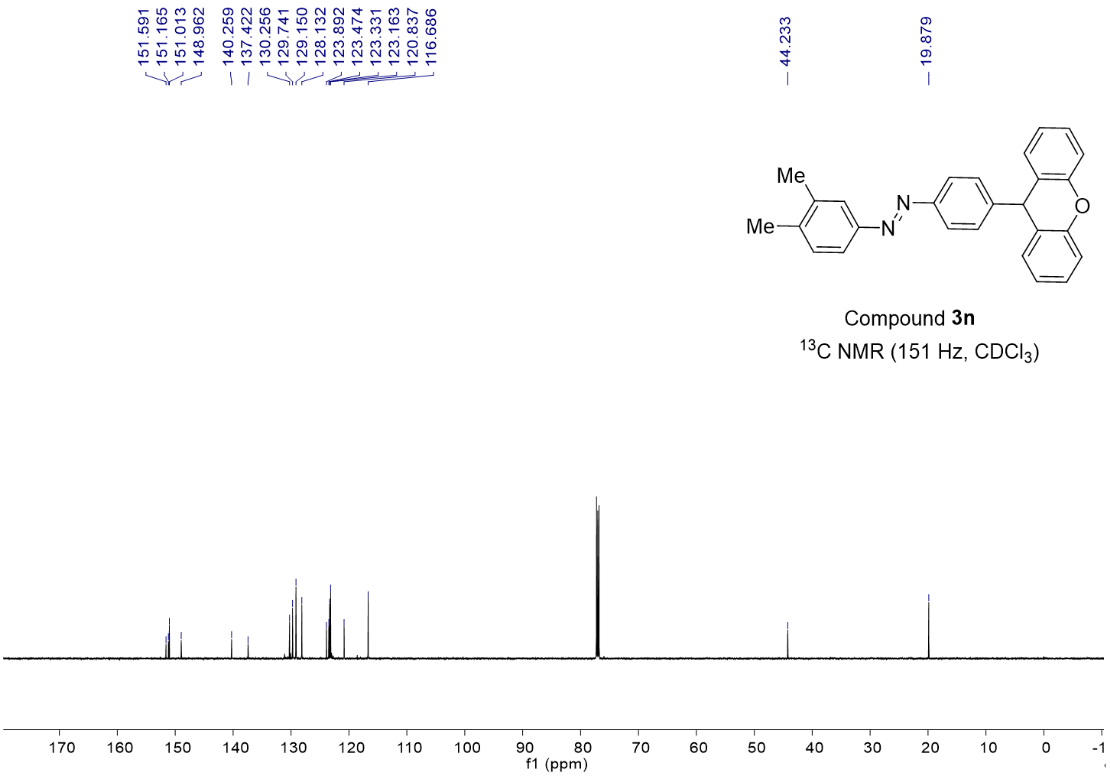

NMR spectra of (*E*)-1-(4-(9*H*-xanthen-9-yl)phenyl)-2-(3,5-dimethylphenyl)diazene (**3o**)

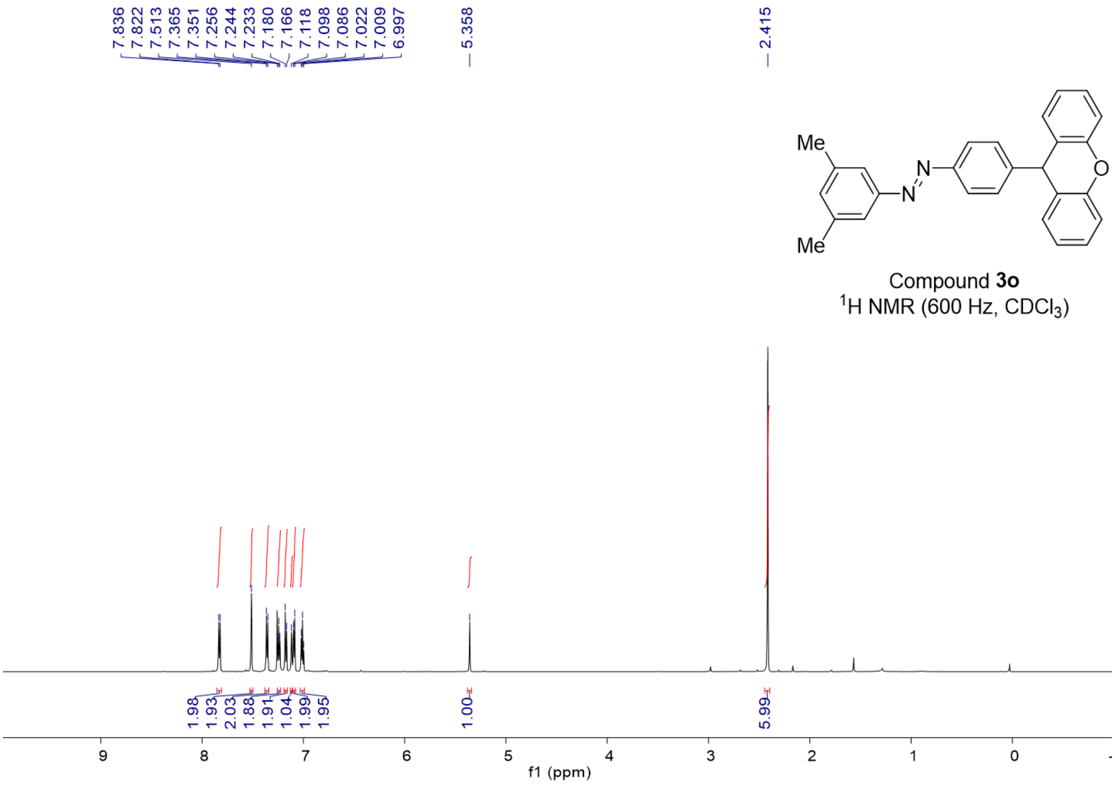

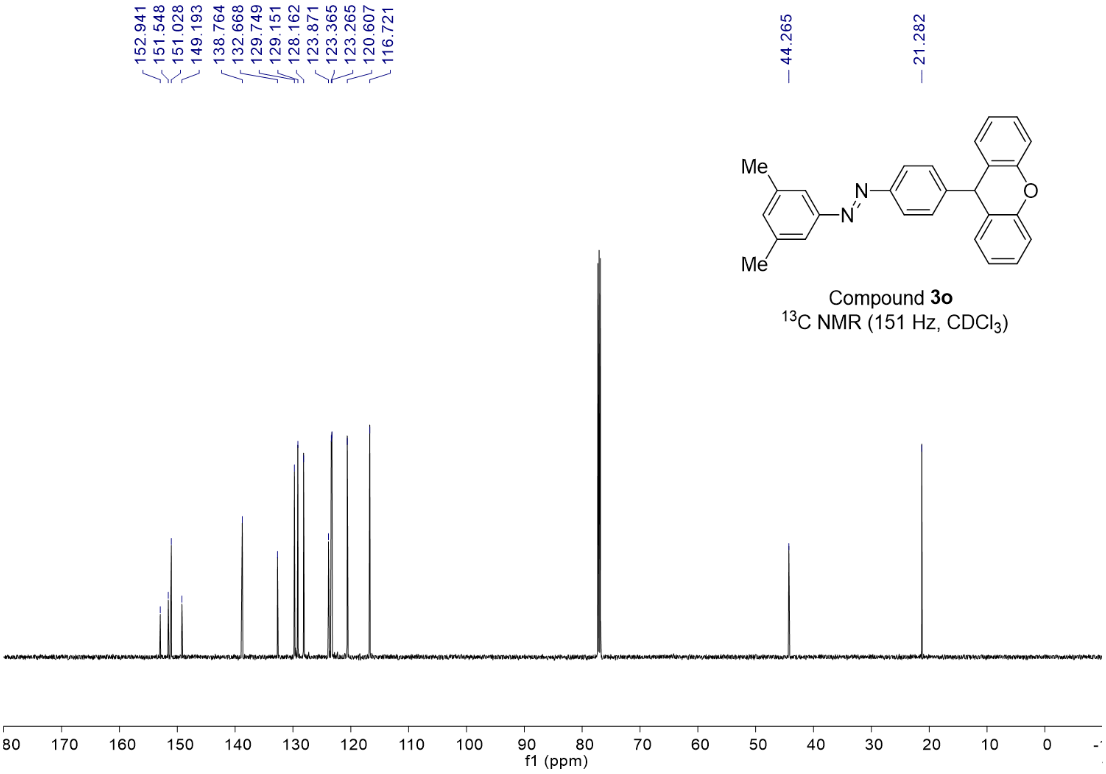

NMR spectra of (*E*)-1-(3-methyl-4-(9*H*-xanthen-9-yl)phenyl)-2-(m-tolyl)diazene (**3p**)

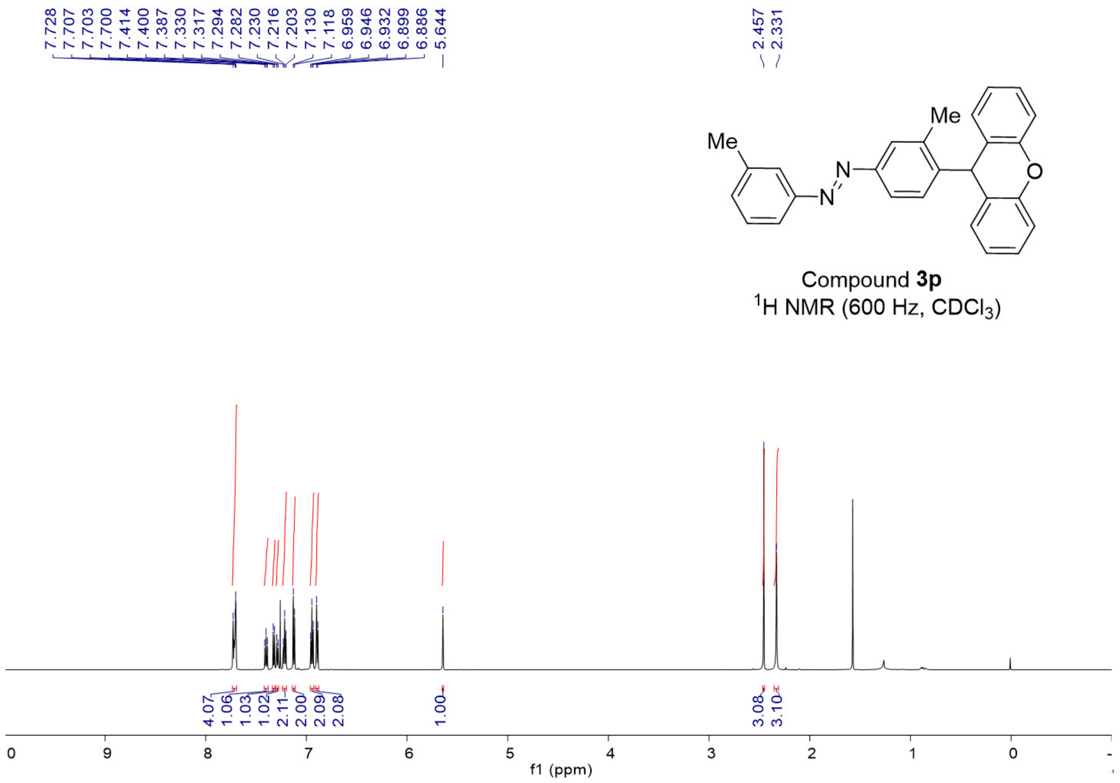

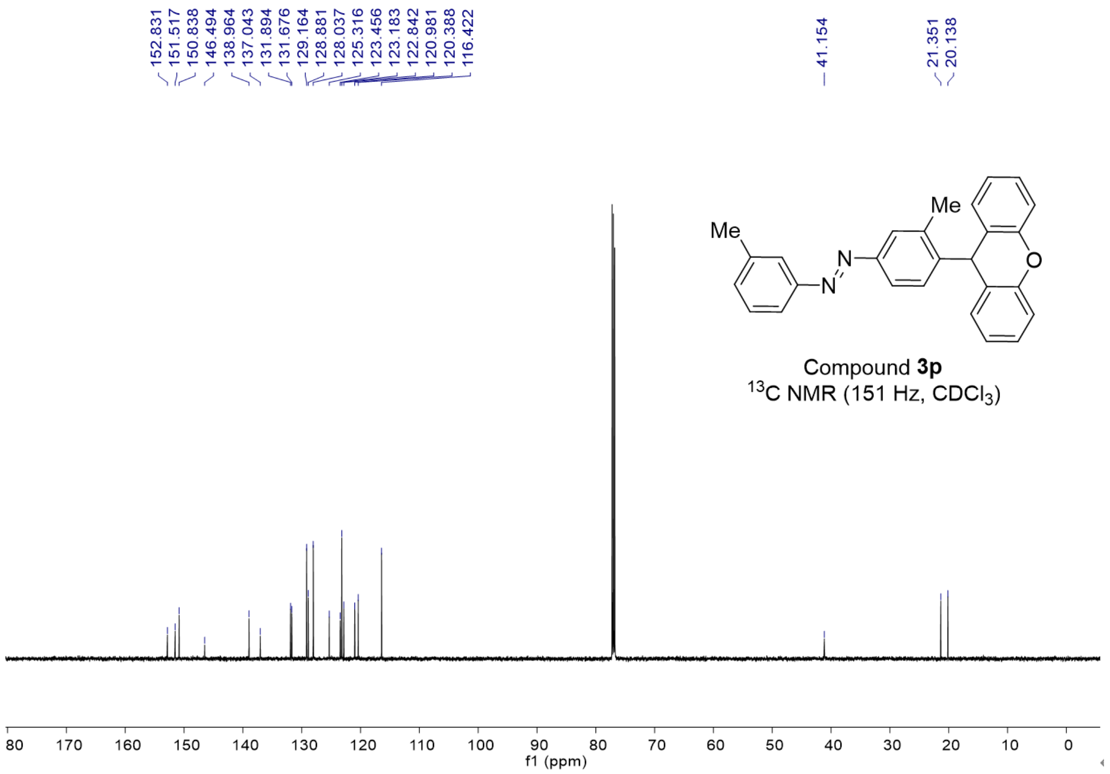

NMR spectra of (*E*)-1-(2-isopropyl-4-(9*H*-xanthen-9-yl)phenyl)-2-(2-isopropylphenyl)diazene (**3q**)

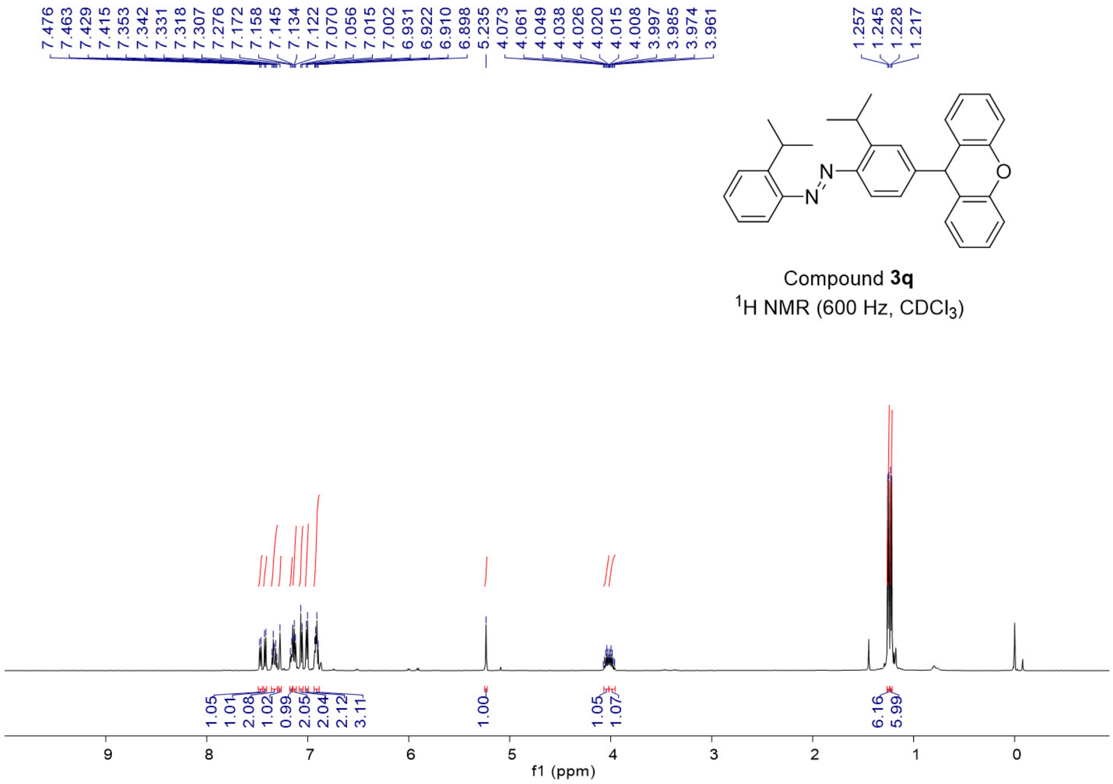

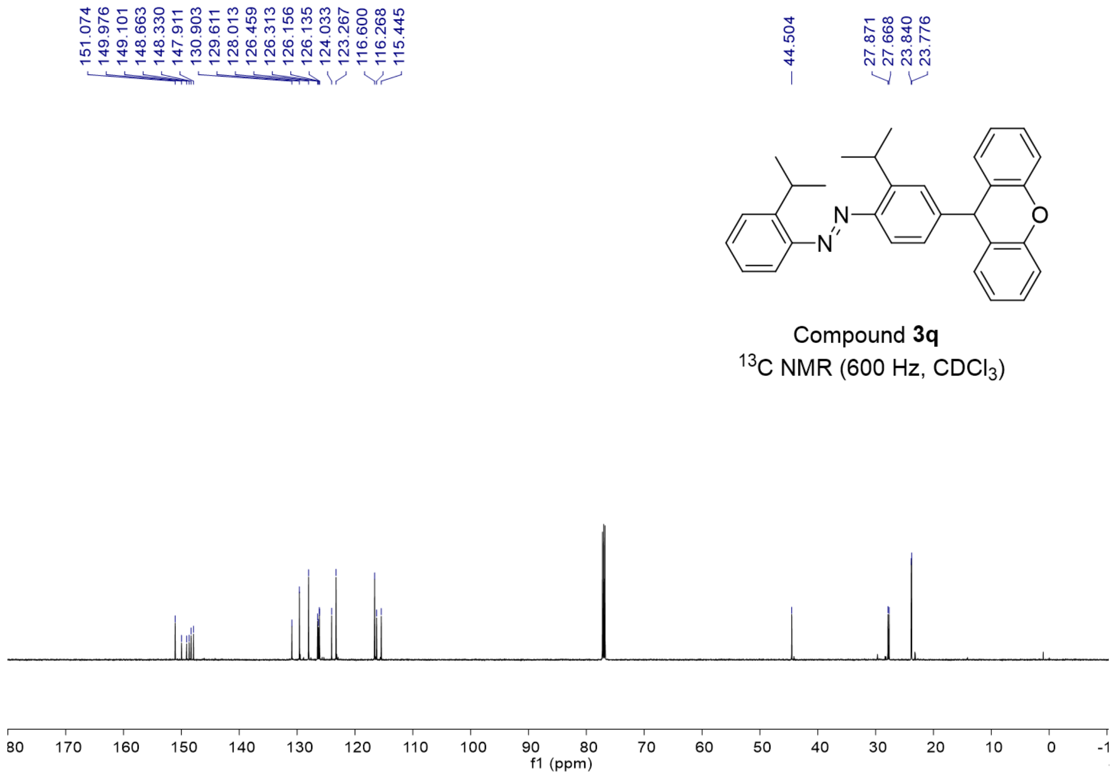

NMR spectra of (*E*)-1-(4-(9*H*-xanthen-9-yl)phenyl)-2-(3-methyl-4-(9*H*-xanthen-9-yl)phenyl)diazene (**4a**)

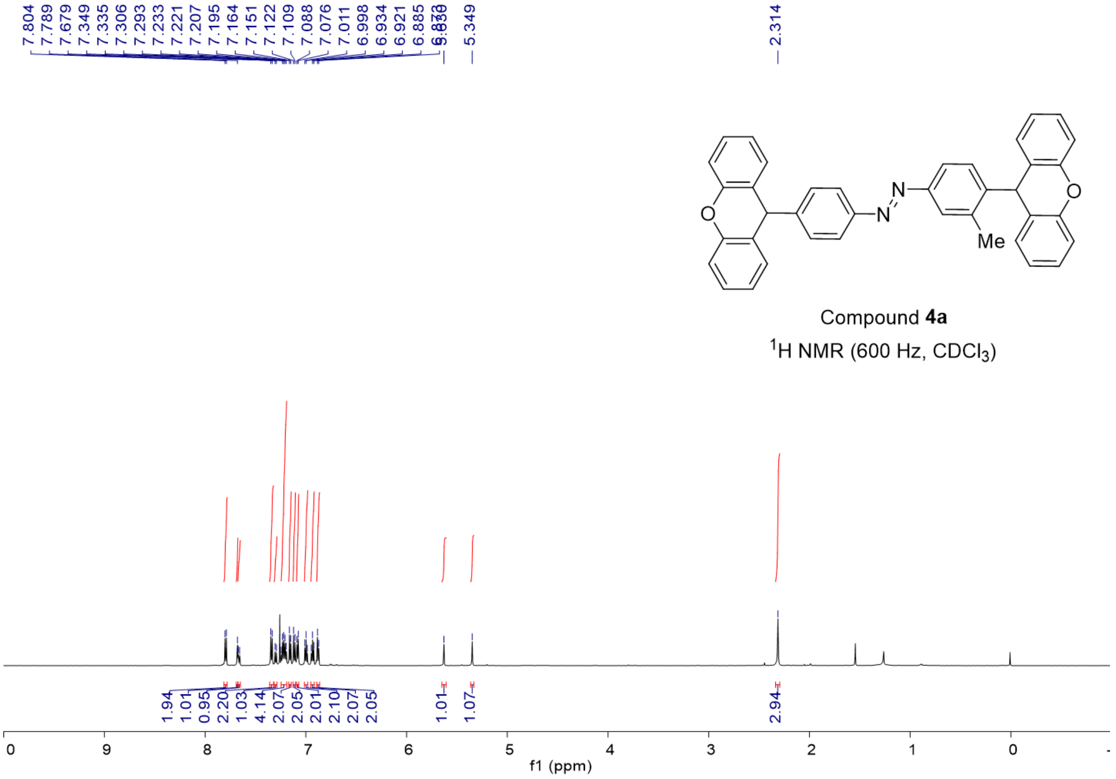

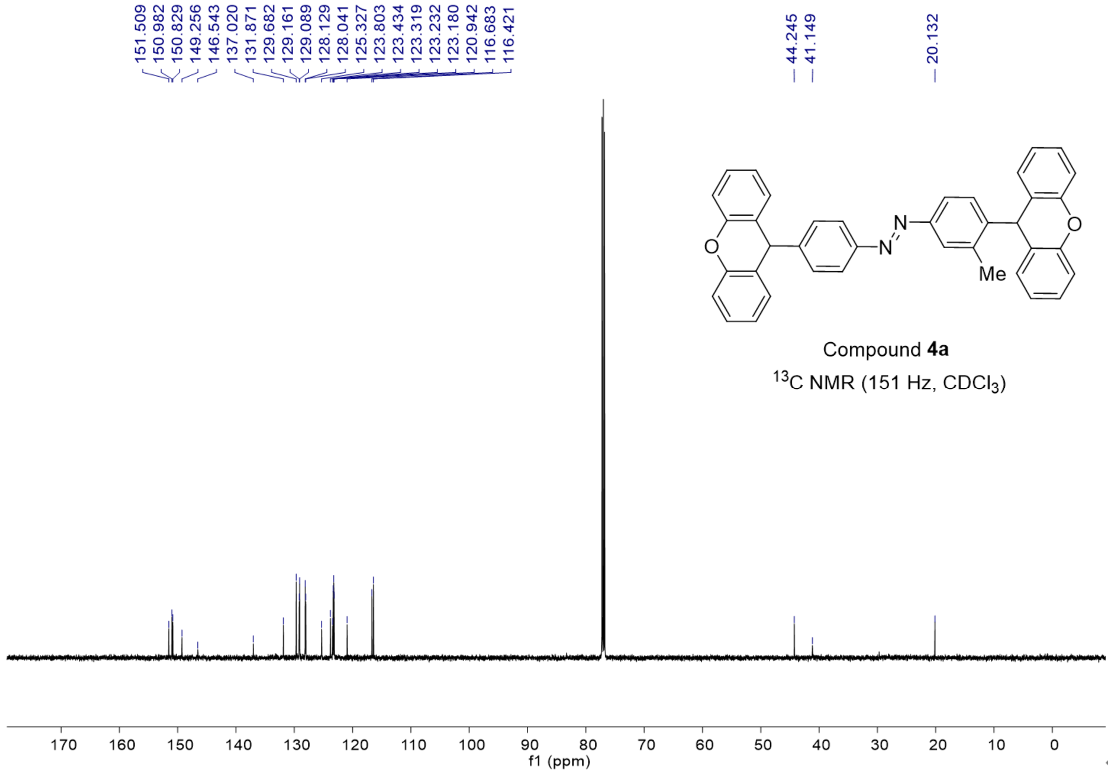

NMR spectra of (*E*)-1-(4-(9*H*-xanthen-9-yl)phenyl)-2-(2-isopropyl-4-(9*H*-xanthen-9-yl)phenyl)diazene (**4b**)

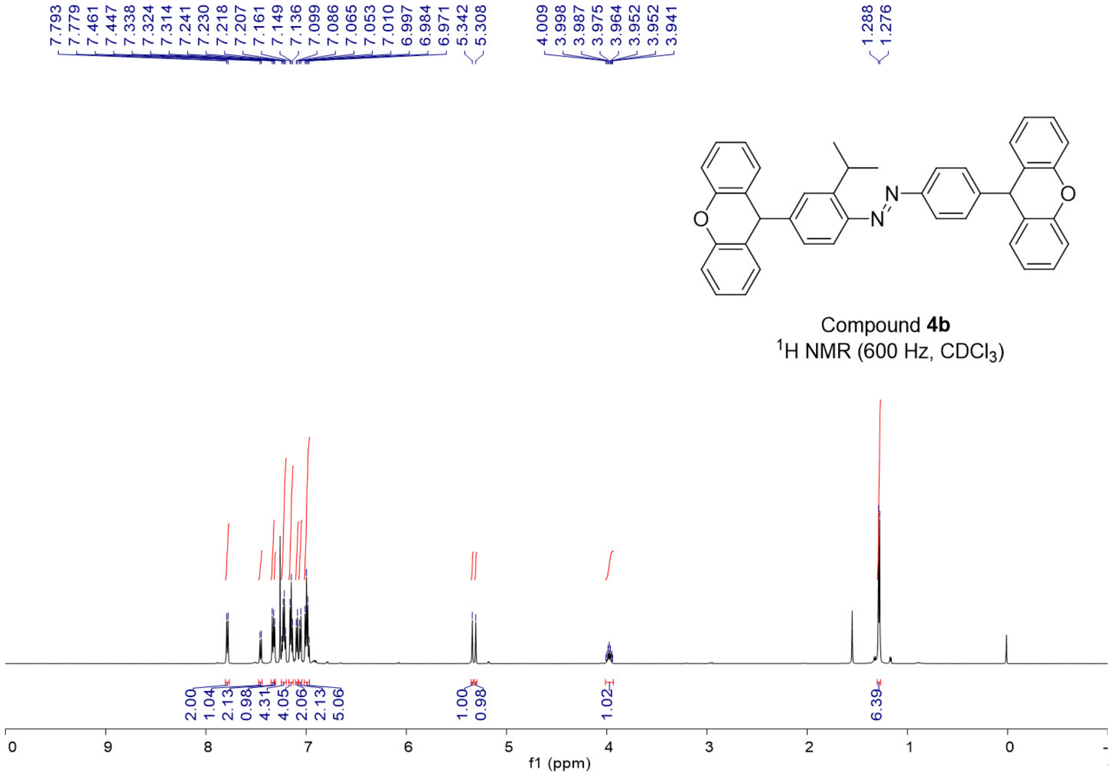

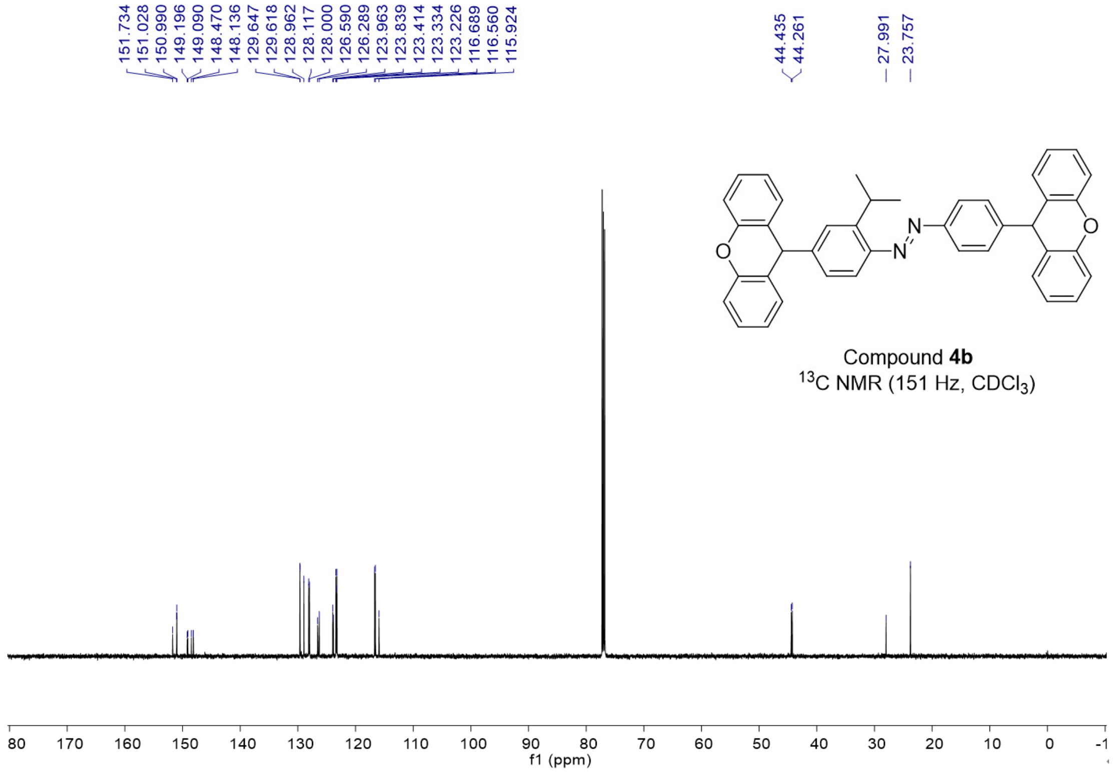

NMR spectra of (*E*)-1,2-bis(2-isopropyl-4-(9*H*-xanthen-9-yl)phenyl)diazene (**4c**)

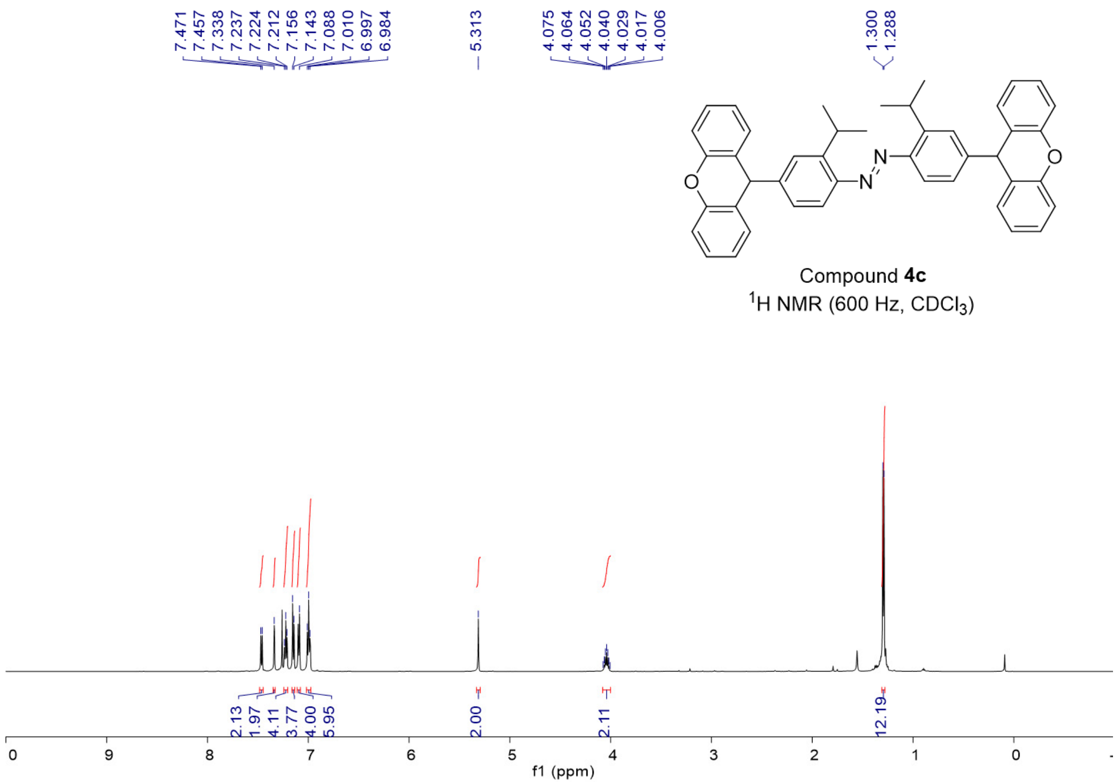

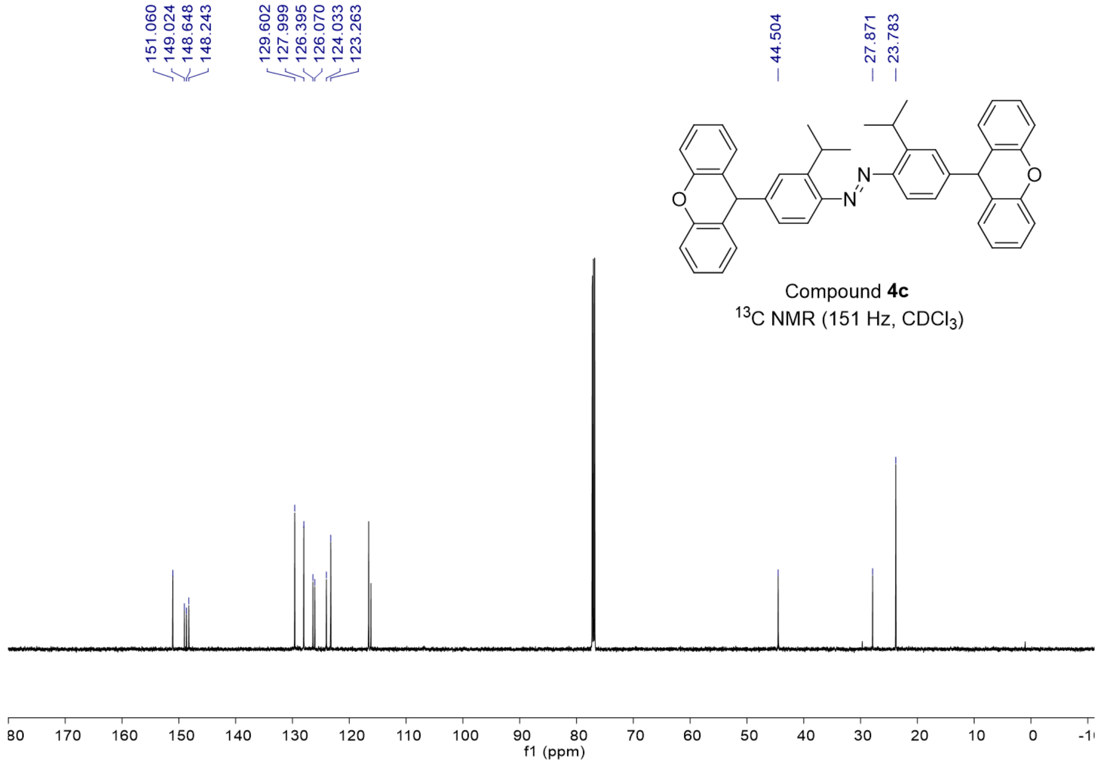

NMR spectra of (*E*)-1-(4-(2-methyl-9*H*-xanthen-9-yl)phenyl)-2-phenyldiazene (**5a**)

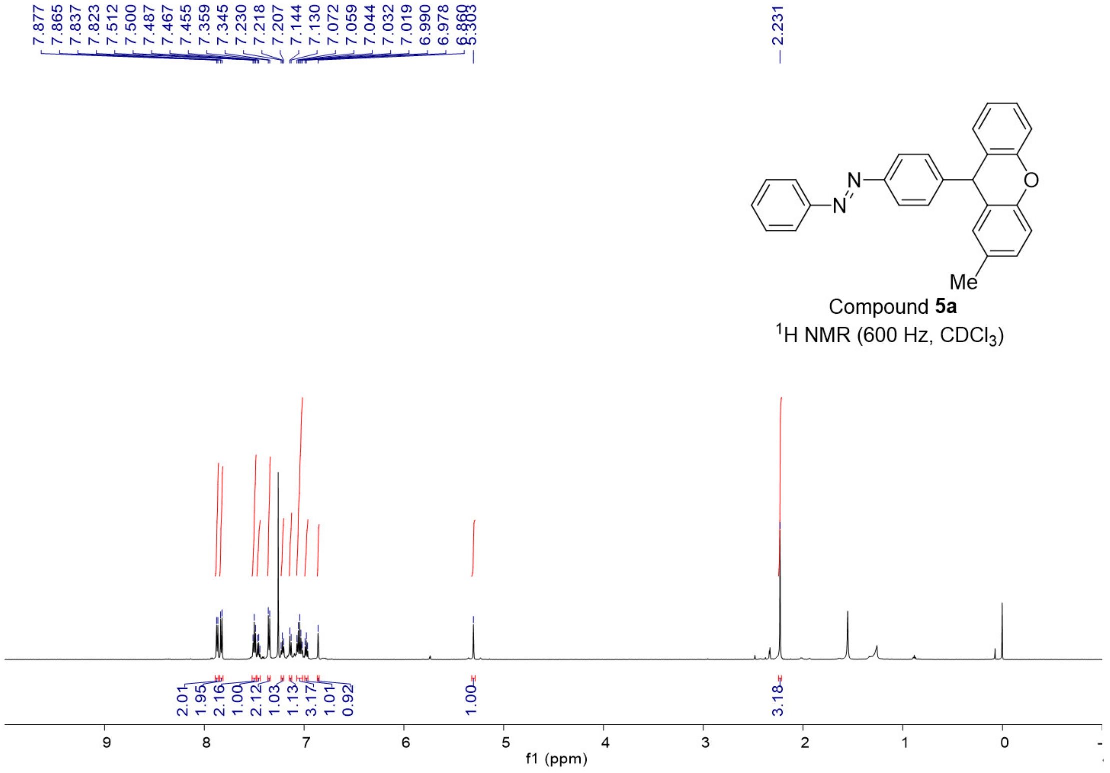

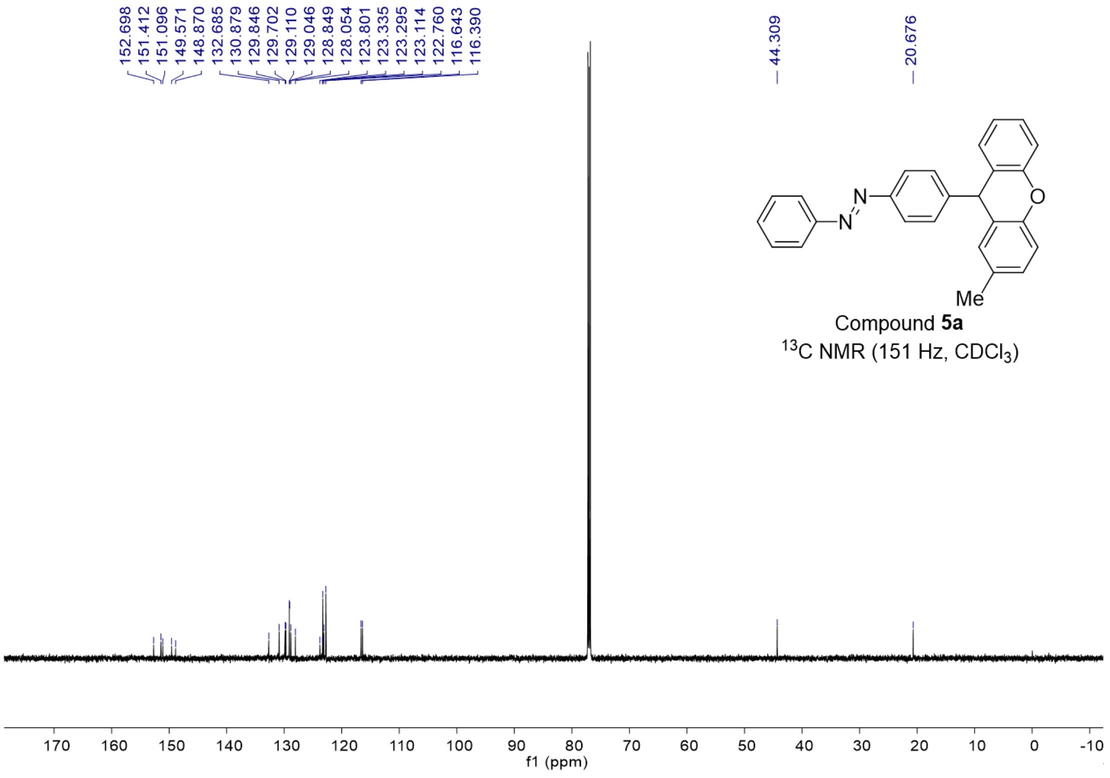

NMR spectra of (*E*)-1-(4-(4-methoxy-9*H*-xanthen-9-yl)phenyl)-2-phenyldiazene (**5b**)

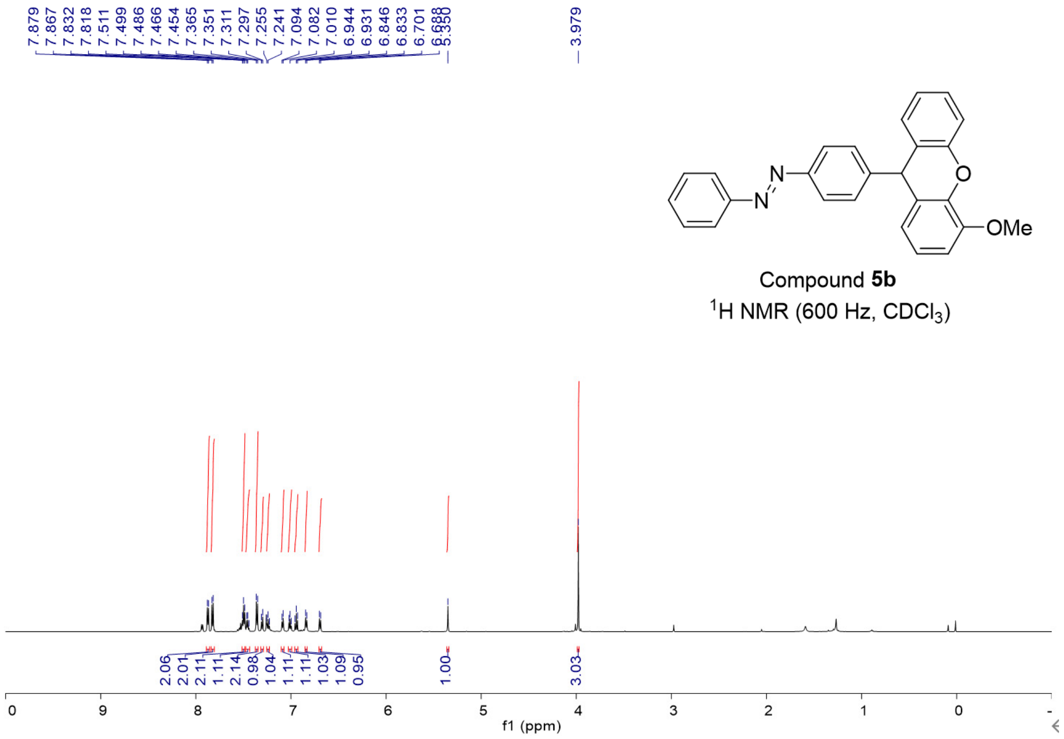

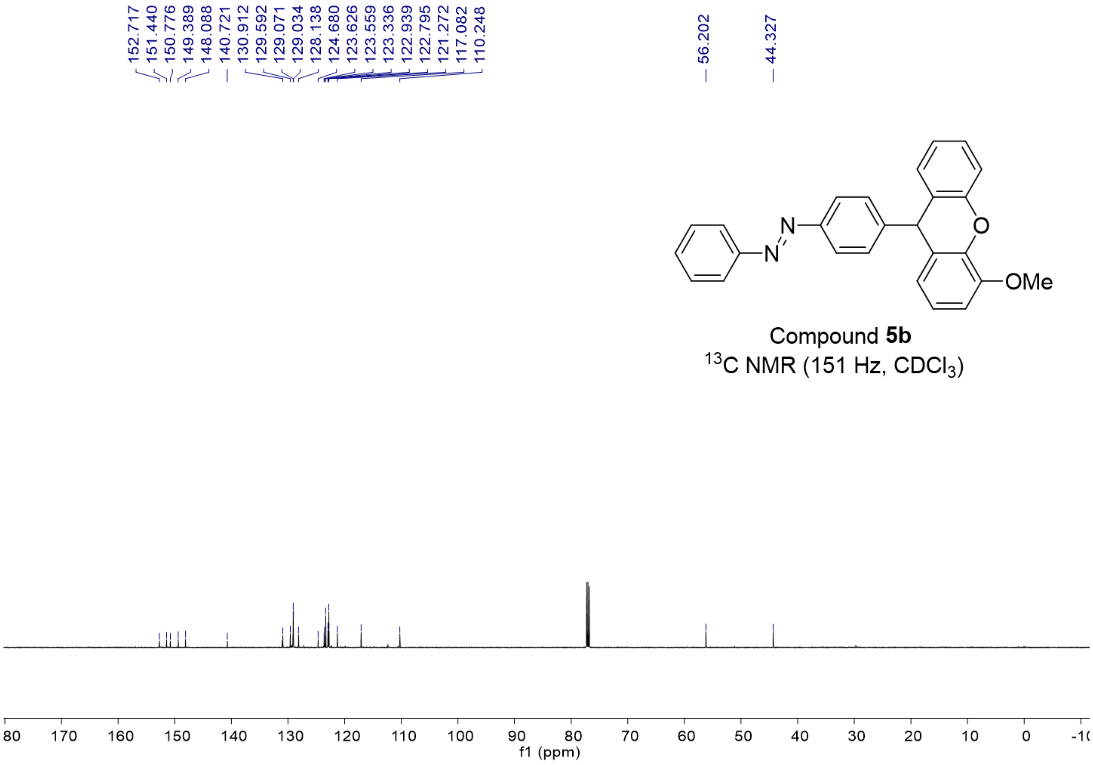

NMR spectra of (*E*)-1-phenyl-2-(4-(4-phenyl-9*H*-xanthen-9-yl)phenyl)diazene (**5c**)

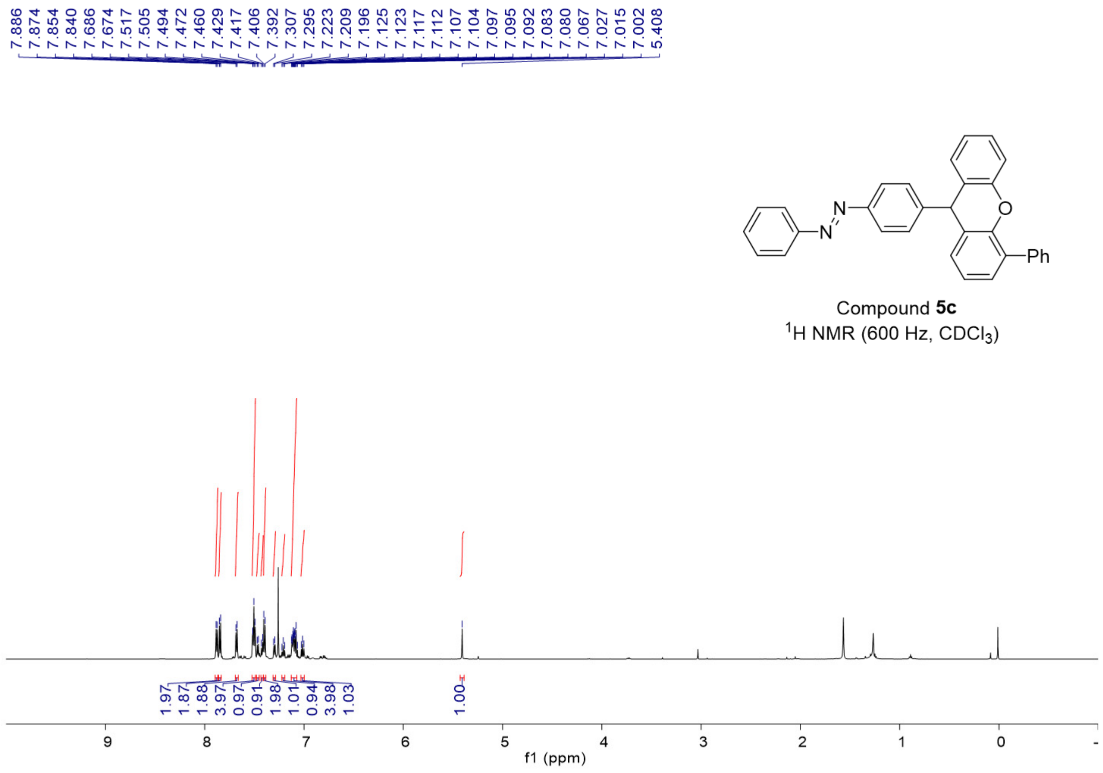

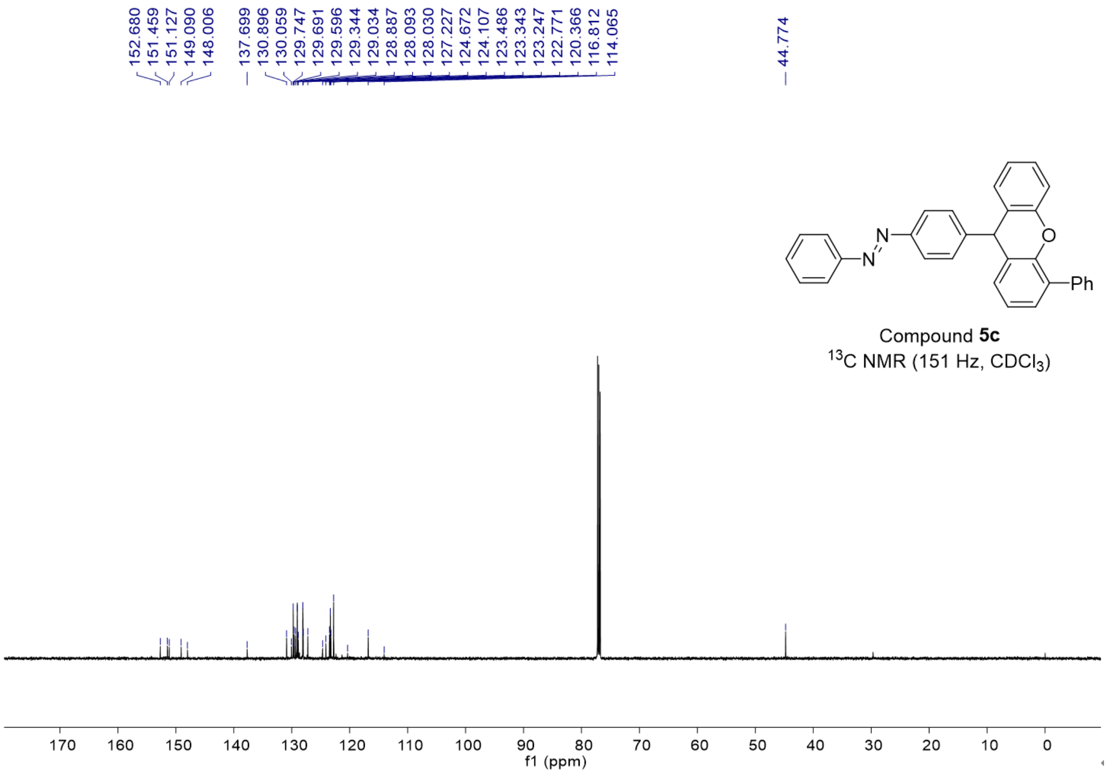

NMR spectra of (*E*)-1-(4-(12*H*-benzo[a]xanthen-12-yl)phenyl)-2-phenyldiazene (**5d**)

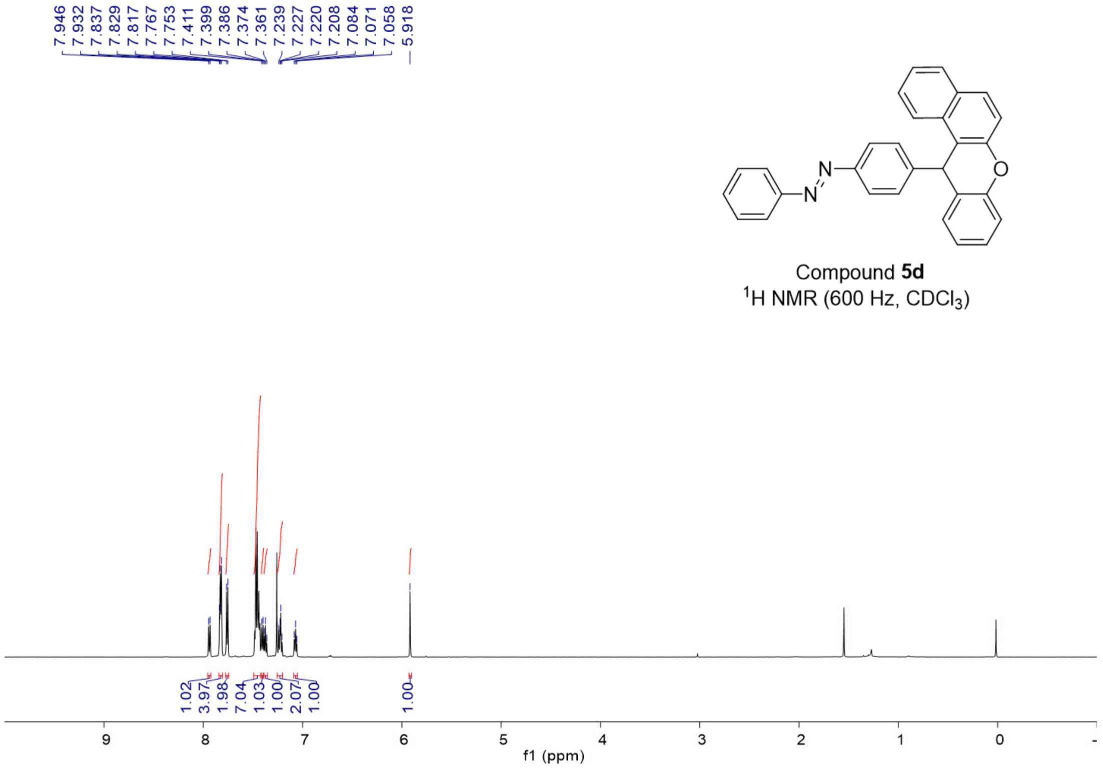

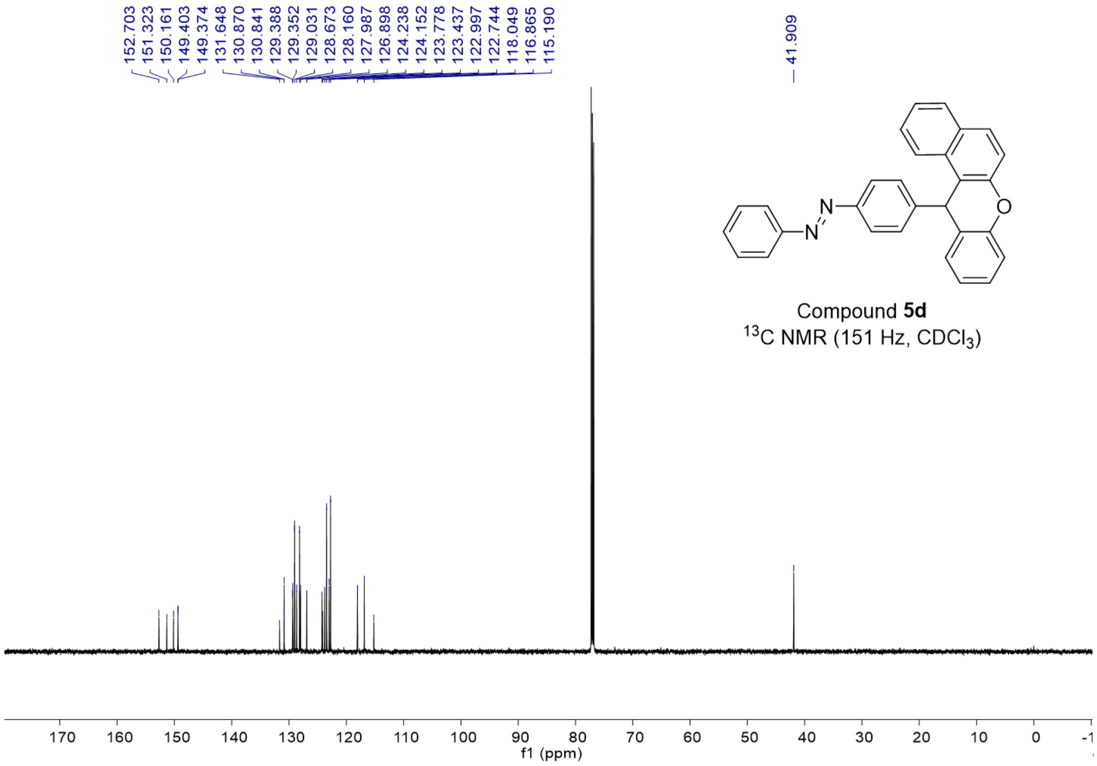

NMR spectra of (*E*)-1-(4-(7*H*-benzo[c]xanthen-7-yl)phenyl)-2-phenyldiazene (**5e**)

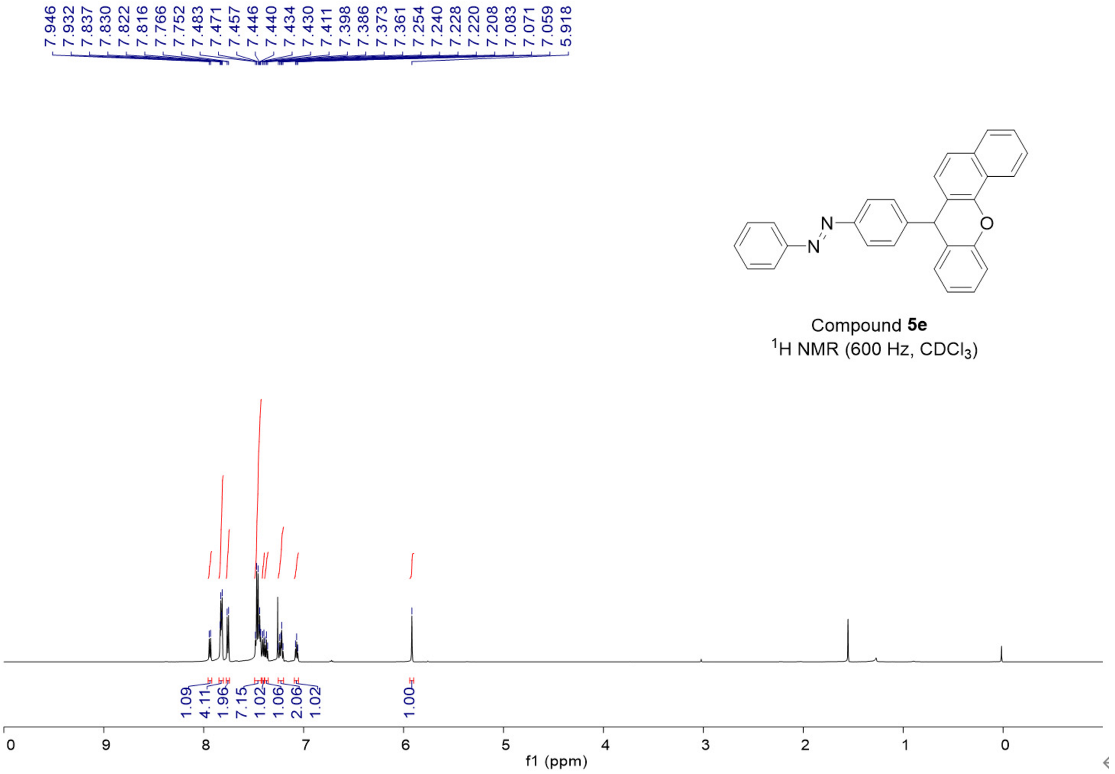

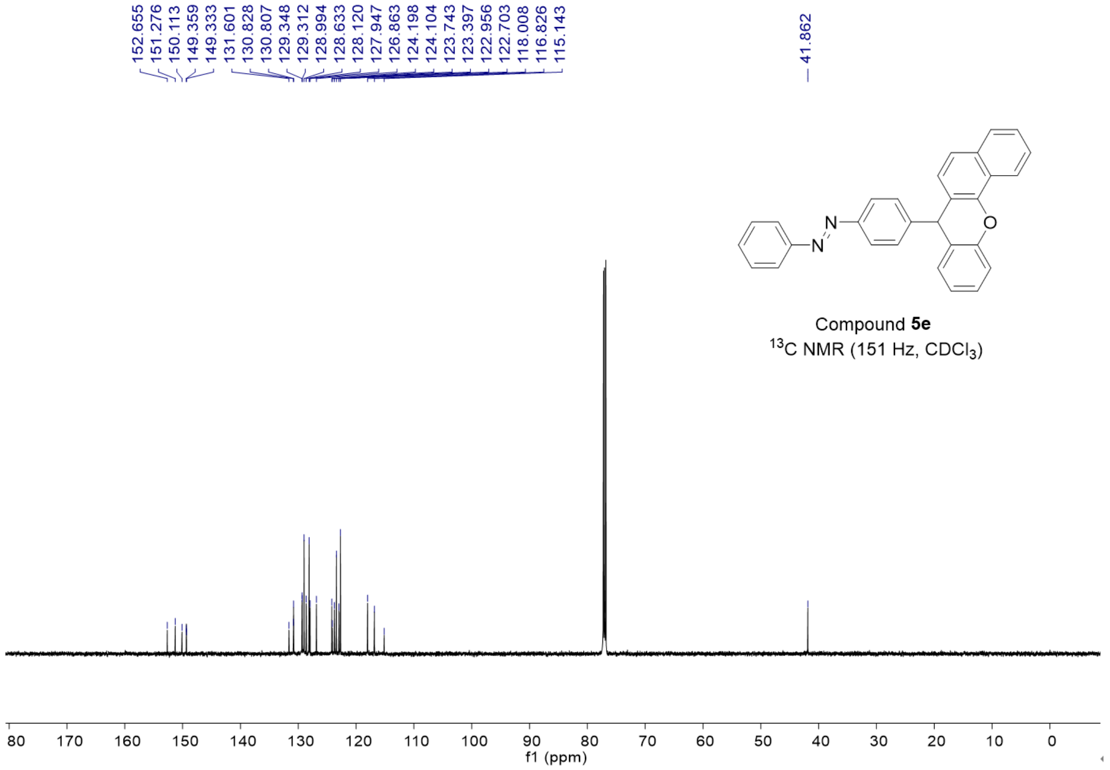

NMR spectra of (*E*)-1-(4-(10-methyl-12*H*-benzo[*a*]xanthen-12-yl)phenyl)-2-phenyl diazene (**5f**)

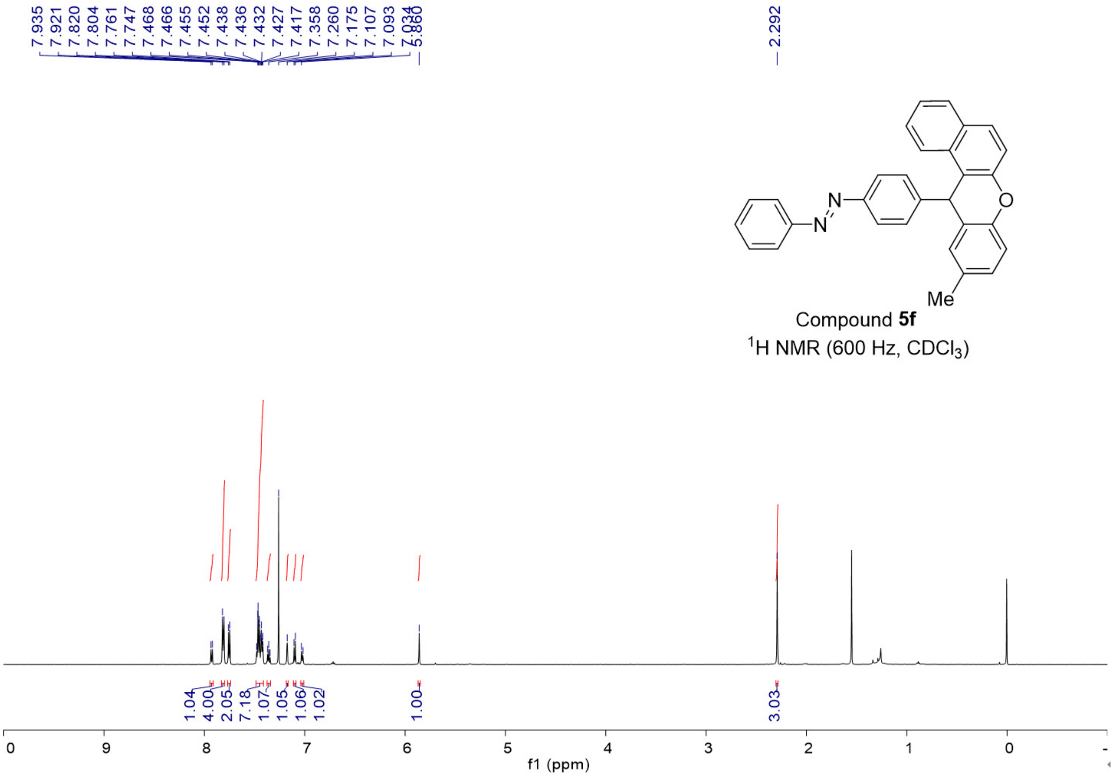

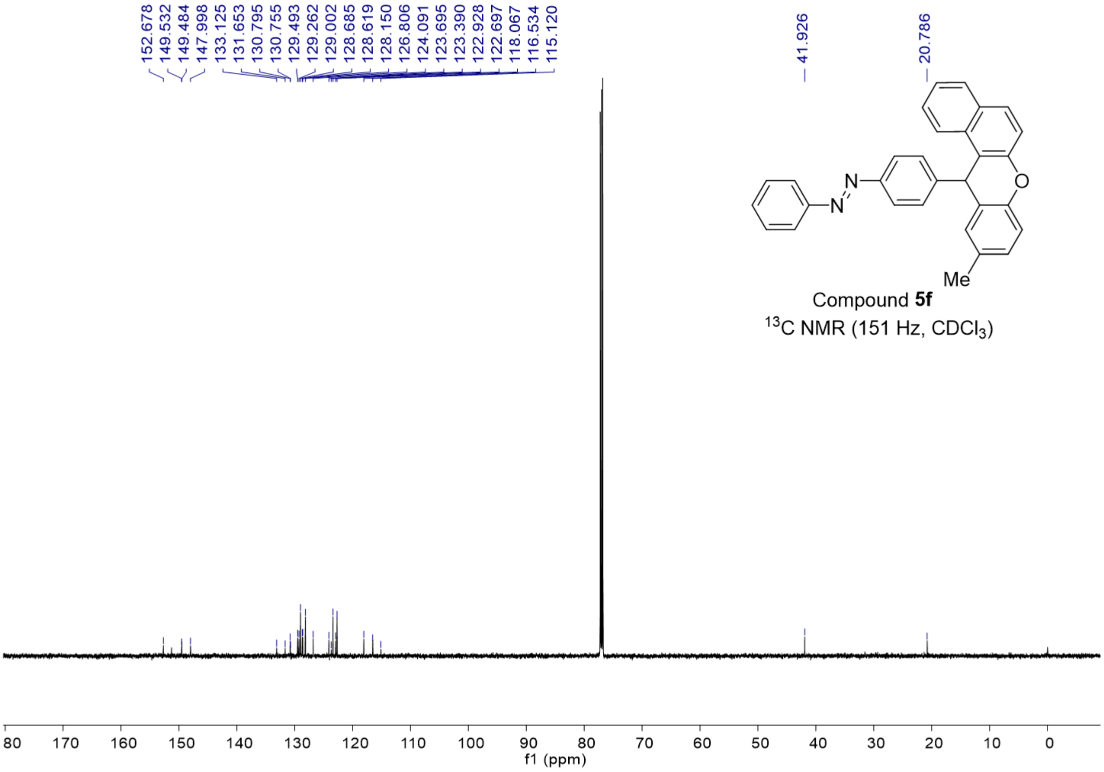

NMR spectra of (*E*)-1-(4-(9*H*-thioxanthen-9-yl)phenyl)-2-phenyldiazene (**5g**)

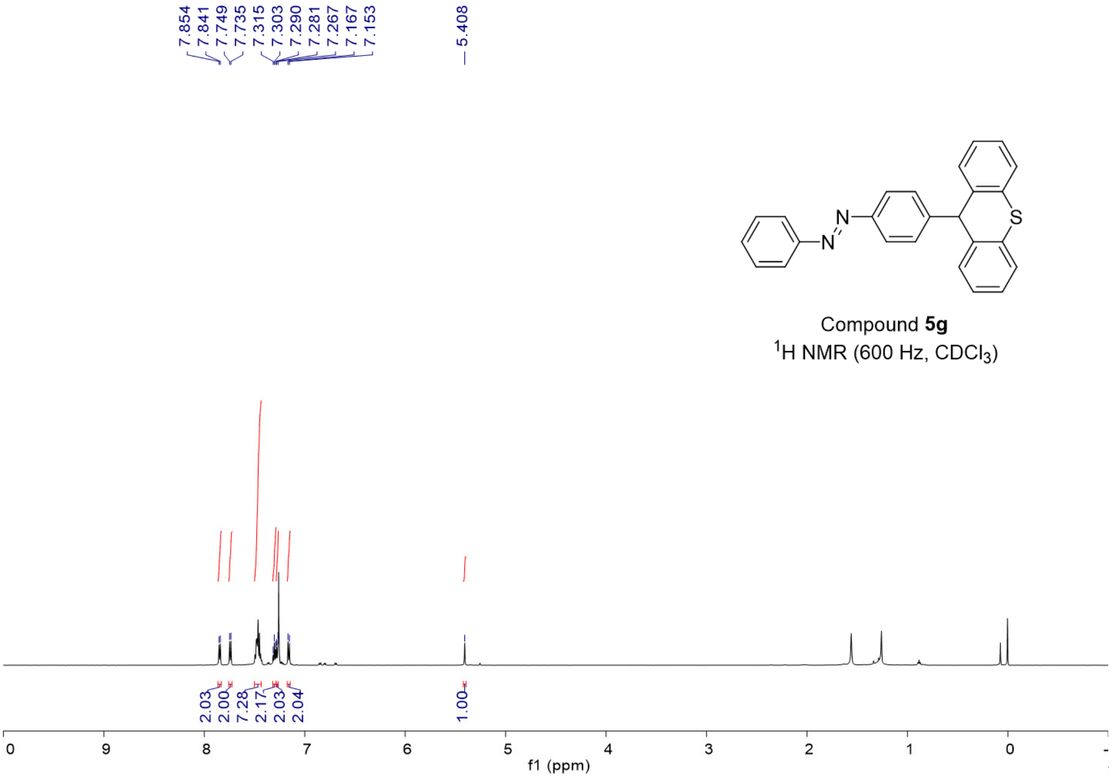

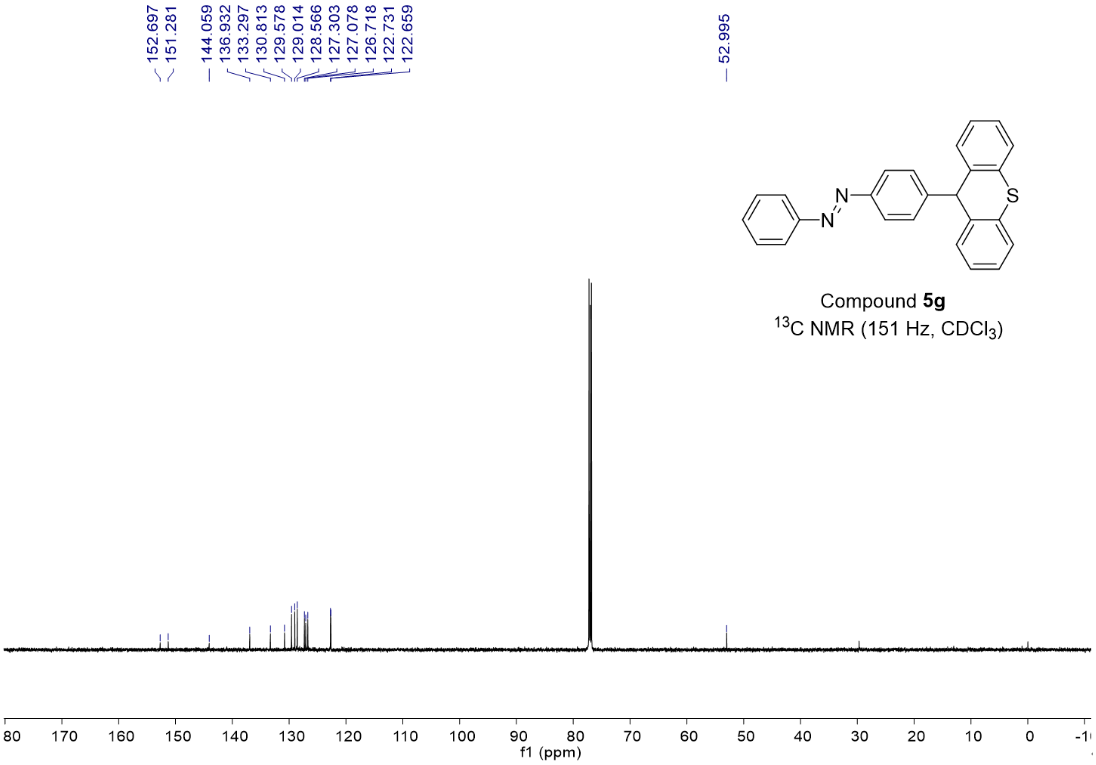

NMR spectra of (*E*)-1-(4-(2-methyl-9*H*-thioxanthen-9-yl)phenyl)-2-phenyldiazene (**5h**)

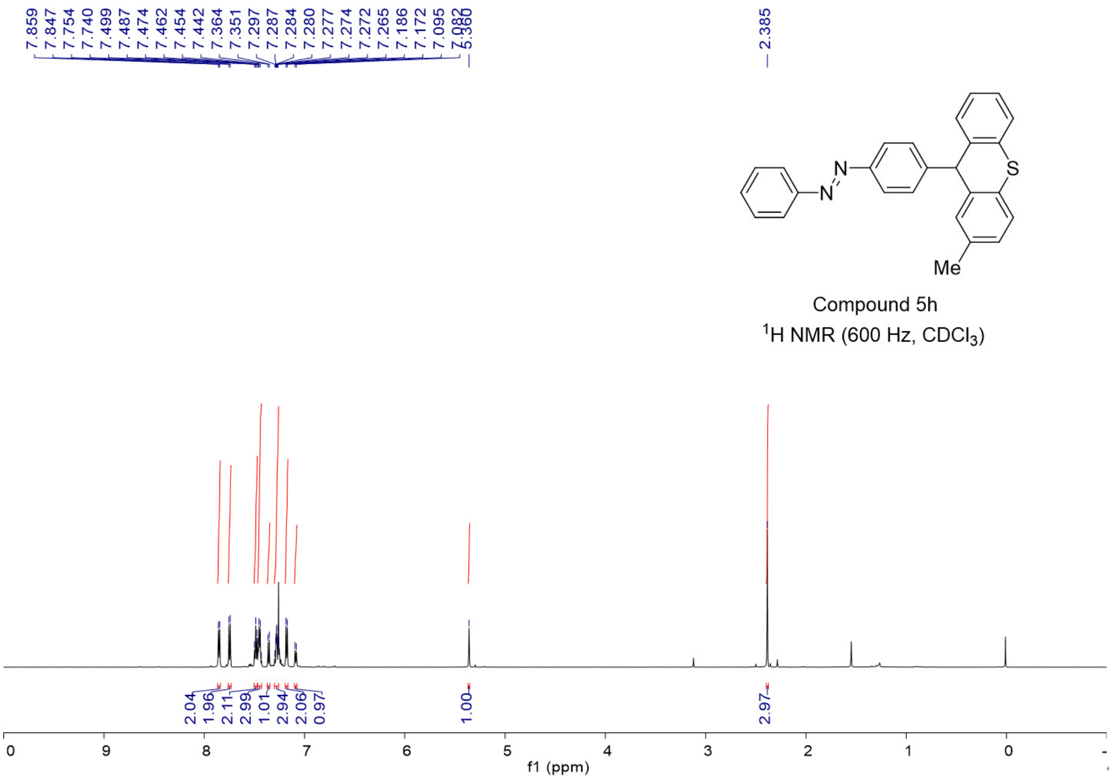

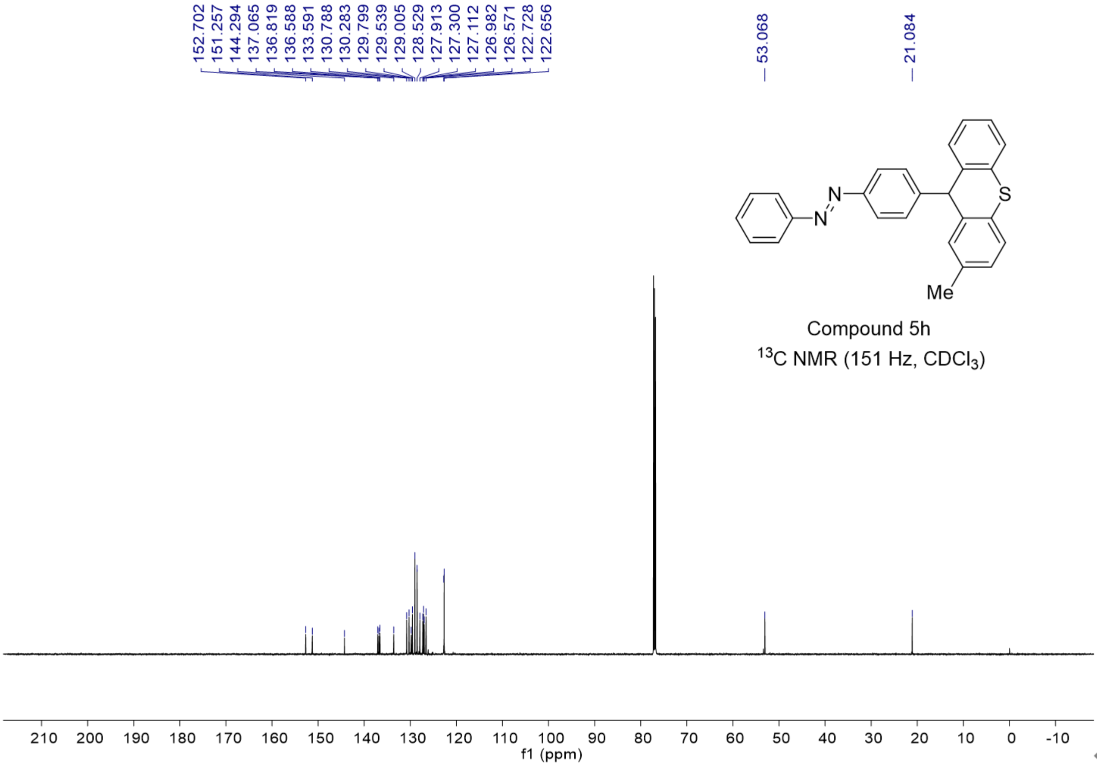

NMR spectra of (*E*)-1-(4-(2-chloro-9*H*-thioxanthen-9-yl)phenyl)-2-phenyldiazene (**5i**)

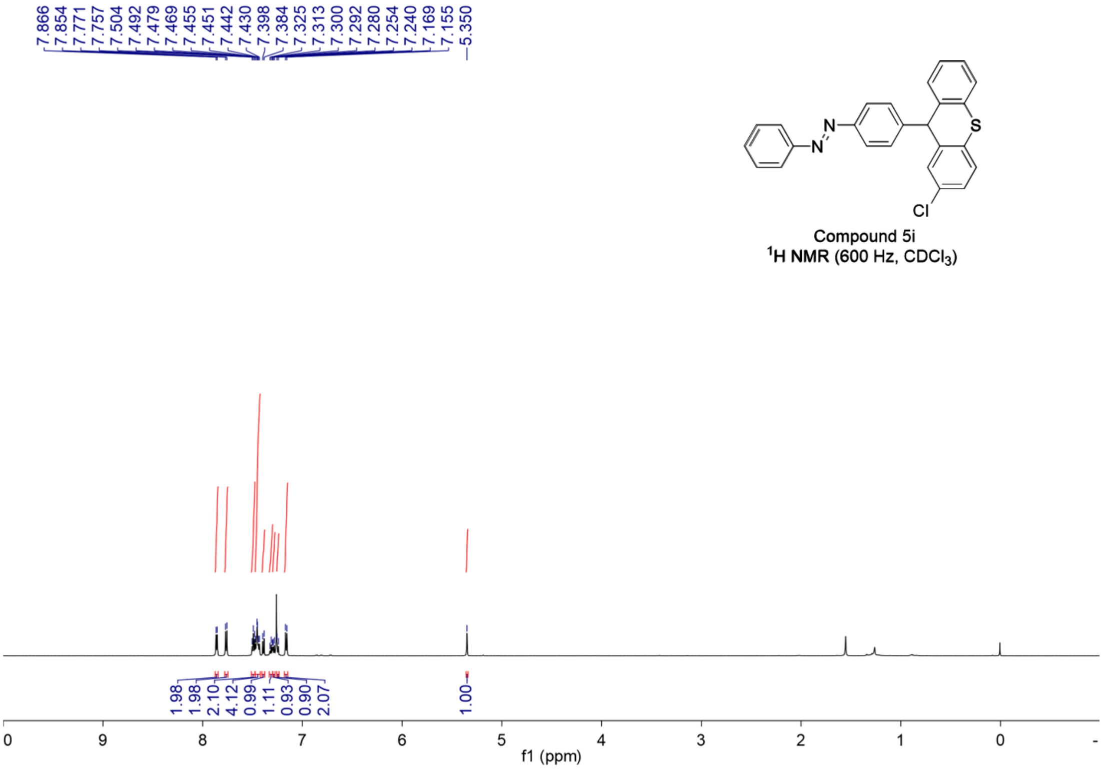

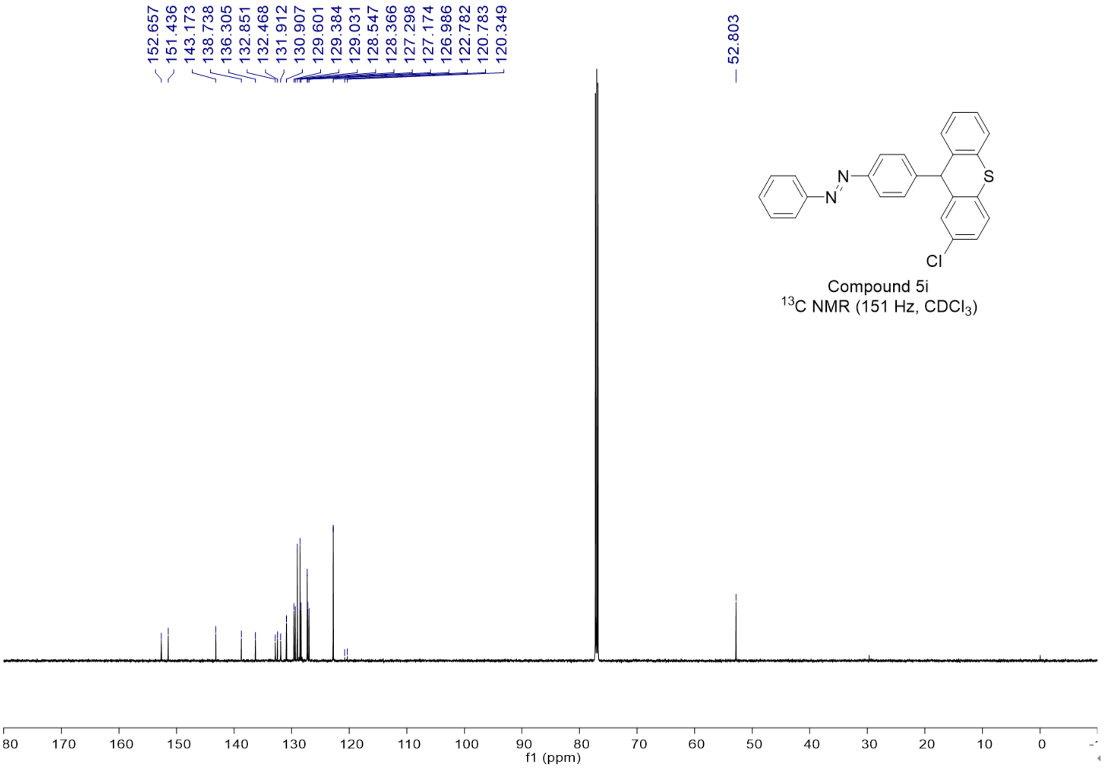

5. Crystallographic Data for 3d

The compound **3d** was crystalized over a solution of **3d** (80 mg) in CH_2Cl_2/petroleum (2 mL/2 mL) at room temperature. The mixed solvent spontaneously evaporates in open air to obtain the crystals of **3d**. Then the crystals were carefully collected and used for X-ray diffraction analysis. The crystal structure was further determined by Bruker D8 QUEST X-ray single crystal diffractometer. The CCDC number of **3d** is 2178297.

checkCIF/PLATON report

You have not supplied any structure factors. As a result the full set of tests cannot be run.

THIS REPORT IS FOR GUIDANCE ONLY. IF USED AS PART OF A REVIEW PROCEDURE FOR PUBLICATION, IT SHOULD NOT REPLACE THE EXPERTISE OF AN EXPERIENCED CRYSTALLOGRAPHIC REFEREE.

No syntax errors found. CIF dictionary Interpreting this report

Datablock: 1

Bond precision: C-C = 0.0020 A Wavelength=1.54178

Cell:	a=9.4464(1)	b=14.0973(2)	c=16.2854(3)
	alpha=90	beta=96.078(1)	gamma=90
Temperature:	298 K		

	Calculated	Reported
Volume	2156.52(6)	2156.51(6)
Space group	P 21/n	P 1 21/n 1
Hall group	-P 2yn	-P 2yn
Moiety formula	C28 H24 N2 O	4(C28 H24 N2 O)
Sum formula	C28 H24 N2 O	C112 H96 N8 O4
Mr	404.49	1617.96
Dx,g cm-3	1.246	1.246
Z	4	1
Mu (mm-1)	0.590	0.590
F000	856.0	856.0
F000’	858.33	
h,k,lmax	11,17,20	11,17,20
Nref	4499	4120
Tmin,Tmax	0.932,0.943	0.920,1.000
Tmin’	0.889	

Correction method= # Reported T Limits: Tmin=0.920 Tmax=1.000
AbsCorr = MULTI-SCAN

Data completeness= 0.916 Theta(max)= 75.811

R(reflections)= 0.0420(3595) wR2(reflections)= 0.1228(4120)

S = 1.047 Npar= 282

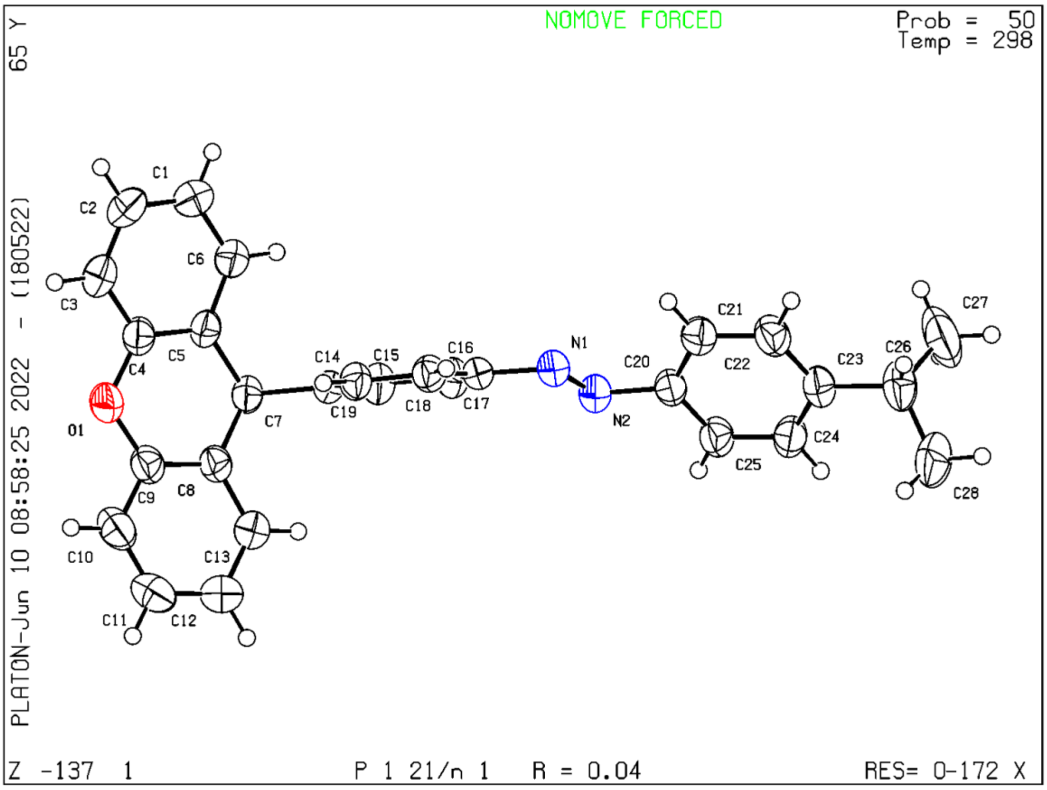

Figure S3 X-ray structure of **3d** (ORTEP diagram with ellipsoid contour 50% probability)

6. Determination of Faradaic Efficiency

$$(1)\ \text{F. E. (\%)} = \frac{\text{n} \times \text{F} \times \text{mol of product or intermediate formed}}{\text{acculumated charge (C)}} \times 100\ \%$$

$$\text{F. E. (\%)} = \frac{2 \times 96485\ \text{C mol}^{-1} \times 0.3\ \text{mmol} \times 10^{-3} \times 76\%}{9\ \text{mA} \times 10^{-3} \times 4\ \text{h} \times 3600} \times 100\ \%$$

$$= 33.9\ \%$$

The F.E. (%) of the product **3a** was calculated by (1). The F.E. is the proportion of electrons consumed in each electrochemical reaction of the total applied charge and represents the selectivity of the electrochemical system for each reaction. In Eq (1), F is the Faradaic constant (96485 C mol^{-1}), and n is the number of electrons required for the production of products. The yield is the proportion of reactant converted to target product.
